# Neolignans and Norlignans from Insect Medicine *Polyphaga plancyi* and Their Biological Activities

**DOI:** 10.1007/s13659-020-00262-0

**Published:** 2020-09-02

**Authors:** Hong-Jie Zhu, Te Xu, Yong-Ming Yan, Zheng-Chao Tu, Yong-Xian Cheng

**Affiliations:** 1 School of Pharmaceutical Sciences, Shenzhen University Health Science Center, Shenzhen, 518060 People’s Republic of China; 2 State Key Laboratory of Phytochemistry and Plant Resources in West China, Kunming Institute of Botany, Chinese Academy of Sciences, Kunming, 650201 People’s Republic of China; 3 International Cooperative Laboratory of Traditional Chinese Medicine Modernization and Innovative Drug Development of Chinese Ministry of Education (MOE), College of Pharmacy, Jinan University, Guangzhou, 510632 People’s Republic of China; 4 Drug Discovery Pipeline & Guangdong Provincial Key Laboratory of Biocomputing, Guangzhou Institutes of Biomedicine and Health, Guangzhou, 510530 People’s Republic of China

**Keywords:** *Polyphaga plancyi*, Insect medicine, Lignans, Renal protection, Anticoagulant activity

## Abstract

**Abstract:**

Ten neolignans or norlignans (**1**–**10**) including eight new compounds were isolated from the whole bodies of *Polyphaga plancyi* Bolivar. Their structures were identified by spectroscopic data. Compounds **3**, **4**, **8**, and **9** are racemates indicated by chiral HPLC analysis. Chiral separation followed by ECD calculations allowed to clarify the absolute configurations of all the antipodes. All the new compounds were evaluated for their biological properties toward extracellular matrix in rat renal proximal tubular cells, human cancer cells (K562, A549, and Huh7), EV71, ROCK2, JAK3, DDR1, and coagulation.

**Graphic Abstract:**

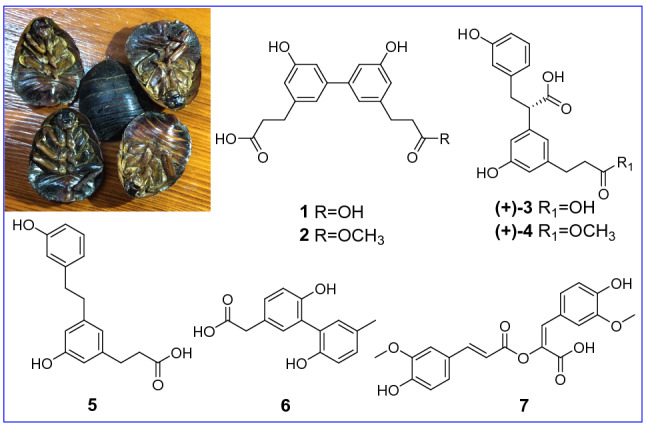

**Electronic supplementary material:**

The online version of this article (10.1007/s13659-020-00262-0) contains supplementary material, which is available to authorized users.

## Introduction

Insects are a special group of existence on the earth. Facing the changing environment, they have strong adaptability, productivity and continuity. They seem small and insignificant, but the largest number of living beings on the planet, and their presence has created a huge molecular libraries [1, 2]. The tenacious vitality of insects is highly likely to be related to their unique molecular mechanisms [2], which provides us a confidence to track their bioactive molecules.

**Fig. 1:**
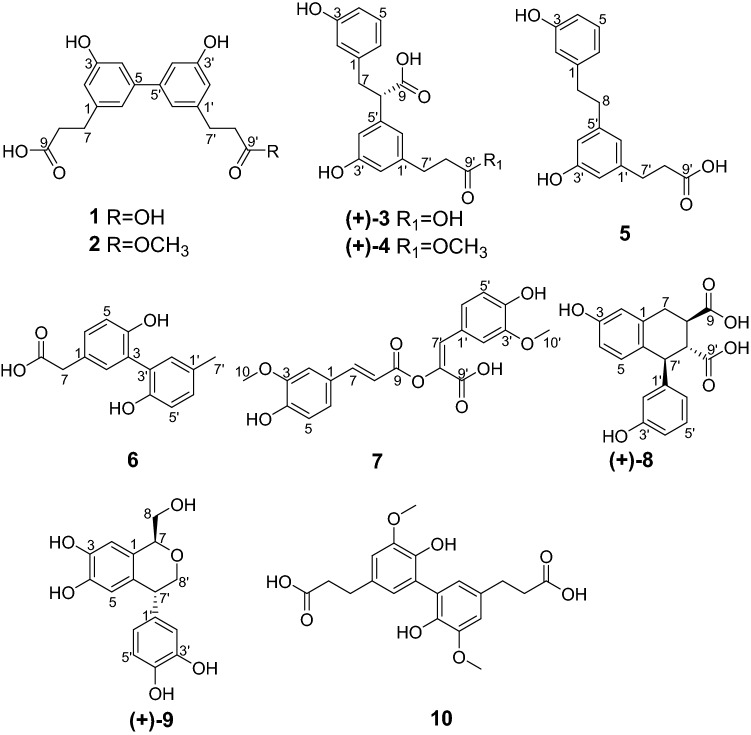
The chemical structures of compounds **1**–**10**

In China, the insect *Polyphaga plancyi* Bolivar has been used for promoting blood circulation [3]. Whereas, so far there have few reports about its chemical composition [4]. Previous studies revealed the significance of structurally novel non-peptide small molecules (NPSMs) [5–11], inspiring our further interest on insect chemistry. As far as the title species was concerned, eight new compounds and two known compounds belonging to neolignans and norlignans were isolated in this study (Fig. 1). Chiral HPLC separation was used to afford optically active isomers. Subsequent absolute configuration clarification was aided by electronic circular dichroism (ECD) calculations. In addition, the biological activities of all the new compounds were assessed using multiple assays.

## Results and Discussion

### Structure Elucidation of the Compounds

**Fig. 2:**
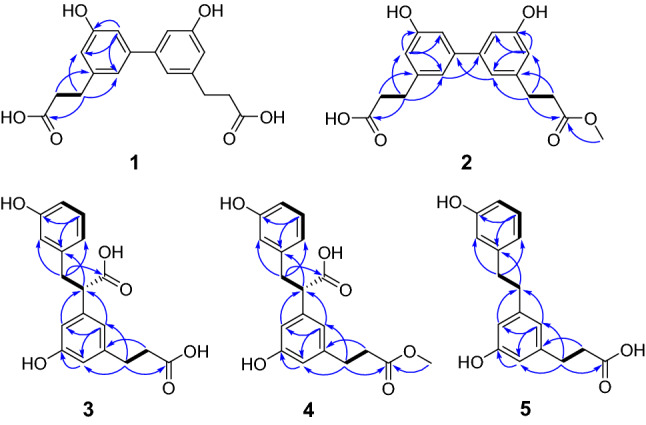
^1^H–^1^H COSY (black bond) and key HMBC (blue arrow) correlations of **1**–**5**

Plancyin A (**1**) has the molecular formula C_18_H_18_O_6_ deduced from analysis of its HRESIMS ([M−H]^−^, *m/z* 329.1040, calcd 329.1031), ^13^C NMR, and DEPT spectra, indicating 10 degrees of unsaturation. The ^1^H NMR spectrum (Table 1) of **1** indicates the presence of three aromatic/olefinic protons at *δ*_H_ 6.63 (brs, H-2), 6.82 (brs, H-4), 6.90 (brs, H-6), indicating the presence of a 1,3,5-trisubstituted benzene ring. The ^13^C NMR and DEPT spectra show 9 carbons ascribed to two methylene, three *sp*^2^ methine, and four quaternary carbons (one carbonyl, three olefinic including one oxygenated). Based on the molecular formula, we judged that compound **1** is a completely symmetrical structure. The ^1^H–^1^H COSY spectrum (Fig. 2) shows correlations of H-7/H-8. The HMBC correlations of H-7/C-2, C-6, C-9 (*δ*_C_ 176.9), H-8/C-1, and H-4/C-2, C-3 (*δ*_C_ 158.8), C-6 allow to establish half the structure fragment of **1**. Taken together, the structure of **1** was established as shown.

**Table 1:** ^1^H (600 MHz) and ^13^C NMR (150 MHz) data of **1**–**3** (*δ* in ppm, *J* in Hz, in methanol-*d*_4_)

No.	**1**		**2**		**3**	
	*δ*_H_	*δ*_C_	*δ*_H_	*δ*_C_	*δ*_H_	*δ*_C_
1		144.0		144.1		142.5
2	6.63, brs	115.1	6.63, brs	115.2^b^	6.59, brs	116.9
3		158.8		158.8		158.2
4	6.82, brs	112.8	6.81^a^, brs	112.8^c^	6.57, dd, 8.1, 1.9	114.1
5		144.1		144.1^d^	7.01, t-like	130.2
6	6.90, brs	119.5	6.90, brs	119.4	6.61, overlap	121.3
7	2.88, t, 7.6	32.1	2.89, t, 7.5	32.3	3.23, dd, 13.6, 8.8	40.8
					2.84, dd, 13.6, 6.6	
8	2.61, t, 7.6	36.8	2.60, t, 7.5	37.1	3.68, t-like	55.3
9		176.9		177.4		177.7
1′		144.0		143.8		143.9
2′	6.63, brs	115.1	6.61, brs	115.1^b^	6.54, brs	115.0
3′		158.8		158.8		158.9
4′	6.82, brs	112.8	6.80^a^, brs	112.9^c^	6.62, overlap	113.7
5′		144.1		144.0^d^		142.4
6′	6.90, brs	119.5	6.88, brs	119.4	6.65, brs	120.5
7′	2.88, t, 7.6	32.1	2.89, t, 7.5	32.0	2.80, t, 7.7	32.3
8′	2.61, t, 7.6	36.8	2.64, t, 7.5	36.6	2.52, t, 7.7	37.1
9′		176.9		175.2		177.4
10′			3.65, s	52.1		

^a,b,c,d^The same symbols at the same column are interchangeable

Plancyin B (**2**) has a molecular formula of C_19_H_20_O_6_ derived from its HREIMS (*m/z* 343.1193 [M−H]^−^, calcd for 343.1187), ^13^C NMR, and DEPT spectra, having 10 degrees of unsaturation. The ^1^H and ^13^C NMR data of **2** resemble those of **1**, differing in that the additional existence of a methyl in **2**. The HMBC correlation of H-10′ (*δ*_H_ 3.65, s)/C-9′ (*δ*_C_ 175.2) indicates the connection position of the methyl group as shown. With these in hand, the structure of **2** was readily determined.

**Fig. 3:**
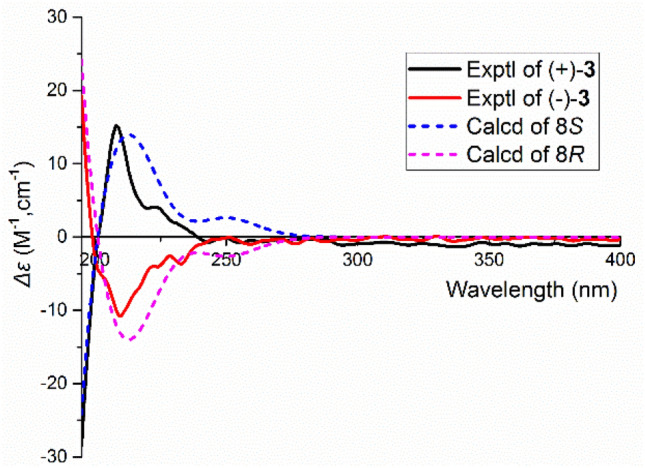
Comparison of calculated ECD spectra for (8*S*)-**3** and (8*R*)-**3** with the experimental spectra of (+)**-3** and (−)**-3** in MeOH. *σ* = 0.3 eV; shift =  − 10 nm

Plancyin C (**3**) possesses a molecular formula C_18_H_18_O_6_ (10 degrees of unsaturation) deduced from its negative HRESIMS at *m/z* 329.1036 [M−H]^−^ (calcd for 329.1031) and NMR data. The ^1^H NMR spectrum (Table 1) indicates the presence of seven aromatic/olefinic protons. The ^13^C NMR and DEPT spectra display 18 carbons, of which twelve are olefinic carbons belonging to two phenyl groups. Besides, the residual signals include three methylene, one *sp*^3^ methine, and two quaternary carbons. The ^1^H–^1^H COSY spectrum (Fig. 2) shows correlations of H-4/H-5/H-6, H-7/H-8 and H-7′/H-8′. The HMBC correlations of H-7/C-2, C-6, C-9 (*δ*_C_ 177.7), H-8/C-1 (*δ*_C_ 142.5), in consideration of the chemical shift of C-1, indicate the presence of phenylpropanoid (part A). The HMBC correlations of H-7′/C-2′, C-6′, C-9′ (*δ*_C_ 177.4) and H-8′/C-1′ (*δ*_C_ 143.9) allow to establish the structure fragment of another phenylpropanoid (part B). In addition, the chemical shift of C-5′ and the HMBC correlations of H-4′, H-6′/C-8 (*δ*_C_ 55.3) indicate that parts A and B in **3** are linked via C-8–C-5′. Taken together, the data enable assignment of the planar structure of **3**. The lack of an optical rotation indicates that **3** is racemic. Separation by using chiral HPLC yielded two enantiomers, whose absolute configurations at stereogenic centers were assigned using computational methods. The ECD spectrum of (8*S*)-**3** is correlated well with the experimental spectrum of (+)-**3**, leading to the unambiguous assignment of the absolute configurations at the stereogenic centers in (+)-**3** as 8*S* (Fig. 3).

Plancyin D (**4**) has the molecular formula C_19_H_20_O_6_ (10 degrees of unsaturation), based on analysis of its HREIMS, ^13^C NMR, and DEPT spectra. The ^1^H and ^13^C NMR data of **4** resemble those of **3**, differing in that the additional existence of a methyl in **4**. The HMBC correlation of H-10′ (*δ*_H_ 3.63, s)/C-9′ (*δ*_C_ 175.2) indicates the connection position of the methyl group as shown. Thus, the planar structure of **4** was determined. For the structure like **3**, it is challenging to clarify the stereochemistry at C-8. Separation of **4** by chiral HPLC afforded (+)-**4** and (−)-**4**, whose absolute configurations were further determined by ECD calculations and comparison (Supplementary material) with the data of **3**, allowing (+)-**4** to be 8*S*.

Plancyin E (**5**) has the molecular formula C_17_H_18_O_4_ (9 degrees of unsaturation), based on analysis of its HREIMS and NMR data. Inspection of these NMR data disclosed that the structure of **5** extremely resemble that of **3**, differing in that a carboxyl group of C-8 is absent gaining support from the HMBC correlations of H-8/C-1, C-4′, C-6′ and the chemical shift of C-8 (*δ*_C_ 39.0). Therefore, the structure of **5** was constructed.

**Fig. 4:**
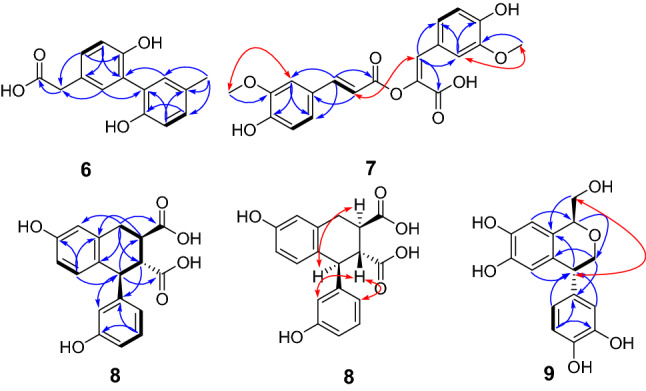
^1^H–^1^H COSY (black bond), Key HMBC (blue arrow), and ROESY (red arrow) correlations of **6**–**9**

Plancyin F (**6**) possesses a molecular formula C_15_H_14_O_4_ deduced from its negative HRESIMS and NMR data, indicating 9 degrees of unsaturation. The ^1^H NMR spectrum of **6** shows two ABX systems indicated by the signals at *δ*_H_ 7.15 (d, *J* = 1.8 Hz, H-2), and 6.86 (d, *J* = 8.1 Hz, H-5), 7.13 (dd, *J* = 8.1, 1.8 Hz, H-6) and *δ*_H_ 7.05 (d, *J* = 1.9 Hz, H-2′), 6.80 (d, *J* = 8.1 Hz, H-5′), and 7.01 (dd, *J* = 8.1, 1.9 Hz, H-6′), suggesting the presence of two 1,2,4-trisubstituted benzene rings. In addition, there exists a signal at *δ*_H_ 3.53 (s, 2H, H-7) and a methyl signal at *δ*_H_ 2.28 (s, 3H, H-7′). The ^13^C NMR and DEPT spectra display 15 carbons ascribed to one methyl, one methylene, six *sp*^2^ methine, seven quaternary carbons (one carbonyl, six *sp*^2^ including two oxygenated). The ^1^H–^1^H COSY spectrum (Fig. 4) gives cross peaks of H-5/H-6 and H-5′/H-6′, in conjunction with the HMBC correlations of H-7/C-2, C-6, C-8 (*δ*_C_ 177.3), and the chemical shift of C-4 (*δ*_C_ 154.0), revealing the west part of **6**. The HMBC correlations of H-7′/C-1′, C-2′, C-6′ and H-6′/C-4′ (*δ*_C_ 152.7) allow to establish the structure fragment of the east part. Two parts are connected via C-3–C-3′ aided by the HMBC correlations of H-2/C-3′ and H-2′/C-3. The structure of **6** was therefore identified.

Plancyin G (**7**) has the molecular formula C_20_H_18_O_8_ (12 degrees of unsaturation) deduced from its HRESIMS and NMR data. The ^1^H NMR spectrum of **7** shows the presence of two doublets at *δ*_H_ 7.59 and *δ*_H_ 6.37 both with *J* value of 15.9 Hz, indicating the *trans* relationship of H-7 and H-8, two ABX systems characteristic of signals at *δ*_H_ 7.31 (d, *J* = 1.7 Hz, H-2), 6.78 (d, *J* = 8.3 Hz, H-5), and 7.06 (dd, *J* = 8.3, 1.7 Hz, H-6) and *δ*_H_ 7.43 (d, *J* = 1.7 Hz, H-2′), 6.74 (d, *J* = 8.3 Hz, H-5′), and 7.08 (dd, *J* = 8.3, 1.7 Hz, H-6′), and two methyl signals respectively at *δ*_H_ 3.96 (s, H-10) and *δ*_H_ 3.69 (s, H-10′). The ^13^C NMR and DEPT spectra display 20 carbons ascribed to two methyl, nine *sp*^2^ methine, nine quaternary carbons (two carbonyl, seven *sp*^2^ including five oxygenated). The architecture of **7** was constructed mainly based on the HMBC spectrum (Fig. 4). In the HMBC spectrum, the correlations of H-7/C-2, C-6, C-9 (*δ*_C_ 170.8), H-7′/C-2′, C-6′, C-9′ (*δ*_C_ 167.7) indicate the presence of two phenylpropanoid parts, the correlations of H-5, H-10/C-3 (*δ*_C_ 150.5), H-5′, H-10′/C-3′ (*δ*_C_ 148.9) and ROESY correlations H-2/H-10, H-2′/H-10′ indicate the position of methoxyl, respectively. Further, a pivotal ROESY correlation of H-8/H-7′ in consideration of the chemical shift of C-8′ (*δ*_C_ 139.9) suggests the position of ester oxygen and the presence of *E-*configurated olefinic protons (H-7′ and H-8′), in accordance with the requirement of degrees of unsaturation. Taken together, these data enable assignment of the structure of **7**.

**Fig. 5:**
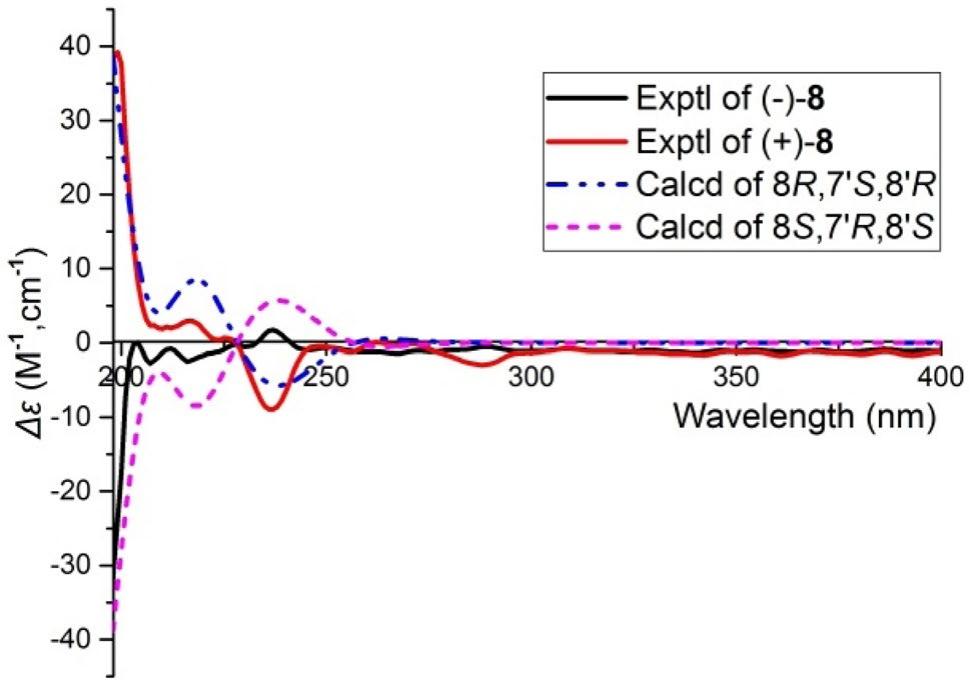
Comparison of calculated ECD spectra for (8*R*,7′*S*,8′*R*)-**8** and (8′*S*,7′*R*,8′*S*)-**8** with the experimental spectra of (+)**-8** and (−)**-8** in MeOH. *σ* = 0.3 eV; shift = − 7 nm

Plancyin H (**8**) possesses a molecular formula C_18_H_16_O_6_ (11 degrees of unsaturation) deduced from its HRESIMS and NMR data. The ^1^H NMR spectrum indicates the presence of seven aromatic/olefinic protons. The ^13^C NMR and DEPT spectra display 18 carbons, of which twelve are olefinic carbons belonging to two phenyl groups. Besides, the residual signals include one methylene, three *sp*^3^ methine, and two quaternary carbons (carbonyl). The HMBC correlations (Fig. 4) of H-7/C-2, C-6, C-9 (*δ*_C_ 178.3), H-8/C-1, and the ^1^H–^1^H COSY correlation of H-7/H-8 indicate the substructure of C-1–C-7–C-8–C-9 (part A). The structure fragment C-1′–C-7′–C-8′–C-9′ (part B) was established the HMBC correlations of H-7′/C-2′, C-6′, C-9′ (*δ*_C_ 178.4), H-8′/C-1′, and the ^1^H–^1^H COSY correlation of H-7′/H-8′. Further, the ^1^H–^1^H COSY correlation of H-8/H-8′ and the HMBC correlations of H-5/C-7′, H-7/C-8′, and H-7′/C-8 suggests two parts are connected via C-6–C-7′ and C-8–C-8′. The ^1^H–^1^H COSY correlations of H-4/H-5, H-5′/H-4′, H-6′, and HMBC correlations of H-5/C-1, C-3 (*δ*_C_ 156.6) and H-5′/C-1′, C-3′ (*δ*_C_ 158.4) indicate the replacement of benzene ring. Taken together, the planar structure of **8** was established as shown. There are three chiral centers in **8**, the ROESY correlations (Fig. 4) of H-8/H-7′ and H-8′/H-2′, H-6′ evidently imply the relative configuration of **8**. Compound **8** is racemic, chiral HPLC separation afforded (+)-**8** and (−)-**8**, whose absolute configurations was further determined as 8*R*,7′*S*,8′*R* for (+)-**8** and 8*S*,7′*R*,8′*S* for (−)-**8** by ECD comparison (Fig. 5).

**Fig. 6:**
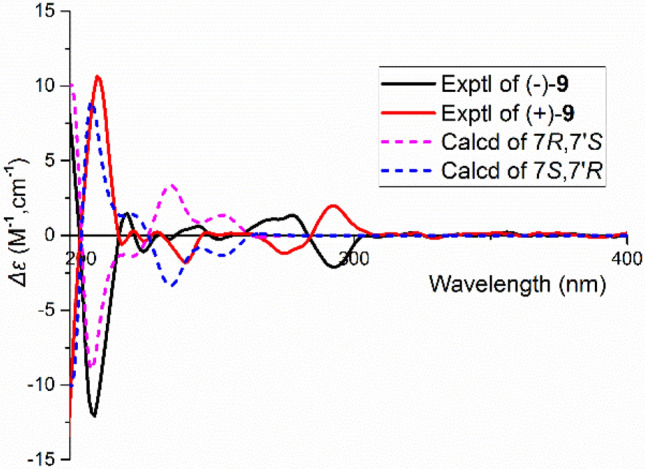
Comparison of calculated ECD spectra for (7*S*,7′*R*)-**9** and (7*R*,7′*S*)-**9** with the experimental spectra of (+)**-9** and (−)**-9** in MeOH. *σ* = 0.3 eV; shift = –3 nm

Compound **9** was determined to have the molecular formula C_16_H_16_O_6_ (9 degrees of unsaturation) on the basis of HRESIMS and NMR data. The ^1^H NMR spectrum shows an ABX system with signals resonated at *δ*_H_ 6.53 (d, *J* = 1.6 Hz, H-2′), 6.69 (d, *J* = 8.0 Hz, H-5′), and 6.50 (dd, *J* = 8.0, 1.6 Hz, H-6′). Two aromatic protons at *δ*_H_ 6.58 (s, H-2) and *δ*_H_ 6.31 (s, H-5) suggest the presence of a 1,2,4,5-tetrasubstituted benzene ring. The ^13^C NMR and DEPT spectra indicate 16 carbons ascribed to two oxygenated methylene, seven methine (5 *sp*^2^ and 2 *sp*^3^), and seven quaternary carbons (4 oxygenated). The ^1^H–^1^H COSY spectrum shows correlations of H-5′/H-6′; H-7/H-8, and H-7′/8′. The HMBC correlations of H-7′/C-1, C-1′, C-2′, C-6 and C-6′ indicate that the two phenyl groups are connected via C-7′. The HMBC correlations of H-8/C-1, H-2/C-7, H-7/C-8′, and H-8′/C-1′, C-6 suggest the structural motif of an isochroman. Taken together, the planar structure of **9** was determined as shown. There are two chiral centers in **9**, the ROESY spectrum (Fig. 4) of **9** displays the correlation of H-7′/H-8. We found that the NMR data of compound **9** are exactly same as those of periplanol A [20]. However, the absolute configuration of periplanol A has not been determined yet. To clarify the absolute configuration of **9**, chiral HPLC separation was first conducted due to it is racemic. The absolute configuration of each enantiomer of racemic **9** was assigned by using ECD calculations. The ECD spectrum (Fig. 6) of (7*R*,7′*S*)-**9** agrees well with the experimental one of (−)-**9**, leading to the unambiguous assignment of the absolute configurations at the stereogenic centers in (−)-**9** as 7*R*,7′*S* and (+)-**9** as 7*S*,7′*R* (Tables 2 and 3).

**Table 2:** ^1^H (600 MHz) and ^13^C NMR (150 MHz) data of **4**–**6** (*δ* in ppm, *J* in Hz, in methanol-*d*_4_)

No.	**4**		**5**		**6**	
	*δ*_H_	*δ*_C_	*δ*_H_	*δ*_C_	*δ*_H_	*δ*_C_
1		142.7		144.7		128.4
2	6.58, brs	116.9	6.60, brs	116.3	7.15, d, 1.8	133.6
3		158.2		158.4		127.7
4	6.55, dd, 8.1, 1.9	114.0	6.58, dd, 8.1, 1.9	113.7		154.0
5	7.00, t-like	130.1	7.04, t-like	130.2	6.86, d, 8.1	117.4
6	6.60, overlap	121.3	6.63, overlap	120.8	7.13, dd, 8.1, 1.8	130.5
7	3.23, dd, 13.6, 8.5	40.9	2.76, overlap	39.0	3.53, s	41.9
	2.84, dd, 13.6, 6.7					
8	3.66, overlap	55.8	2.76, overlap	39.0		177.3
9		178.3		177.4		
1′		143.4		143.6		130.6
2′	6.50, brs	114.8	6.47, brs	113.7	7.05, d, 1.9	133.0
3′		158.6		158.3		127.3
4′	6.62, brs	113.9	6.45, brs	114.2		152.7
5′		142.9		144.7	6.80, d, 8.1	117.3
6′	6.63, brs	120.5	6.51, brs	120.9	7.01, dd, 8.1, 1.9	130.2
7′	2.79, t, 7.6	31.9	2.78, t, 7.7	32.2	2.28, s	20.6
8′	2.56, t, 7.6	36.6	2.51, t, 7.7	37.2		
9′		175.2		177.4		
10′	3.63, s	52.1

**Table 3:** ^1^H (600 MHz) and ^13^C NMR (150 MHz) data of **7**–**9** (*δ* in ppm, *J* in Hz)

No.	**7**		**8**			**9**	
	*δ*_H_^a^	*δ*_C_^a^	*δ*_H_^a^	*δ*_H_^b^	*δ*_C_^a^	*δ*_H_^a^	*δ*_C_^a^
1		130.4			136.9		127.0
2	7.31, d, 1.7	112.6	6.57, overlap	7.03, overlap	115.1	6.58, s	111.9
3		150.5			156.6		145.0
4		149.4	6.47, dd, 8.5, 2.1	6.91, brd, 7.3	114.9		145.2
5	6.78, d, 8.3	114.7	6.52, d, 8.5	6.96, d, 7.8	131.5	6.31, s	116.8
6	7.06, dd, 8.3, 1.7	123.2			130.5		131.2
7	7.59, d, 15.9	146.0	3.07, overlap	3.42, overlap	34.0	4.76, dd, 5.2, 2.5	78.4
8	6.37, d, 15.9	117.8	3.03, overlap	3.42, overlap	45.7	3.86, overlap; 3.74, dd, 11.8, 5.2	66.3
9		170.8			178.3		
10	3.96, s	56.7					
1′		125.9			147.0		135.6
2′	7.43, d, 1.7	113.8	6.57, overlap	7.03, overlap	117.1	6.53, d, 1.6	117.0
3′		148.9			158.4		146.2
4′		149.6	6.65, dd, 8.1, 1.8	7.08, brd, 7.5	114.7		145.1
5′	6.74, d, 8.3	116.2	7.09, t-like	7.27, t-like	130.4	6.69, d, 8.0	116.2
6′	7.08, dd, 8.3, 1.7	126.3	6.62, brd, 7.6	7.34, brs	121.7	6.50, dd, 8.0, 1.6	121.5
7′	7.35, s	128.2	4.06, d, 10.3	4.73, d, 10.2	50.2	3.87, overlap	45.0
8′		139.9	2.97, overlap	3.80, t-like	53.6	4.10, dd, 11.0, 5.1; 3.64, dd, 11.0, 8.9	71.1
9′		167.7			178.4		
10′	3.69, s	56.0					

^a^In methanol-*d*_4_

^b^In pyridine-*d*_5_

The known compound (**10**) was identified as 5–5′ dehydrodi-3-(4-hydroxy-3-methoxyphenyl) propionic acid by comparing its NMR data with those in the literature [21].

Proteins or peptides are commonly considered to be the active substances in the insects. However, the chemical profiling and biological role of nonpeptidal small molecules in the insects remains largely unknown. In this study, ten lignan derivatives were characterized. It is evident that compounds **5**, **6**, and **9** are diverse norlignans and there exist four types of connection pattern for the ten lignans, adding new facets for the insect derived natural products.

### Biological Evaluation

**Fig. 7:**
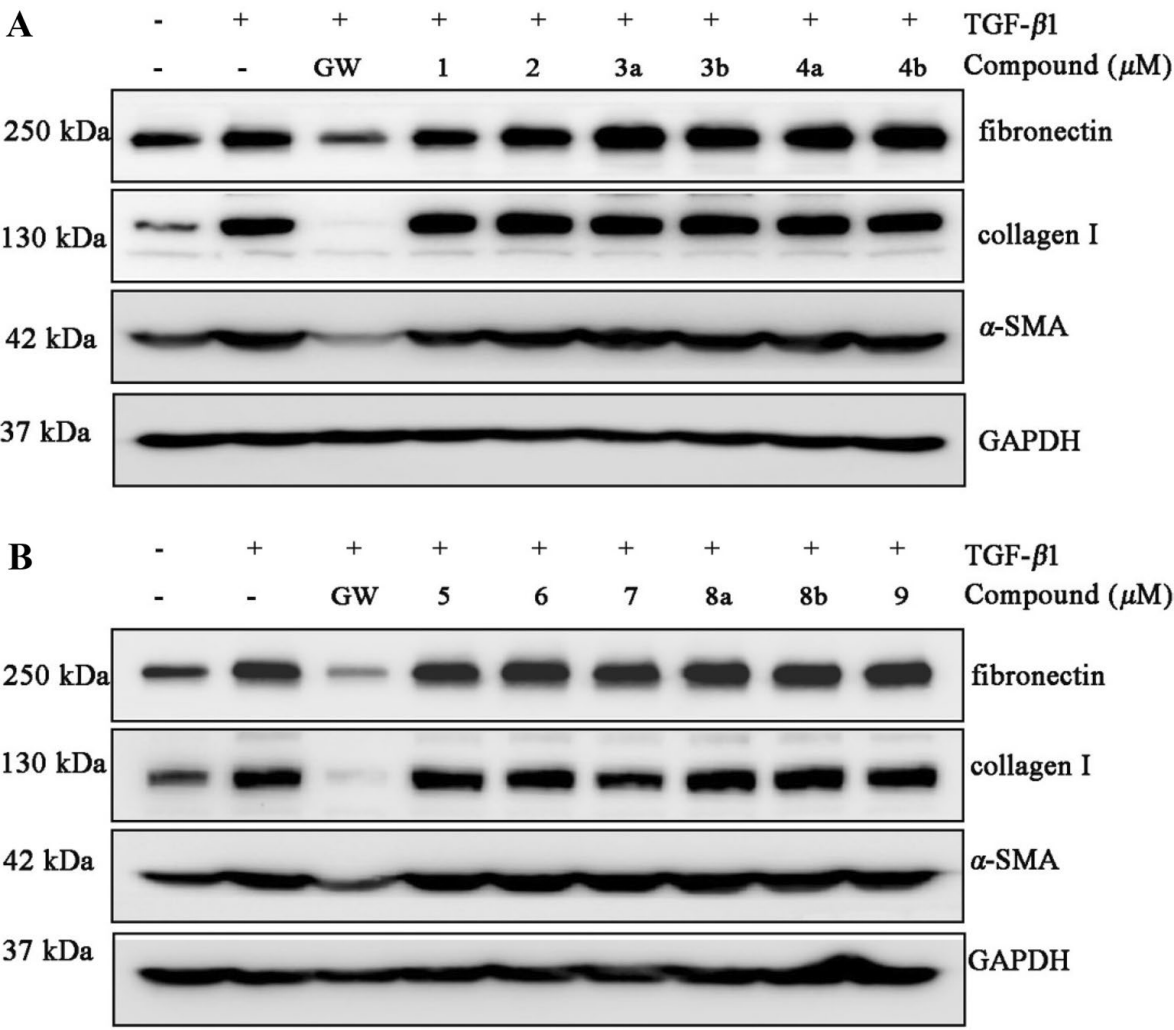
Western blot analysis showing that **1** and **7** protect against TGF-*β*1-mediated renal fibrosis. NRK-52e cells were incubated with TGF-*β*1 (10 ng/mL) for 48 h in the absence or presence of the compounds (40 μM). Cell lysates were immunoblotted with antibodies against fibronectin, collagen I, *α*-SMA, and GAPDH. GAPDH and GW were used as a normalizing control and a positive control, respectively. GW: GW788388 (Medchemexpree, HY-10326)

**Fig. 8:**
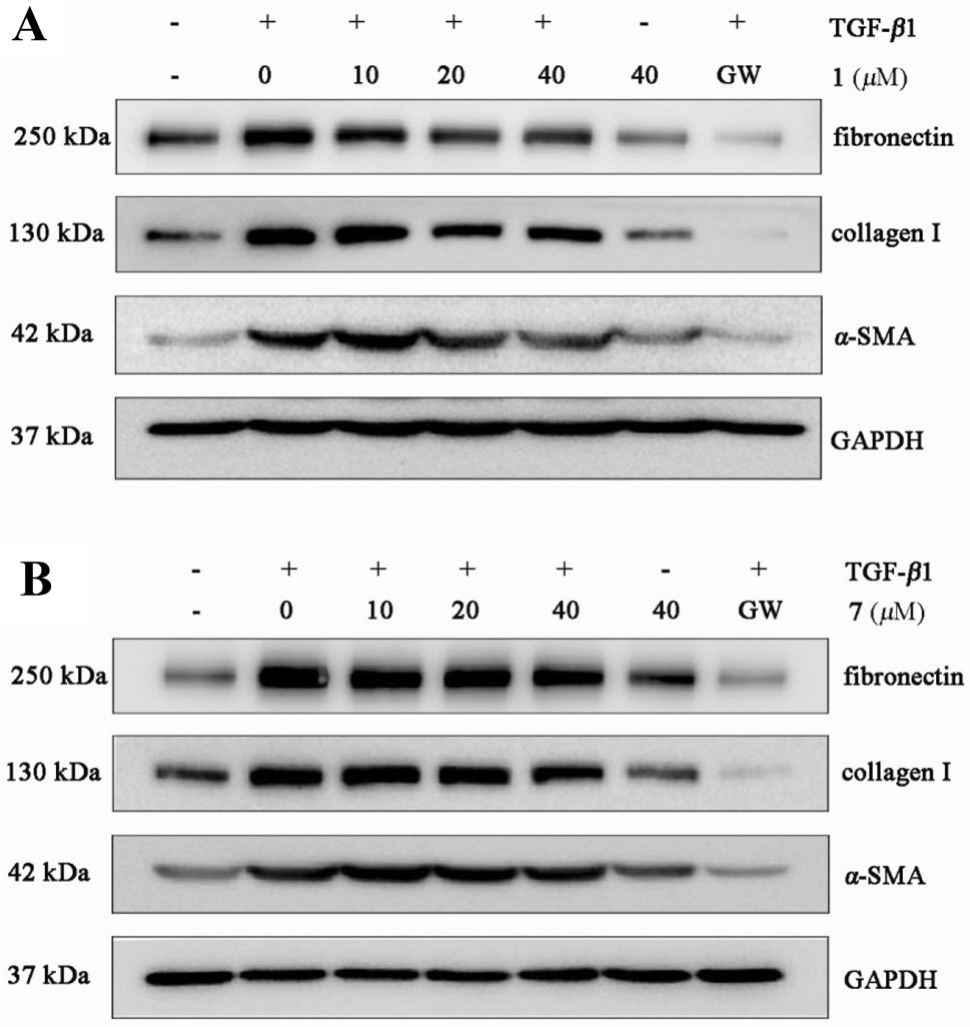
Western blot analysis showing that **1** and **7** block TGF-*β*1-mediated renal fibrosis. NRK-52e cells were preincubated with different concentrations of the compounds for 48 h before TGF-*β*1 (10 ng/mL) treatment. Cell lysates were immunoblotted with antibodies against fibronectin, collagen I, *α*-SMA, and GAPDH

**Fig. 9:**
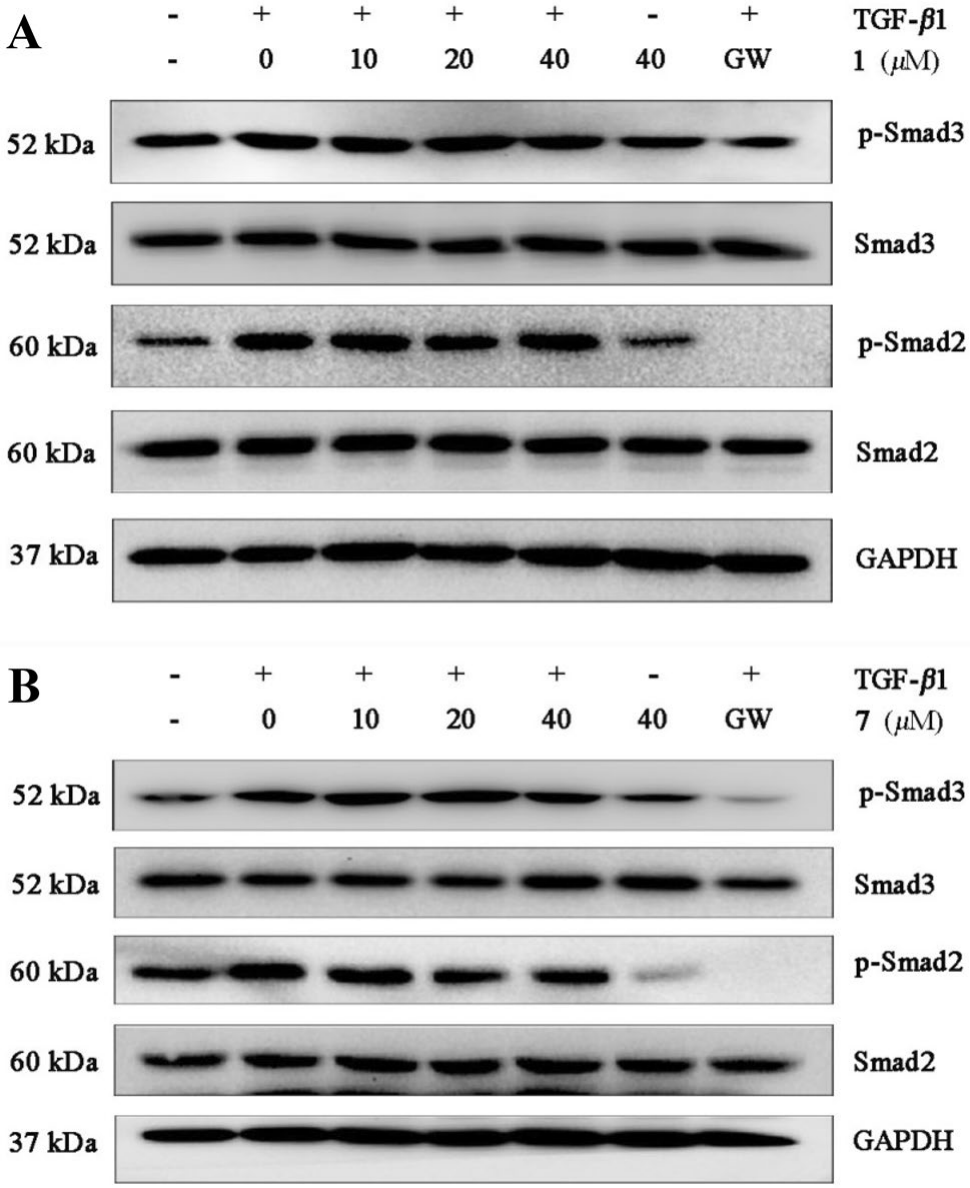
Western blot analysis showing that **1** and **7** inhibit TGF-*β*1–induced Smad2/3 phosphorylation in NRK-52e cells. NRK-52e cells were treated with TGF-*β*1 (10 ng/mL) for 1 h in the absence or presence of different concentrations of the compounds. Cell lysates were immunoblotted with antibodies against p-Smad3, Smad3, p-Smad2, Smad2, and GAPDH

To explore the mechanism underlying the antifibrotic effect of the compounds, we first examined whether the compounds affected TGF-*β*1–induced activation of the marker genes in NRK-52e cells (40 μM, data not shown). As presented in Fig. 7, compound **1** reduces three marker genes, especially for fibronectin and *α*-SMA in TGF-*β*1–induced NRK-52e cells (Fig. 7a). Compound **7** significantly inhibits three marker genes (Fig. 7b). Besides, Western bolt assay shows that compounds **1** and **7** decrease the protein expression with a dose-dependent manner in TGF-*β*1-induced NRK-52e cells (Fig. 8a, b). Next, we assessed the expression of Smad2/3 phosphorylation in NRK-52e cells. As presented in Fig. 9, compound **1** inhibits Smad3 phosphorylation, but does not affect TGF-*β*1–induced Smad2 phosphorylation (Fig. 9a). Compound **7** decreases both Smad3 and Smad2 phosphorylation level, and the effect for Smad2 appears to be more potent than Smad3 at 40 μM (Fig. 9b). Although more investigation are needed, our current study provides evidence that compounds **1** and **7** can play potential roles in the therapy of renal fibrosis by the disruption of Smad activation.

In addition, all the new compounds were evaluated for their inhibitory activities against human cancer cells (K562, A549, and Huh7), EV71, ROCK2, JAK3, DDR1, and coagulation. Compounds (+)-**3** and (−)-**3** show cytotoxicity against K562, A549, and Huh-7 cell lines with IC_50_ values ranging from 16.6 to 67.7 µM (Table 4). In contrast, compounds (+)-**3** and (−)-**3** also exhibit inhibitory activity against EV71 with IC_50_ values of 23.2 μM and 27.1 μM, respectively. It was found that compound **6** exhibits potent inhibitory activities toward these kinases with IC_50_ values of 9.17 μM for JAK3 and 27.1 μM for ROCK2. Compounds (+)-**9** and (−)-**9** exhibit inhibitory activities against JAK3 and DDR1 kinases (Table 5). In addition, partial compounds exhibit negligible inhibitory effect on coagulant (Tables 6, 7 and 8).

**Table 4:** Cytotoxicity against three different human cancer cell lines

Groups	IC_50_ (μM)		
	K562	A549	Huh7
(+)-**3**	48.1	> 70	> 70
(−)-**3**	16.6	43.1	67.7
Taxol	0.004	0.0027	0.0099

**Table 5:** Inhibitory effects of the isolates toward JAK3, ROCK2, DDR1, and EV71

Group	IC_50_ (μM)			
	JAK3	ROCK2	DDR1	EV71
(+)-**3**	NA^a^	NA^a^	NA^a^	23.2
(−)-**3**	NA^a^	NA^a^	NA^a^	27.1
**6**	9.17	27.1	NA^a^	NA^a^
(+)-**9**	9.35	NA^a^	23.9	NA^a^
(−)-**9**	4.59	NA^a^	22.9	NA^a^
Positive drug	Staurosporine 0.00044	Staurosporine 0.00381	Dasatinib 0.00744	Ribavirin 0.000384

^a^NA means no activity

**Table 6:** Inhibitory effects of the isolates on PT (n = 3)

Group	Detectable level (μM)	Plasma level (μM)	PT (s) *X* ± SD
Control	–	–	16.0 ± 0.12
Heparin	141.09	14.11	21.4 ± 0.50***
(+)-**4**	200.00	20.00	16.6 ± 0.26*
**7**	200.00	20.00	16.4 ± 0.06**

*P < 0.05, **P < 0.01, *** *P* < 0.001 versus control

**Table 7:** Inhibitory effects of the isolates on TT (n = 3)

Group	Detectable level (μM)	Plasma level (μM)	TT (s) *X* ± SD
Control	–	–	15.1 ± 0.15
Enoxaparin	35.60	3.56	20.4 ± 1.2*
(+)**-9**	200.00	20.00	16.1 ± 0.55*

*P < 0.05 versus control

**Table 8:** Inhibitory effects of the isolates on AA induced platelet aggregation in rabbits ($\overline{X}$ ± SD, n = 3)

Group		Inducer (AA)	Maximum aggregation rate (%)	Inhibition rate (%)
No.	Final level	Final level		
DMSO	1%	–	1.6 ± 1.5***	–
Control	1%	0.5 mM	70.6 ± 3.4	–
Aspirin	27.78 μM		12.0 ± 15.1***	82.5 ± 21.9***
**5**	20.00 μM		65.3 ± 1.5*	8.2 ± 4.7*
(+)-**9**	20.00 μM		65.0 ± 2.0*	8.5 ± 8.8*

*P < 0.05, *** *P* < 0.001 versus control

## Experimental Section

### General Experimental Procedures

Optical rotations were recorded on a Horiba SEPA-300 polarimeter. UV spectra were measured on a Shimadzu UV-2401PC spectrometer. CD spectra were determined on a Chirascan instrument. NMR spectra were recorded on a Bruker AV-600 MHz spectrometer, with TMS as an internal standard. ESIMS and HRESIMS were collected on an Agilent 1290 UPLC/6540 Q-TOF spectrometer. C-18 silica gel (40–60 μm; Daiso Co., Japan), MCI gel CHP 20P (75–150 μm, Mitsubishi Chemical Industries, Tokyo, Japan) and Sephadex LH-20 (Amersham Pharmacia, Uppsala, Sweden) were used for column chromatography. Semi-preparative HPLC was underwent on an Agilent 1200 liquid chromatograph with an YMC-Pack ODS-A column (250 × 10 mm, i.d., 5 μm, flow rate: 3 mL/min) and a Daicel Chiralpak (IC, 250 mm × 4.6 mm, i.d., 5 μm, flow rate: 1 mL/min).

### Insect Material

The specimen of *Polyphaga plancyi* was purchased from Henan Province, China, in November 2014, and identified by Prof. Da-Rong Yang at Kunming Institute of Zoology, Chinese Academy of Sciences. A voucher specimen (CHYX-0593) is deposited at the School of Pharmaceutical Sciences, Shenzhen University Health Science Center, People’s Republic of China.

### Extraction and Isolation

The whole bodies of *P. plancyi* (50 kg) were extracted under reflux with 70% EtOH (300 L, 4 h, 3 h, 3 h) to give a crude extract (6.32 kg), which was suspended in water followed by successive partition with petroleum ether and EtOAc to afford an EtOAc soluble extract. This extract (127 g) was divided into six parts (Frs. A–F) by using a MCI gel CHP 20P column eluted with gradient aqueous MeOH (10%–100%). Fr. B (8.1 g) was gel filtrated over Sephadex LH-20 (aqueous MeOH, 80%) to afford 3 parts (Frs. B1–B3). Compound **9** (2.6 mg, *t*_R_ = 19.2 min) was obtained from Fr. B3 (0.40 g) by semi-preparative HPLC (MeCN/H_2_O, 13%, flow rate: 3 mL/min). Fr. C (12.0 g) was gel filtrated over Sephadex LH-20 (aqueous MeOH, 80%) to afford 5 parts (Frs. C1–C5). Fr. C2 (5.51 g) was submitted to a RP-18 column eluted with gradient aqueous MeOH (15–50%) to yield 8 subfractions (Frs. C2.1–C2.8). Fr. C3 (2.45 g) was submitted to a RP-18 column eluted with gradient aqueous MeOH (15–50%) followed by semi-preparative HPLC (MeCN/H_2_O, 29%, flow rate: 3 mL/min) to produce **4** (1.3 mg, *t*_R_ = 28.0 min). Fr. C4 (1.71 g) was gel filtrated over Sephadex LH-20 (MeOH) followed by semi-preparative HPLC (MeCN/H_2_O, 21%, flow rate: 3 mL/min) to produce **8** (2.6 mg, *t*_R_ = 27.2 min). Fr. D (8.7 g) was gel filtrated over Sephadex LH-20 (aqueous MeOH, 80%) to afford 6 parts (Frs. D1–D6). Further, Fr. D5 (1.23 g) was gel filtrated over Sephadex LH-20 (MeOH) followed by semi-preparative HPLC (MeCN/H_2_O, 29%, flow rate: 3 mL/min) to produce **3** (22.5 mg, *t*_R_ = 11.6 min), **10** (2.5 mg, *t*_R_ = 12.3 min), **7** (2.1 mg, *t*_R_ = 19.2 min), **6** (2.8 mg, *t*_R_ = 28.4 min), and **5** (5.5 mg, *t*_R_ = 34.0 min). Fr. D6 (1.01 g) was gel filtrated over Sephadex LH-20 (MeOH) followed by semi-preparative HPLC (MeCN/H_2_O, 29%, flow rate: 3 mL/min) to produce **1** (12.5 mg, *t*_R_ = 12.2 min) and **2** (3.8 mg, *t*_R_ = 34.5 min). Notably, compounds **3**, **4**, **8**, and **9** were isolated as racemates, which were subjected to chiral HPLC to yield (+)-**3** (10.1 mg, *t*_R_ = 8.3 min) and (−)-**3** (10.5 mg, *t*_R_ = 11.6 min) (n-hexane/ethanol, 85:15, flow rate: 1 mL/min), (+)-**4** (0.7 mg, *t*_R_ = 10.4 min) and (−)-**4** (0.5 mg, *t*_R_ = 15.8 min) (n-hexane/ethanol, 80:20, flow rate: 1 mL/min), (+)-**8** (1.4 mg, *t*_R_ = 12.5 min) and (−)-**8** (1.1 mg, *t*_R_ = 14.6 min) (n-hexane/ethanol, 90:10, flow rate: 1 mL/min), (+)-**9** (1.2 mg, *t*_R_ = 8.9 min) and (−)-**9** (1.1 mg, *t*_R_ = 14.3 min) (n-hexane/ethanol, 77:23, flow rate: 1 mL/min), respectively.

### Compound Characterization

Plancyin A (**1**): Yellowish solid; UV (MeOH) *λ*_max_ (log *ε*) 288 (3.82), 255 (4.02), 211 (4.45) nm; ESIMS *m/z* 329 [M−H]^−^, HRESIMS *m/z* 329.1040 [M−H]^−^ (calcd for C_18_H_17_O_6_, 329.1031). ^1^H and ^13^C NMR data, see Table 1.

Plancyin B (**2**): Yellowish solid; UV (MeOH) *λ*_max_ (log *ε*) 291 (3.76), 256 (4.00), 214 (4.54) nm; ESIMS *m/z* 343 [M−H]^−^, HRESIMS *m/z* 343.1193 [M−H]^−^ (calcd for C_19_H_19_O_6_, 343.1187). ^1^H and ^13^C NMR data, see Table 1.

Plancyin C (**3**): Yellowish solid; {[*α*]_D_^25^ + 49.5 (*c* 0.17, MeOH); CD (MeOH) Δ*ε*_195_ − 27.87, Δ*ε*_208_ + 15.93; (+)-**3**}; {[*α*]_D_^25^ − 58.0 (*c* 0.16, MeOH); CD (MeOH) Δ*ε*_195_ + 18.55, Δ*ε*_208_ − 11.62; (−)-**3**}; UV (MeOH) *λ*_max_ (log *ε*) 278 (3.77), 205 (4.80) nm; ESIMS *m/z* 329 [M−H]^−^, HRESIMS *m/z* 329.1036 [M−H]^−^ (calcd for C_18_H_17_O_6_, 329.1031). ^1^H and ^13^C NMR data, see Table 1.

Plancyin D (**4**): Yellowish solid; {[*α*]_D_^25^ + 86.7 (*c* 0.07, MeOH); CD (MeOH) Δ*ε*_197_ − 39.04, Δ*ε*_209_ + 15.41; (+)-**4**}; {[*α*]_D_^25^ − 86.5 (*c* 0.05, MeOH); CD (MeOH) Δ*ε*_195_ + 33.86, Δ*ε*_208_ − 17.87; (−)-**4**}; UV (MeOH) *λ*_max_ (log *ε*) 277 (3.53), 205 (4.60) nm; ESIMS *m/z* 343 [M−H]^−^, HRESIMS *m/z* 343.1178 [M−H]^−^ (calcd for C_19_H_19_O_6_, 343.1187). ^1^H and ^13^C NMR data, see Table 2.

Plancyin E (**5**): Yellowish solid; UV (MeOH) *λ*_max_ (log *ε*) 277 (3.60), 217 (4.24), 205 (4.60) nm; ESIMS *m/z* 285 [M−H]^−^, HRESIMS *m/z* 285.1133 [M−H]^−^ (calcd for C_17_H_17_O_4_, 285.1132). ^1^H and ^13^C NMR data, see Table 2.

Plancyin F (**6**): Yellowish solid; UV (MeOH) *λ*_max_ (log *ε*) 291 (3.72), 208 (4.44) nm; ESIMS *m/z* 257 [M−H]^−^, HRESIMS *m/z* 257.0816 [M−H]^−^ (calcd for C_15_H_13_O_4_, 257.0819). ^1^H and ^13^C NMR data, see Table 2.

Plancyin G (**7**): Yellowish solid; UV (MeOH) *λ*_max_ (log *ε*) 323 (4.39), 204 (4.39) nm; ESIMS *m/z* 385 [M−H]^−^, HRESIMS *m/z* 385.0917 [M−H]^−^ (calcd for C_20_H_17_O_8_, 385.0929). ^1^H and ^13^C NMR data, see Table 3.

Plancyin H (**8**): Yellowish solid; {[*α*]_D_^25^ + 34.9 (*c* 0.11, MeOH); CD (MeOH) Δ*ε*_198_ + 29.58, Δ*ε*_230_ − 5.48; (+)-**8**}; {[*α*]_D_^25^ − 29.6 (*c* 0.14, MeOH); CD (MeOH) Δ*ε*_198_ − 37.08, Δ*ε*_237_ + 2.03; (−)-**8**}; UV (MeOH) *λ*_max_ (log *ε*) 280 (3.53), 221 (4.15), 205 (4.52) nm; ESIMS *m/z* 327 [M−H]^−^, HRESIMS *m/z* 327.0873 [M−H]^−^ (calcd for C_18_H_15_O_6_, 327.0874). ^1^H and ^13^C NMR data, see Table 3.

Periplanol A (**9**): Yellowish solid; {[*α*]_D_^25^ + 36.1 (*c* 0.04, MeOH); CD (MeOH) Δε_197_ − 6.41, Δε_206_ + 11.24, Δ*ε*_292_ + 2.06; (+)-**9}**; {[*α*]_D_^25^ − 12.8 (*c* 0.41, MeOH); CD (MeOH) Δε_196_ + 7.91, Δε_204_ − 12.17, Δ*ε*_294_ − 2.08; (−)-**9}**; UV (MeOH) *λ*_max_ (log *ε*) 507 (2.58), 474 (2.59), 290 (3.65), 206 (4.36); ESIMS *m/z* 303 [M−H]^−^, HRESIMS *m/z* 303.0863 [M−H]^−^ (calcd for C_16_H_15_O_6_, 303.0874). ^1^H and ^13^C NMR data, see Table 3.

### Biological Evaluation for Kidney Fibrosis

#### Cell Culture and Cytotoxicity Assay

NRK-52e cell lines were maintained in Dulbecco’s modified Eagle’s medium (DMEM) supplemented with 10% fetal bovine serum and 100 U/mL penicillin–streptomycin in 37 °C with atmosphere of 5% CO_2_. The growing cells were seeded at 1 × 10^4^ cells per well in 96-well culture plates for 24 h. Cells were exposed to the compounds with different concentrations (2.5, 5, 10, 20, 40 μM) for 48 h. The equal volume of DMSO was used as the solvent control. CCK-8 solution (10% v/v) was added and incubated for another 2 h. Light absorbance of the solution was measured at 450 nm (Epoch 2; BioTek Instruments, Inc.).

#### Western Blot Assay

Protein expression was analyzed by Western blot analysis as described previously [12]. The concentration of all the compounds was 40 μM for primary screening and 10, 20, 40 μM for dose-dependent experiments. The primary antibodies as follows: Anti-Fibronectin antibody [IST-9] (#ab6328; Abcam), COL1A1 Antibody (#84336; Cell Signaling Technology), *α*-Smooth Muscle Actin (D4K9N) XP® Rabbit mAb (#19245; Cell Signaling Technology), Smad3 (C67H9) Rabbit mAb (#9523, Cell Signaling Technology), Phospho-Smad3 (Ser423/425) (C25A9) Rabbit mAb (#9520, Cell Signaling Technology), Smad2 (D43B4) XP® Rabbit mAb (#5339, Cell Signaling Technology), Phospho-Smad2 (Ser465/467) (138D4) Rabbit mAb (#3108, Cell Signaling Technology), GAPDH (D16H11) XP® Rabbit mAb (#5174, Cell Signaling Technology). Cell pellets were collected and resuspended in RIPA lysis buffer (containing 0.1 mM PMSF). GW: GW788388 (Medchemexpree, HY-10326) [13].

### Biological Evaluation for Human Cancer Cells (K562, A549, and Huh7), ROCK2, JAK3, and DDR1

Compounds **1**–**9** were evaluated for their inhibitory effects against human cancer cells (K562, A549, and Huh7) [14], ROCK2 [15], JAK3 [16], and DDR1 [4] as previously described methods.

### Biological Evaluation for EV71

Compounds **1**–**9** were evaluated for their EV71 inhibitory activities in vitro. VERO cells were plated into 384-well plate at a suitable density and allowed to adhere prior to addition of varying concentrations of drugs. Then the cells were covered with enteroviruses EV71 which was diluted at fresh culture medium. After that, the cells were incubated at 37 °C for a further 72 h. Then discard the old medium, add fresh medium containing CCK-8, and incubated in 37 °C for 2 h. The A450 was then measured with an Envision Plate Reader (PerkinElmer).

### Anticoagulant Assay

The prothrombin time (PT) and thrombin time (TT) was determined with a coagulometer (TECO MC-4000, Germany). All the reagents were purchased from TECO GmbH (Germany). PT reagent, TT reagent, and normal human plasma was reconstituted in 4 mL (or 1 mL for plasma) of distilled water, according to the instructions of the manufacturer. The compounds were dissolved to 2 mM in DMSO at various concentrations. Then use 20 mM Tris–HCl pH 7.4 (including 5% Tween 80) diluted to 200 µM to be measured. For the PT and TT assays, 5 μL samples (or 10 μL for TT) were mixed with 45 μL (or 90 μL for TT) of normal human plasma and incubated for 2 min at 37 °C; 100 μL of PT (or 50 μL of TT) reagent was then added and the clotting time was recorded.

### Platelet Aggregation Assay

Turbidometric measurements of platelet aggregation inhibition were performed in a Chronolog Model 700 Aggregometer (Chronolog Corporation, Havertown, PA, USA) according to Born’s method [17, 18]. The present study was approved by the Research Ethics Committee of Kunming Institute of Botany, Chinese Academy of Sciences. The blood from the rabbits by ear central arter puncture, were anticoagulated with 3.8% sodium citrate (9:1, v/v). Platelet-rich plasma (PRP) and platelet-poor plasma (PPP) were prepared shortly after blood collection by spinning the sample at 180 g for 10 min at 22 °C. The PRP was carefully removed and the remaining blood centrifuged at 2400×*g* for 10 min to obtain PPP. The centrifuge temperature was maintained at 22 °C. Platelet counts were adjusted by the addition of PPP to the PRP to achieve a count of 250 × 10^9^/L. Platelet aggregation studies were completed within 3 h of preparation of PRP. Immediately after preparation of PRP, 250 μL was transferred into each of the test tubes, with 250 μL PPP set as a control. Before addition of inducers, the compounds were incubated with PRP at 37 °C for 5 min. Final concentration of agonist was: arachidonic acid (AA) 0.5 mM as positive reference. Percentage inhibition by the compounds was calculated according to the formula:

$${\text{Inhibition}}\;{\text{rate}}\;\left( \% \right) = \frac{(A - B)}{A} \times 100\%$$

where *A*: maximum change of turbidity in DMSO added, *B*: maximum change of turbidity in sample added.

#### Computational Methods

Molecular Merck force field (MMFF) and DFT/TDDFT calculations were performed with Spartan'14 software package (Wavefunction Inc., Irvine, CA, USA) and Gaussian 09 program package [19], the conformational search generated low-energy conformers within a 10 kcal/mol energy was finished by software Conflex 7. Geometry optimizations of compounds **3**, **8**, and** 9** were carried out at the DFT/B3LYP/6-311G (d, p) level. The calculated ECD spectra were determined by using Gaussian 09 software employing the TDDFT-B3LYP functional and the 6-311G (d, p) basis sets. ECD calculations further were conducted at the B3LYP SCRF (PCM)/6-311G (d,p) level in MeOH.

## Electronic supplementary material

Below is the link to the electronic supplementary material.

Supplementary file1 (DOCX 5910 kb)

## References

[1] Ratcliffe NA, Mello CB, Garcia ES, Butt TM, Azambuja P (2011). Insect Biochem. Mol. Biol..

[2] Dossy AD (2010). Nat. Prod. Rep..

[3] Chinese Pharmacopeia Committee, Pharmacopeia of the People’s Republic of China **1**, 15 (2015)

[4] Zhu HJ, Yan YM, Tu ZC, Luo JF, Liang R, Yang TH, Cheng YX, Wang SM (2016). Fitoterapia.

[5] Yan YM, Ai J, Shi YN, Zuo ZL, Hou B, Luo J, Cheng YX (2014). Org. Lett..

[6] Zhao J, Zhu HJ, Zhou XJ, Yang TH, Wang YY, Su J, Li Y, Cheng YX (2010). J. Nat. Prod..

[7] Yan YM, Dai HQ, Du YH, Schneider B, Guo H, Li DP, Zhang LX, Fu H, Dong XP, Cheng YX (2012). Bioorg. Med. Chem. Lett..

[8] Shi YN, Tu ZC, Wang XL, Yan YM, Fang P, Zuo ZL, Hou B, Yang TH, Cheng YX (2014). Bioorg. Med. Chem. Lett..

[9] Yan YM, Li LJ, Qin XC, Lu Q, Tu ZC, Cheng YX (2015). Bioorg. Med. Chem. Lett..

[10] Yan YM, Zhu HJ, Zhou FJ, Tu ZC, Cheng YX (2019). Tetrahedron.

[11] Li J, Li YP, Qin FY, Yan YM, Zhang HX, Cheng YX (2020). Fitoterapia.

[12] Luo CW, Zhou S, Zhou ZM, Liu YH, Yang L, Liu JF, Zhang YF, Li HY, Liu YH, Hou FF, Zhou LL (2018). J. Am. Soc. Nephrol..

[13] Gellibert F, de Gouville AC, Woolven J, Mathews N, Nguyen VL, Bertho-Ruault C, Patikis A, Grygielko ET, Laping NJ, Huet S (2006). J. Med. Chem..

[14] Wang JF, Wang Z, Ju ZR, Wan JT, Liao SR, Lin XP, Zhang TY, Zhou XF, Chen H, Tu ZC, Liu YH (2015). Planta Med..

[15] Tang JJ, Zhang L, Jiang LP, Di L, Yan YM, Tu ZC, Yang CP, Zuo ZL, Hou B, Xia HL, Chen YB, Cheng YX (2014). Tetrahedron.

[16] Tang JJ, Fang P, Xia HL, Tu ZC, Hou BY, Yan YM, Di L, Zhang L, Cheng YX (2015). Food Res. Int..

[17] Born GVR (1962). Nature.

[18] Born GVR, Cross MJ (1963). J. Physiol..

[19] Frisch MJ, Trucks GW, Schlegel HB, Scuseria GE, Robb MA, Cheeseman JR, Scalmani G, Barone V, Mennucci B, Petersson GA, Nakatsuji H, Caricato M, Li X, Hratchian HP, Izmaylov AF, Bloino J, Zheng G, Sonnenberg JL, Hada M, Ehara M, Toyota K, Fukuda R, Hasegawa J, Ishida M, Nakajima T, Honda Y, Kitao O, Nakai H, Vreven T, Montgomery JA, Peralta JE, Ogliaro F, Bearpark M, Heyd JJ, Brothers E, Kudin KN, Staroverov VN, Kobayashi R, Normand J, Raghavachari K, Rendell A, Burant JC, Iyengar SS, Tomasi J, Cossi M, Rega N, Millam JM, Klene M, Knox JE, Cross JB, Bakken V, Adamo C, Jaramillo J, Gomperts R, Stratmann RE, Yazyev O, Austin AJ, Cammi R, Pomelli C, Ochterski JW, Martin RL, Morokuma K, Zakrzewski VG, Voth GA, Salvador P, Dannenberg JJ, Dapprich S, Daniels AD, Farkas O, Foresman JB, Ortiz JV, Cioslowski J, Fox DJ (2009). Gaussian 09, Revision A.02.

[20] Yan YM, Zhu HJ, Xiang B, Qi JJ, Cheng YX (2017). Nat. Prod. Commun..

[21] Russell WR, Scobbie L, Chesson A (2005). Bioorg. Med. Chem..

