# Supplementary material for: Neolignans and Norlignans from Insect Medicine Polyphaga plancyi and Their Biological Activities
Source: Nat Prod Bioprospect. 2020 Sep 2;11(1):51–62. doi: 10.1007/s13659-020-00262-0 (PMC7933325; doi:10.1007/s13659-020-00262-0)
Supplement: Supplementary file 1 — Supplementary file1 (DOCX 5910 kb) [file 13659_2020_262_MOESM1_ESM.docx]

**Supplementary material**

Neolignans and norlignans from insect medicine *Polyphaga plancyi* and their biological activities**

Hong-Jie Zhu^b,1^, Te Xu^a,1^, Yong-Ming Yan^a^, Zheng-chao Tu^c,d^, Yong-Xian Cheng^a^*

^a^School of Pharmaceutical Sciences, Shenzhen University Health Science Center, Shenzhen 518060, People’s Republic of China

^b^State Key Laboratory of Phytochemistry and Plant Resources in West China, Kunming Institute of Botany, Chinese Academy of Sciences, Kunming 650201, People’s Republic of China

^c^International Cooperative Laboratory of Traditional Chinese Medicine Modernization and Innovative Drug Development of Chinese Ministry of Education (MOE), College of Pharmacy, Jinan University, Guangzhou 510632, People’s Republic of China

^d^Drug Discovery Pipeline & Guangdong Provincial Key Laboratory of Biocomputing, Guangzhou Institutes of Biomedicine and Health, Guangzhou 510530, People’s Republic of China

^1^ These authors contributed equally to this work.

* Corresponding author

Email address: yxcheng@szu.edu.cn.

** In honor of Professor Jun Zhou.

**Content**

Figure S1. ^1^H NMR spectrum of **1** in methanol-*d*_4_

Figure S2. ^13^C NMR and DEPT spectra of **1** in methanol-*d*_4_

Figure S3. ^1^H-^1^H COSY spectrum of **1** in methanol-*d*_4_

Figure S4. HSQC spectrum of **1** in methanol-*d*_4_

Figure S5. HMBC spectrum of **1** in methanol-*d*_4_

Figure S6. HRESIMS of **1**

Figure S7. ^1^H NMR spectrum of **2** in methanol-*d*_4_

Figure S8. ^13^C NMR and DEPT spectra of **2** in methanol-*d*_4_

Figure S9. ^1^H-^1^H COSY spectrum of **2** in methanol-*d*_4_

Figure S10. HSQC spectrum of **2** in methanol-*d*_4_

Figure S11. HMBC spectrum of **2** in methanol-*d*_4_

Figure S12. HRESIMS of **2**

Figure S13. ^1^H NMR spectrum of **3** in Methanol-*d*_4_

Figure S14. ^13^C NMR and DEPT spectra of **3** in methanol-*d*_4_

Figure S15. ^1^H-^1^H COSY spectrum of **3** in methanol-*d*_4_

Figure S16. HSQC spectrum of **3** in methanol-*d*_4_

Figure S17. HMBC spectrum of **3** in methanol-*d*_4_

Figure S18. HRESIMS of **3**

Figure S19. ECD comparison between **3** and **4**

Figure S20. ^1^H NMR spectrum of **4** in methanol-d4

Figure S21. ^13^C NMR and DEPT spectra of **4** in methanol-*d*_4_

Figure S22. ^1^H-^1^H COSY spectrum of **4** in methanol-*d*_4_

Figure S23. HSQC spectrum of **4** in methanol-*d*_4_

Figure S24. HMBC spectrum of **4** in methanol-*d*_4_

Figure S25. HRESIMS of **4**

Figure S26. ^1^H NMR spectrum of **5** in methanol-*d*_4_

Figure S27. ^13^C NMR and DEPT spectra of **5** in methanol-*d*_4_

Figure S28. ^1^H-^1^H COSY spectrum of **5** in methanol-*d*_4_

Figure S29. HSQC spectrum of **5** in methanol-*d*_4_

Figure S30. HMBC spectrum of **5** in methanol-*d*_4_

Figure S31. HRESIMS of **5**

Figure S32. ^1^H NMR spectrum of **6** in methanol-*d*_4_

Figure S33. ^13^C NMR and DEPT spectra of **6** in methanol-*d*_4_

Figure S34. ^1^H-^1^H COSY spectrum of **6** in methanol-*d*_4_

Figure S35. HSQC spectrum of **6** in methanol-*d*_4_

Figure S36. HMBC spectrum of **6** in methanol-*d*_4_

Figure S37. HRESIMS of **6**

Figure S38. ^1^H NMR spectrum of **7** in methanol-*d*_4_

Figure S39. ^13^C NMR and DEPT spectra of **7** in methanol-*d*_4_

Figure S40. ^1^H-^1^H COSY spectrum of **7** in methanol-*d*_4_

Figure S41. HSQC spectrum of **7** in methanol-*d*_4_

Figure S42. HMBC spectrum of **7** in methanol-*d*_4_

Figure S43. ROESY spectrum of **7** in methanol-*d*_4_

Figure S44. HRESIMS of **7**

Figure S45. ^1^H NMR spectrum of **8** in methanol-*d*_4_

Figure S46. ^13^C NMR and DEPT spectra of **8** in methanol-*d*_4_

Figure S47. ^1^H-^1^H COSY spectrum of **8** in methanol-*d*_4_

Figure S48. HSQC spectrum of **8** in methanol-*d*_4_

Figure S49. HMBC spectrum of **8** in methanol-*d*_4_

Figure S50. ^1^H NMR spectrum of **8** in pyridine-*d*_5_

Figure S51. ^13^C NMR spectrum of **8** in pyridine-*d*_5_

Figure S52. ^1^H-^1^H COSY spectrum of **8** in pyridine-*d*_5_

Figure S53. HSQC spectrum of **8** in pyridine-*d*_5_

Figure S54. HMBC spectrum of **8** in pyridine-*d*_5_

Figure S55. ROESY spectrum of **8** in pyridine-*d*_5_

Figure S56. HRESIMS of **8**

Figure S57. ^1^H NMR spectrum of **9** in methanol-*d*_4_

Figure S58. ^13^C NMR and DEPT spectra of **9** in methanol-*d*_4_

Figure S59. ^1^H-^1^H COSY spectrum of **9** in methanol-*d*_4_

Figure S60. HSQC spectrum of **9** in methanol-*d*_4_

Figure S61. HMBC spectrum of **9** in methanol-*d*_4_

Figure S62. ROESY spectrum of **9** in methanol-*d*_4_

Figure S63. HRESIMS of **9**


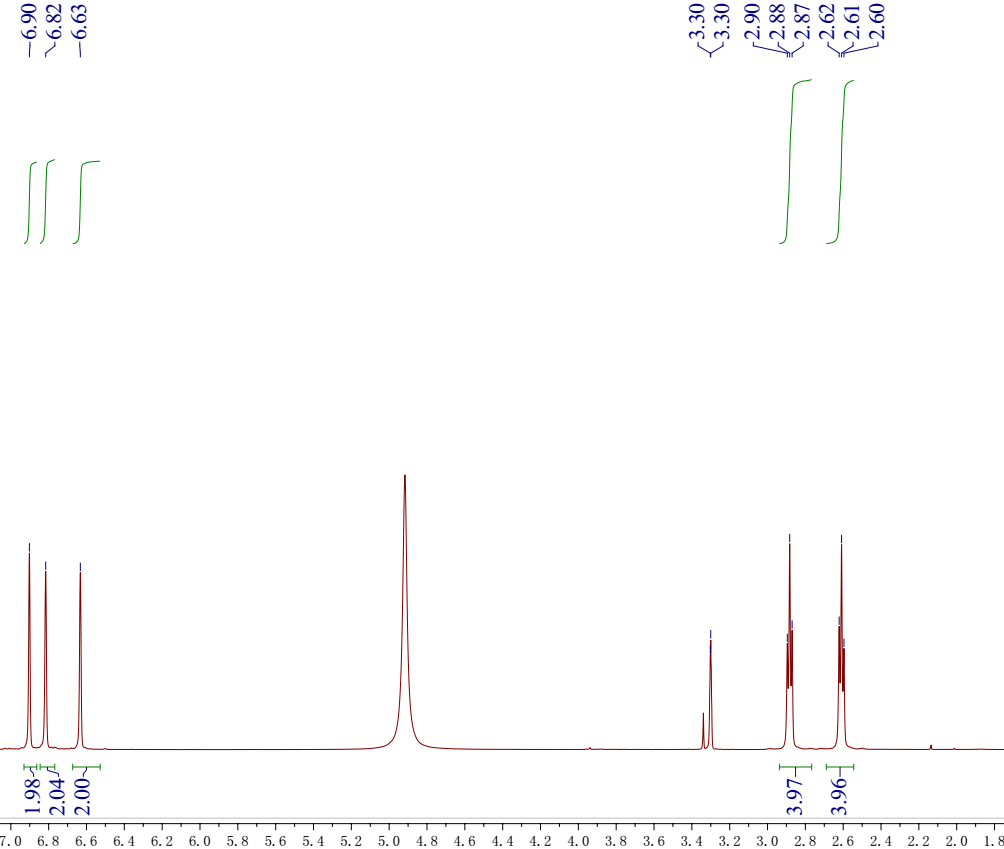


Figure S1. ^1^H NMR spectrum of **1** in methanol-*d*_4_


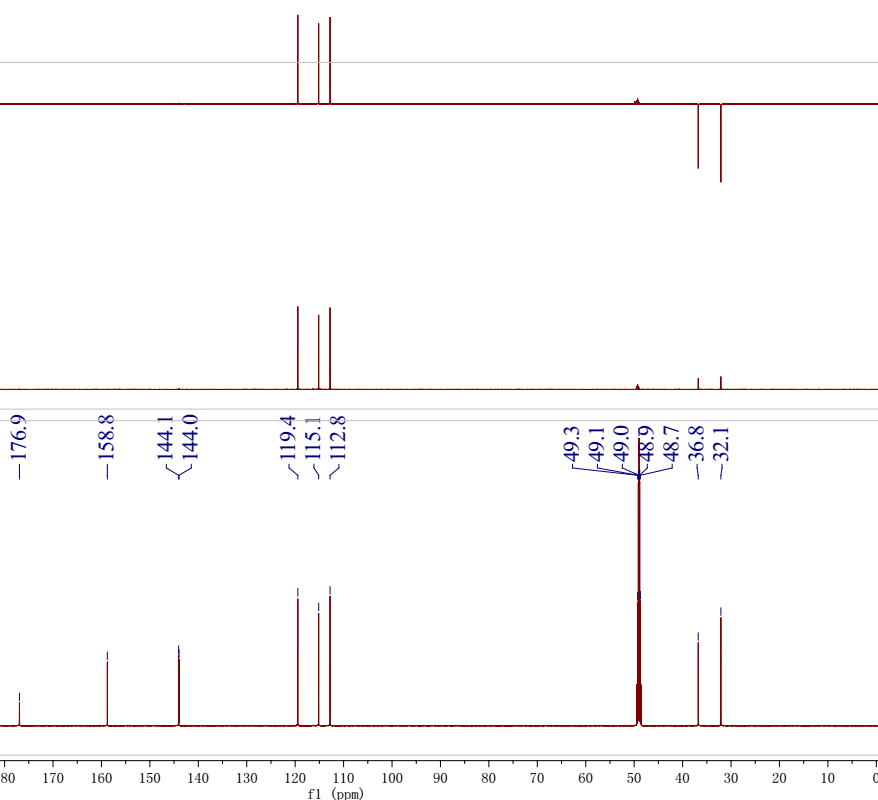


Figure S2. ^13^C NMR and DEPT spectra of **1** in methanol-*d*_4_


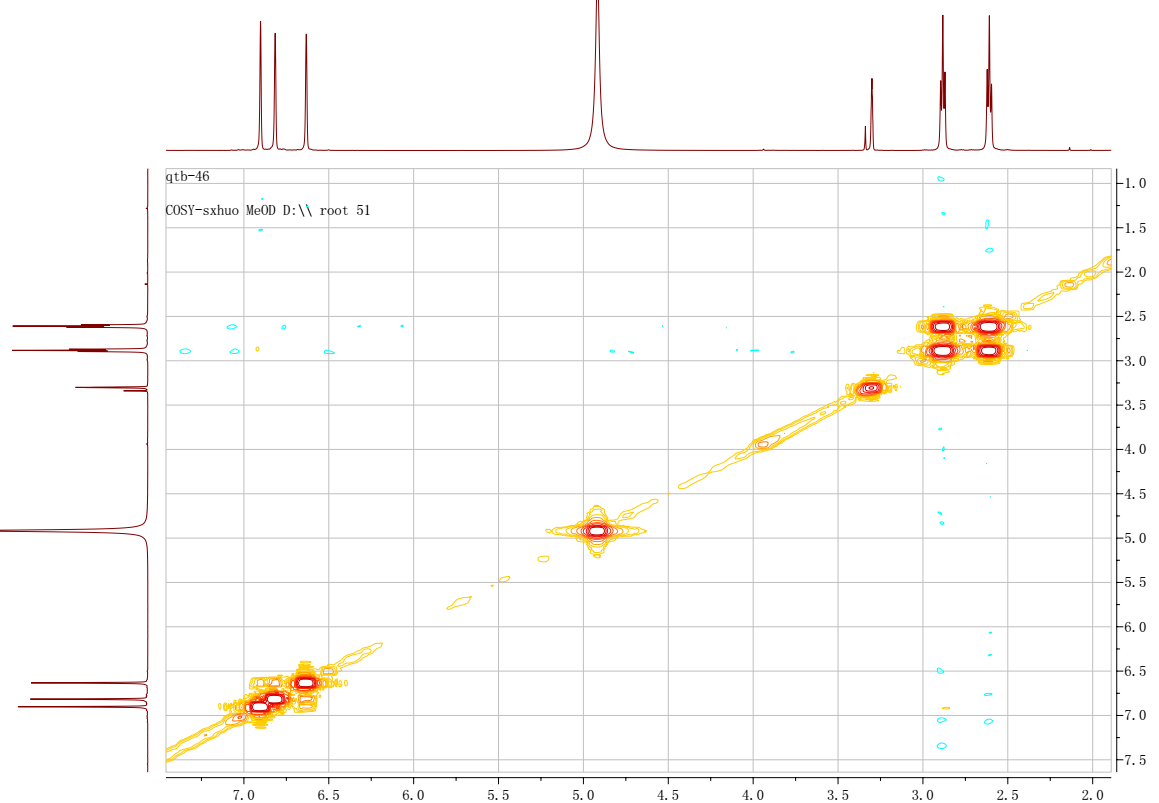


Figure S3. ^1^H-^1^H COSY spectrum of **1** in methanol-*d*_4_


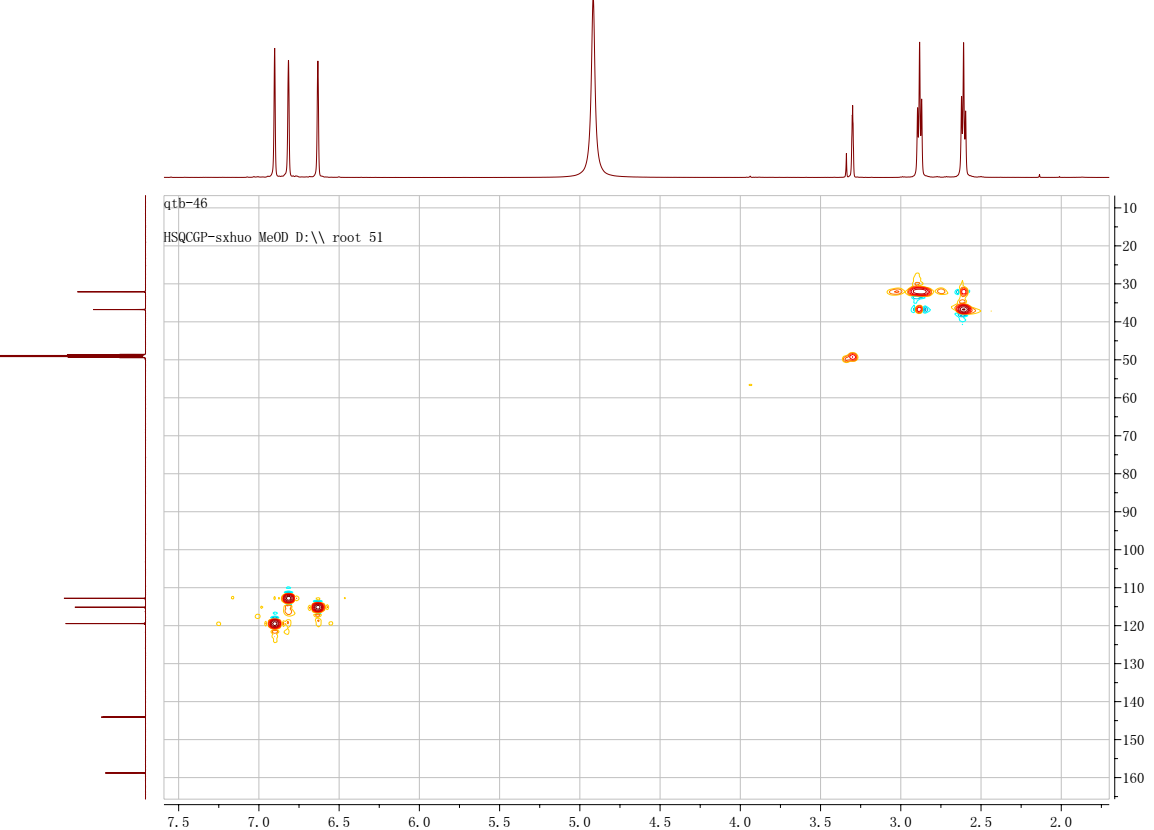


Figure S4. HSQC spectrum of **1** in methanol-*d*_4_


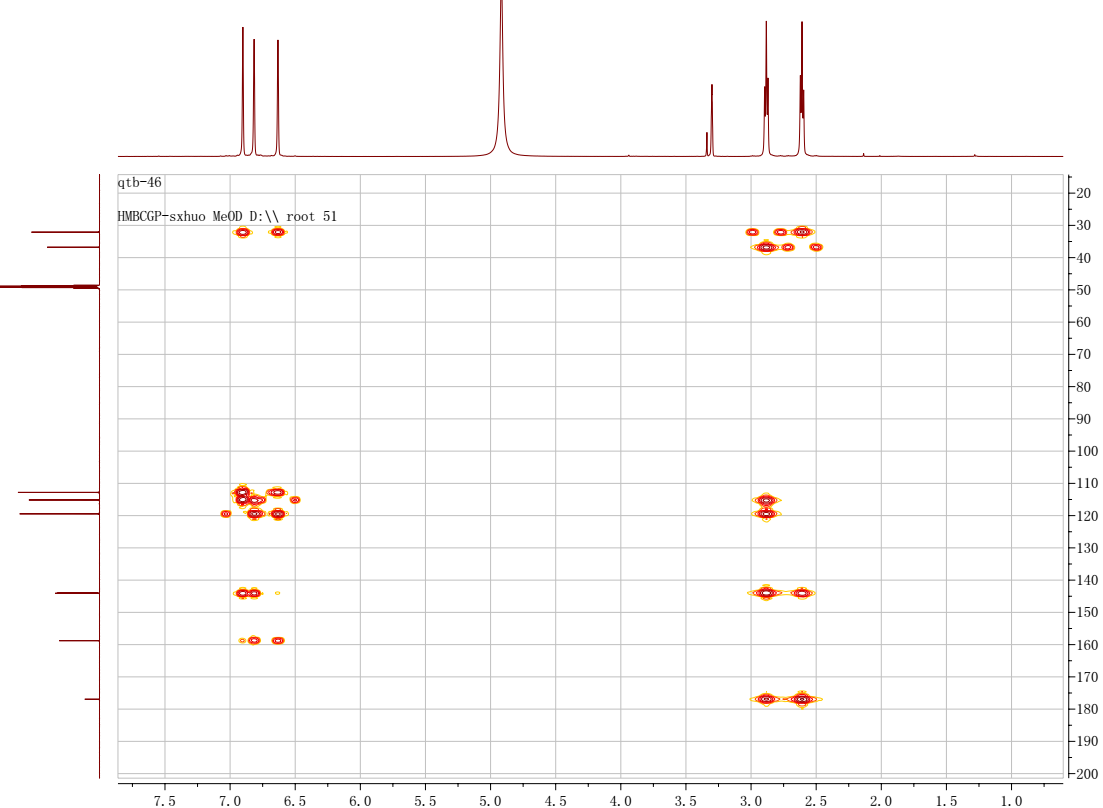


Figure S5. HMBC spectrum of **1** in methanol-*d*_4_


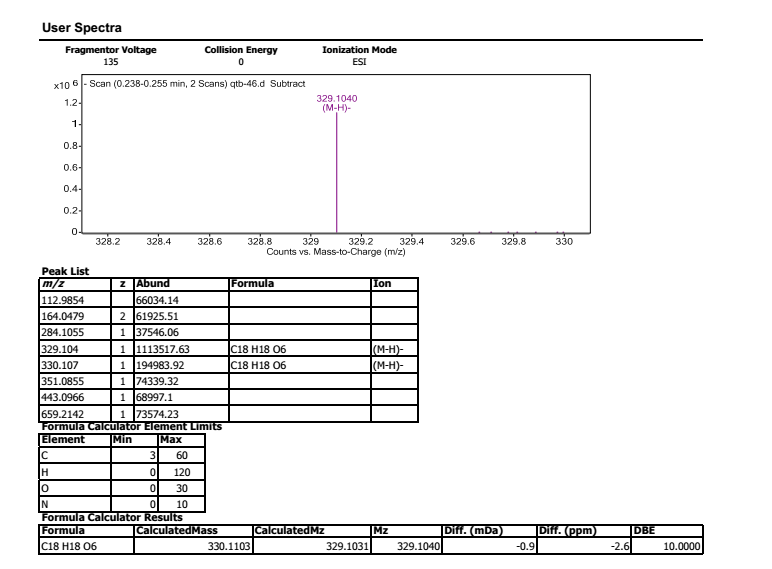


Figure S6. HREIMS of **1**


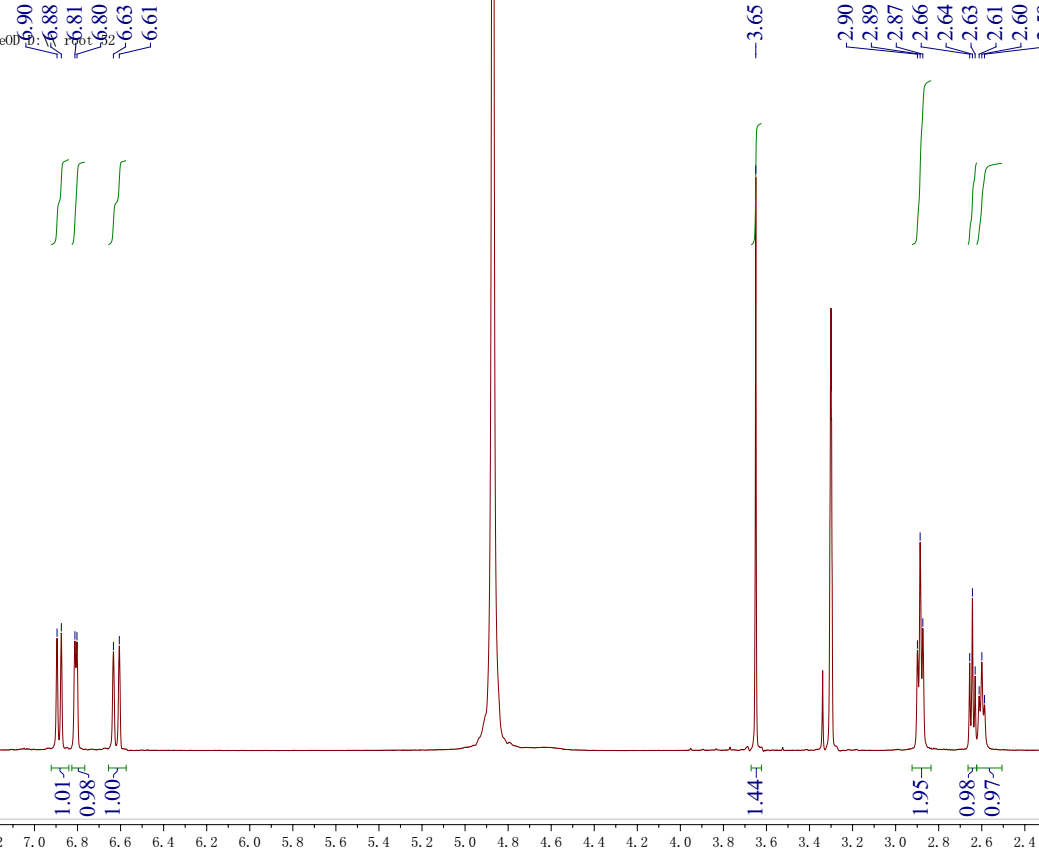


Figure S7. ^1^H NMR spectrum of **2** in methanol-*d*_4_


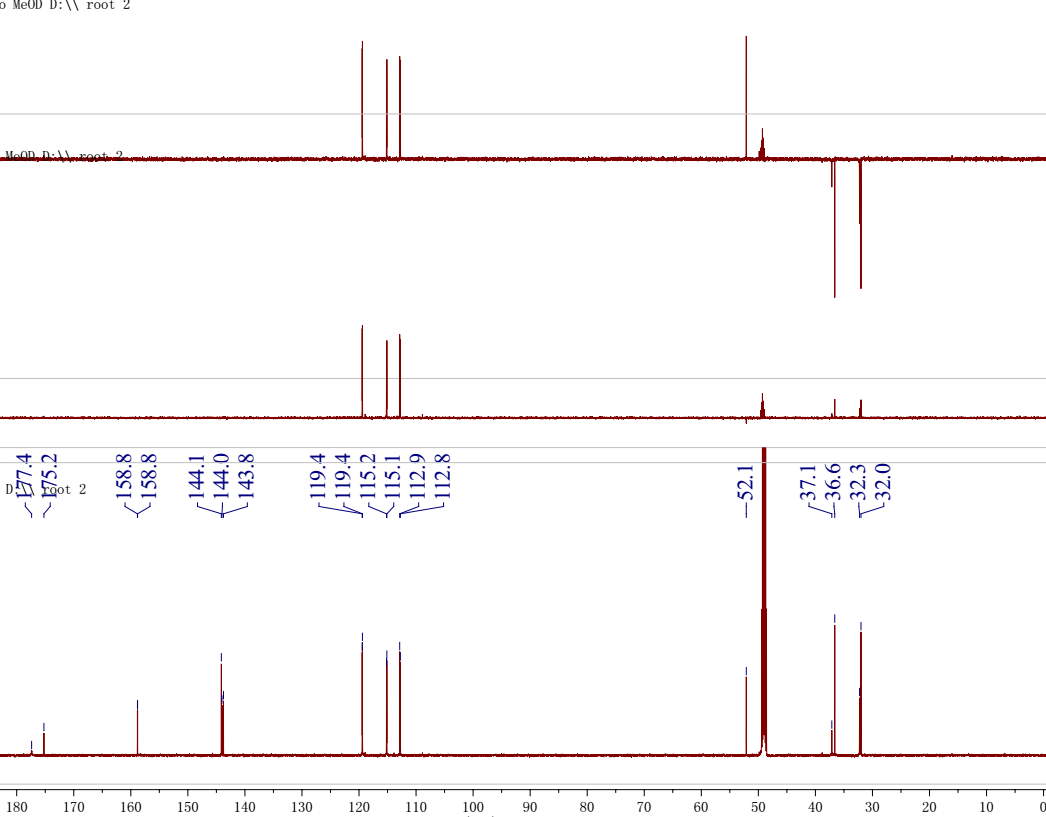


Figure S8. ^13^C NMR and DEPT spectra of **2** in methanol-*d*_4_


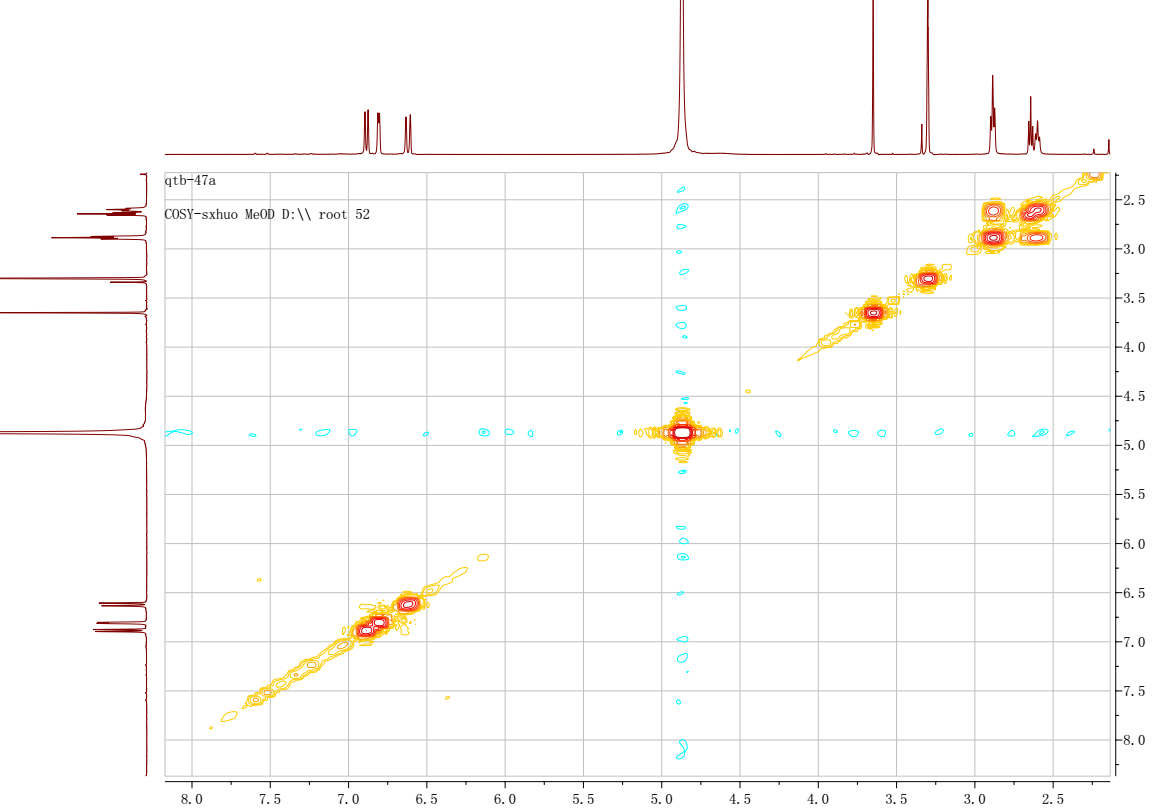


Figure S9. ^1^H-^1^H COSY spectrum of **2** in methanol-*d*_4_


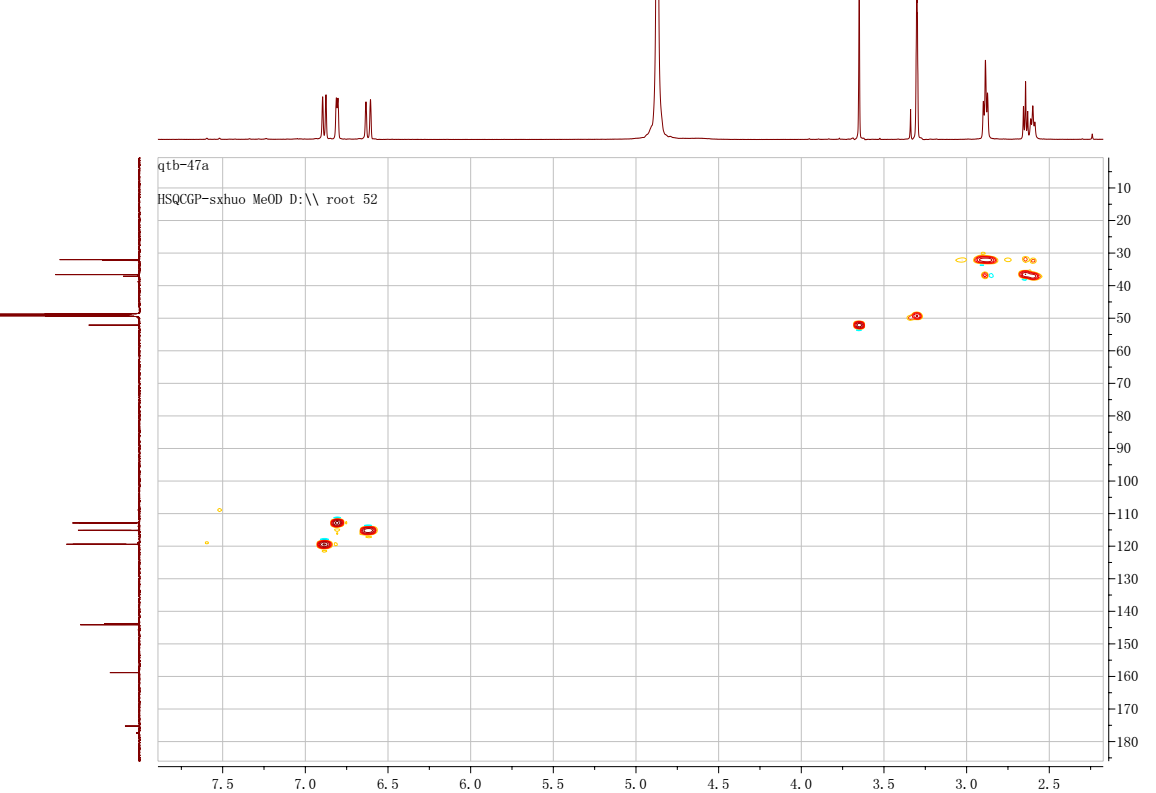


Figure S10. HSQC spectrum of **2** in methanol-*d*_4_


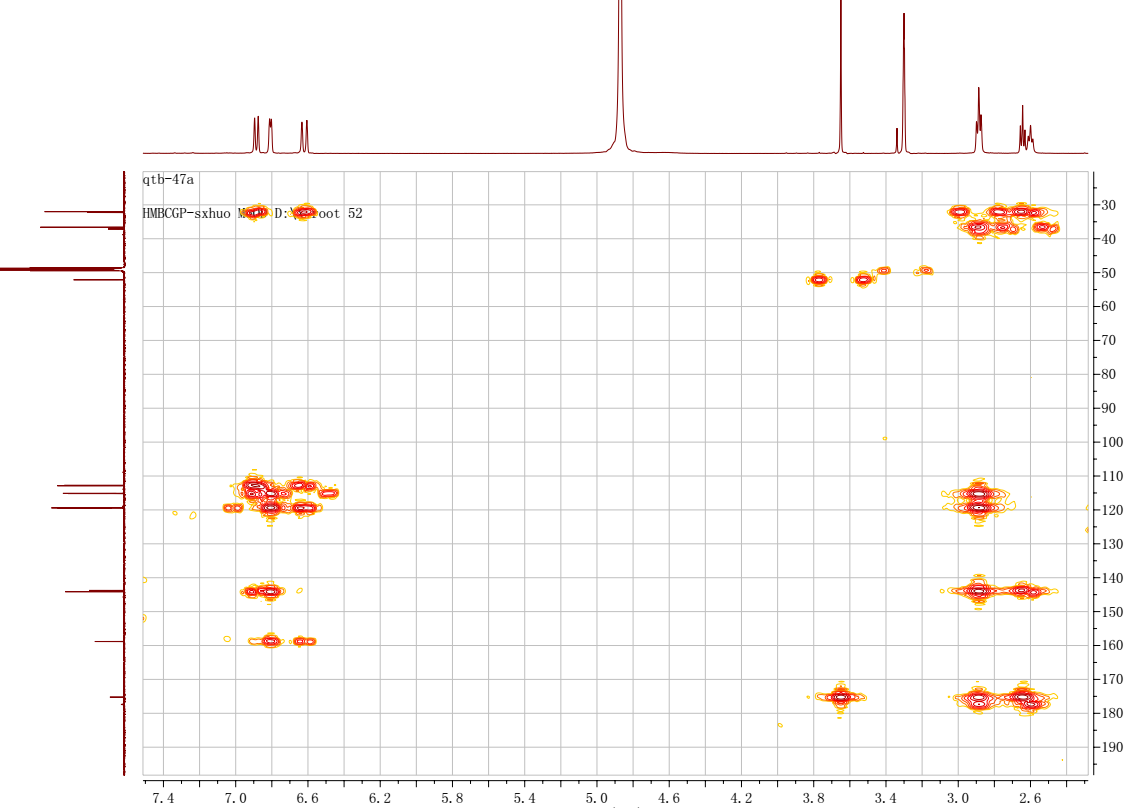


Figure S11. HMBC spectrum of **2** in methanol-*d*_4_


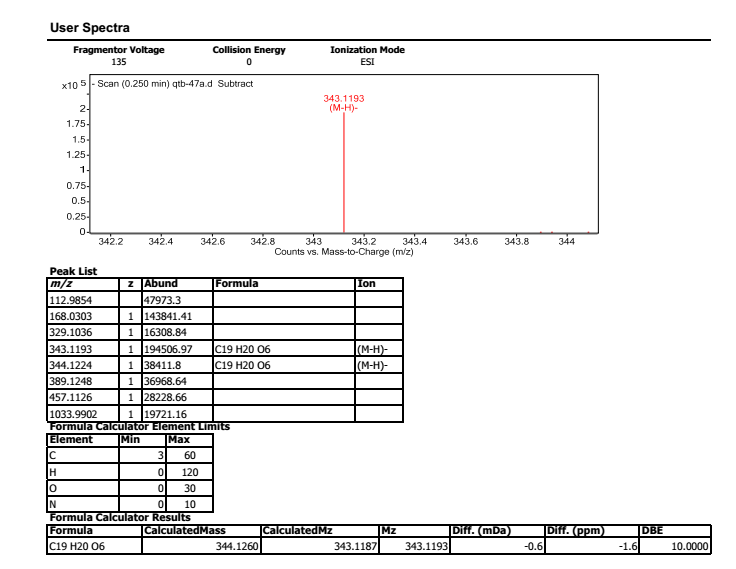


Figure S12. HREIMS of **2**


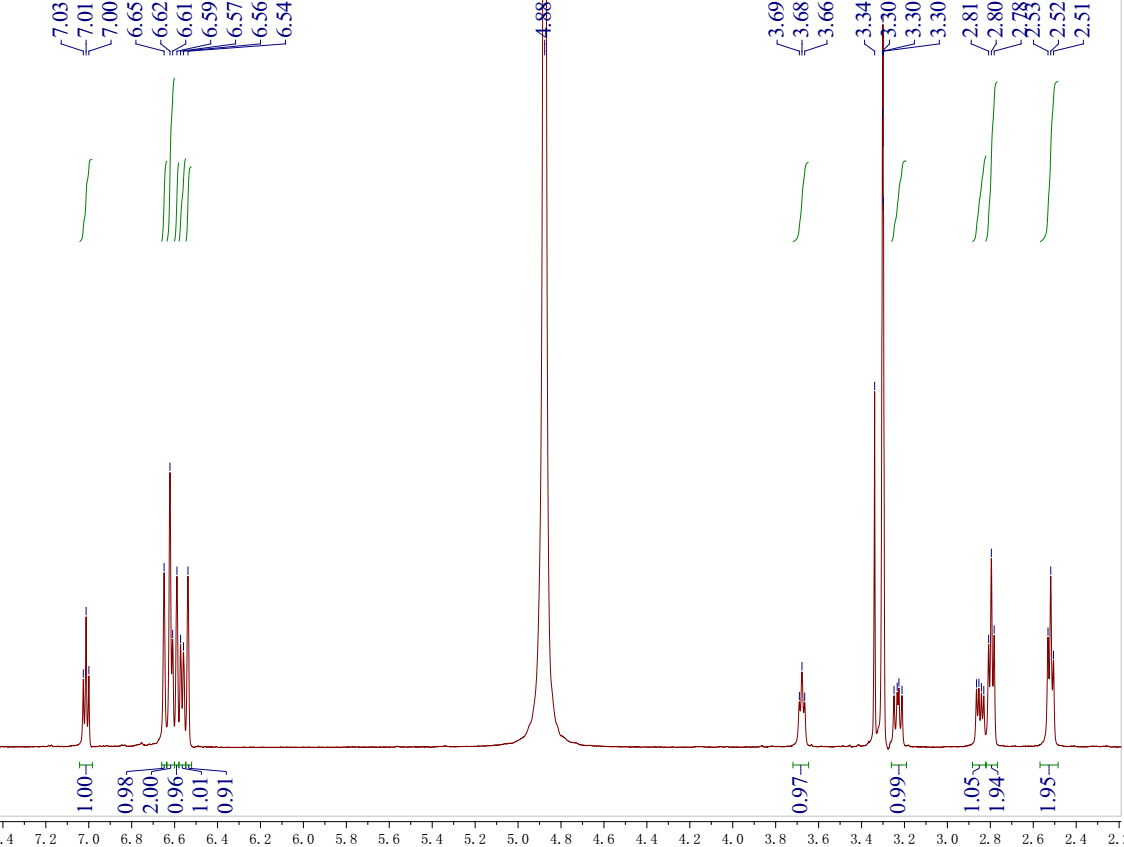


Figure S13. ^1^H NMR spectrum of **3** in methanol-*d*_4_


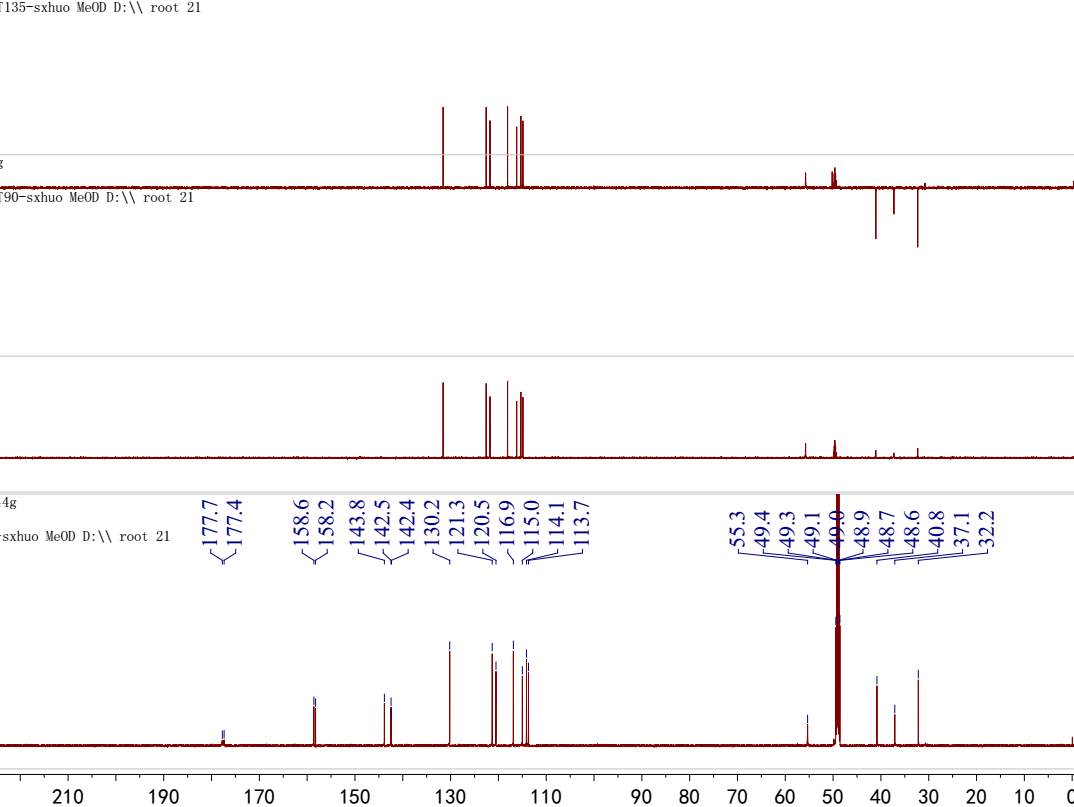


Figure S14. ^13^C NMR and DEPT spectra of **3** in methanol-*d*_4_


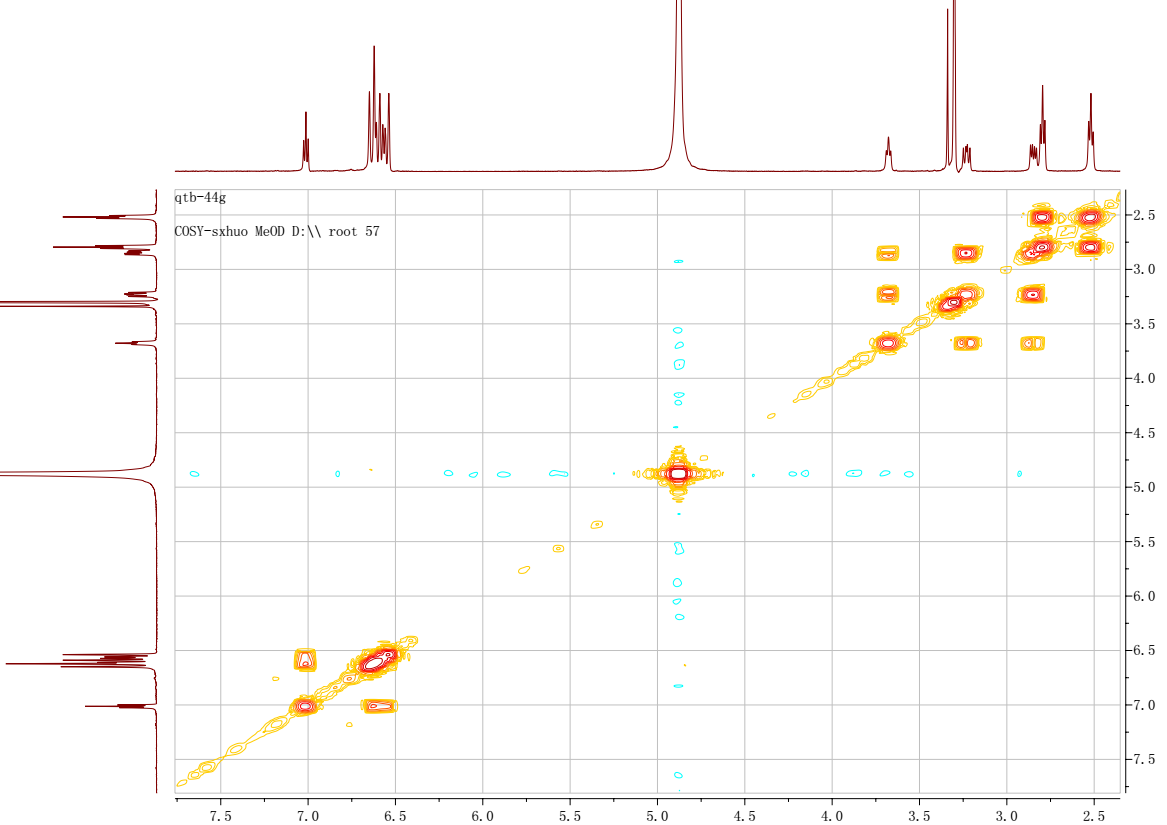


Figure S15. ^1^H-^1^H COSY spectrum of **3** in methanol-*d*_4_


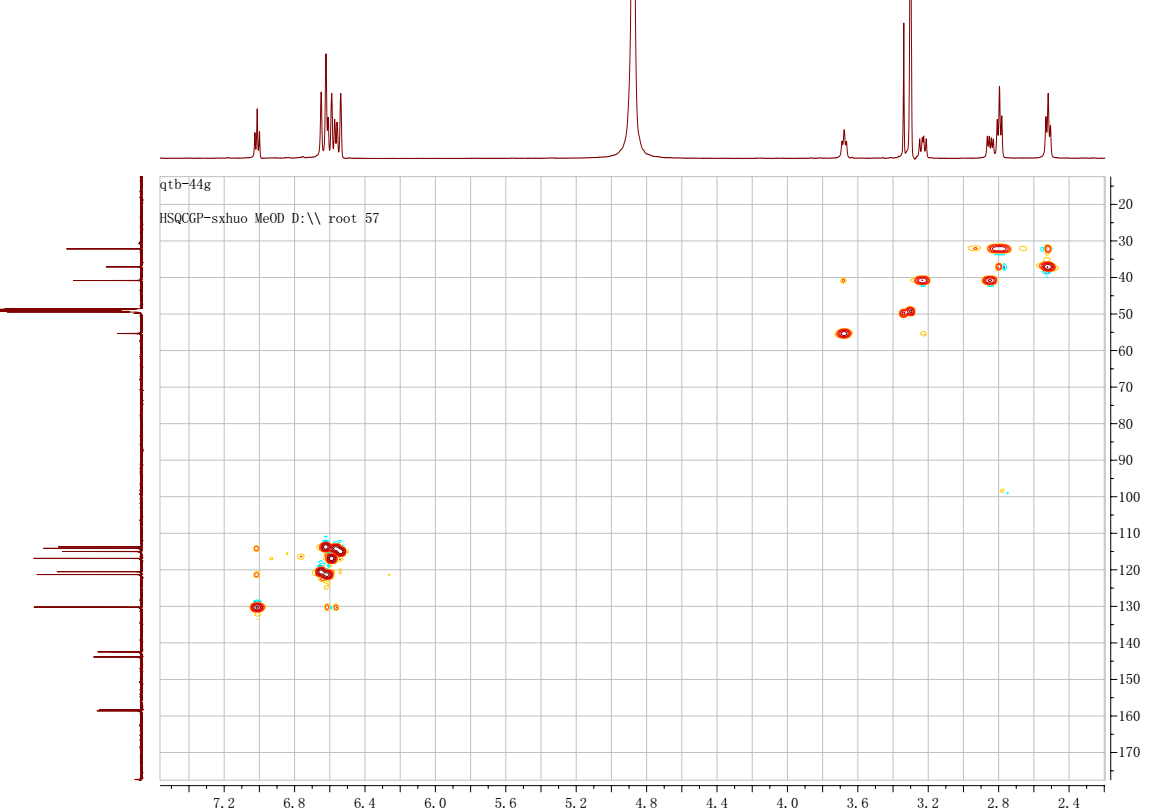


Figure S16. HSQC spectrum of **3** in methanol-*d*_4_


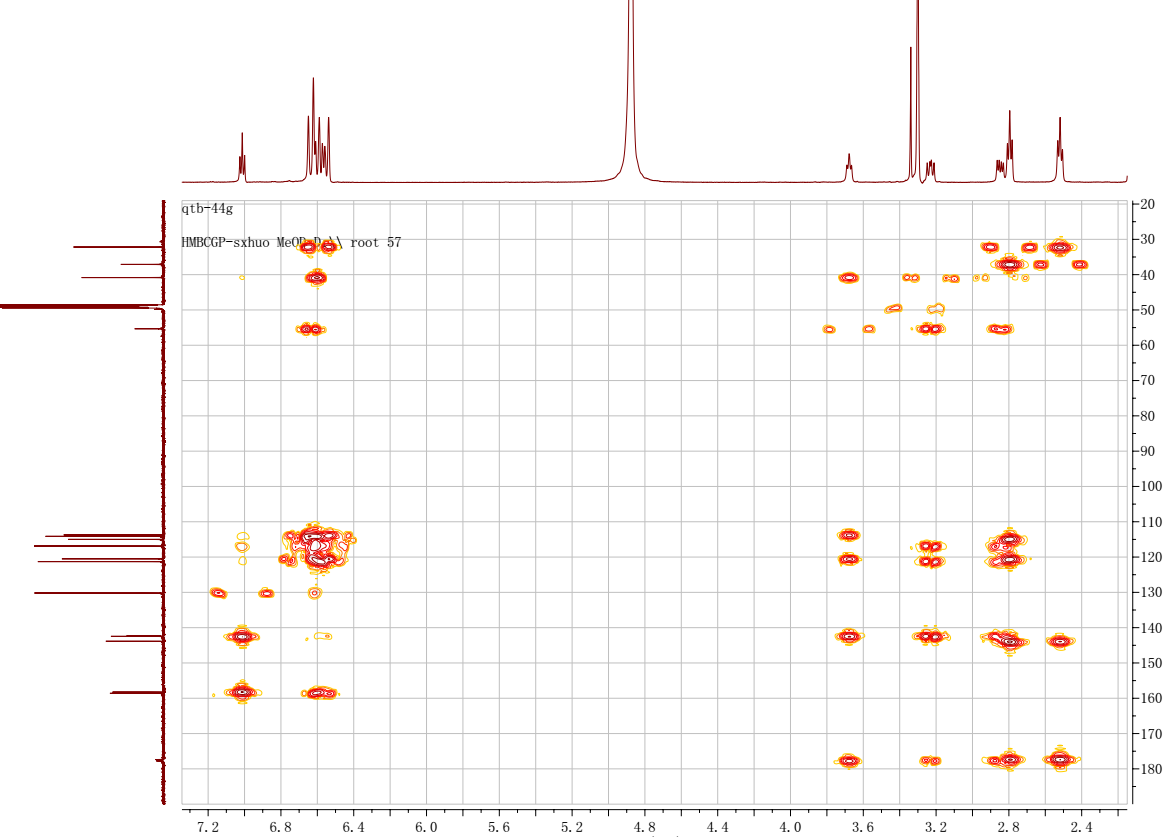


Figure S17. HMBC spectrum of **3** in methanol-*d*_4_


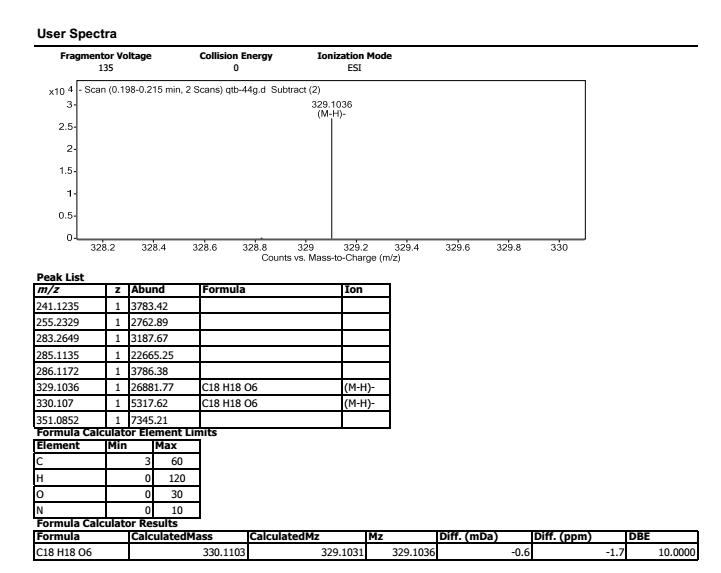


Figure S18. HREIMS of **3**

**

** **
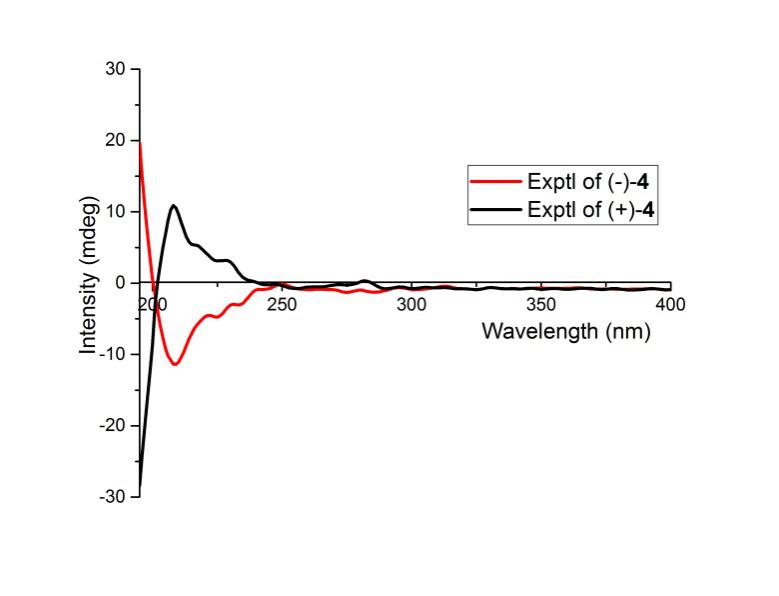
**

Figure S19. ECD comparison between **3** and **4**


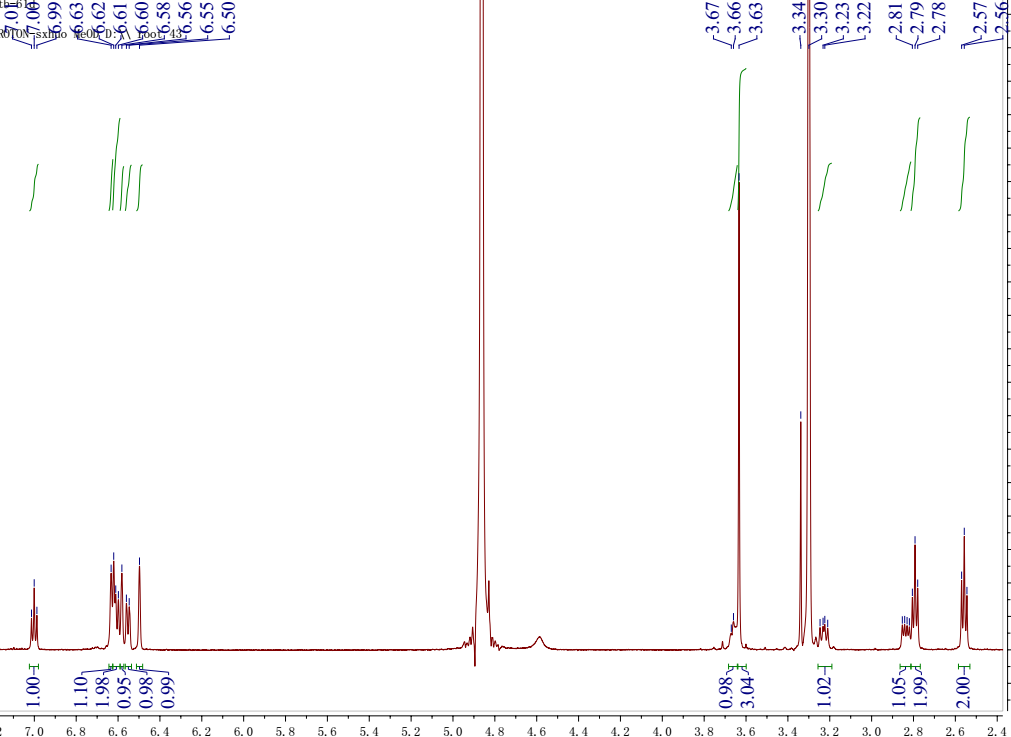


Figure S20. ^1^H NMR spectrum of **4** in methanol-*d*_4_


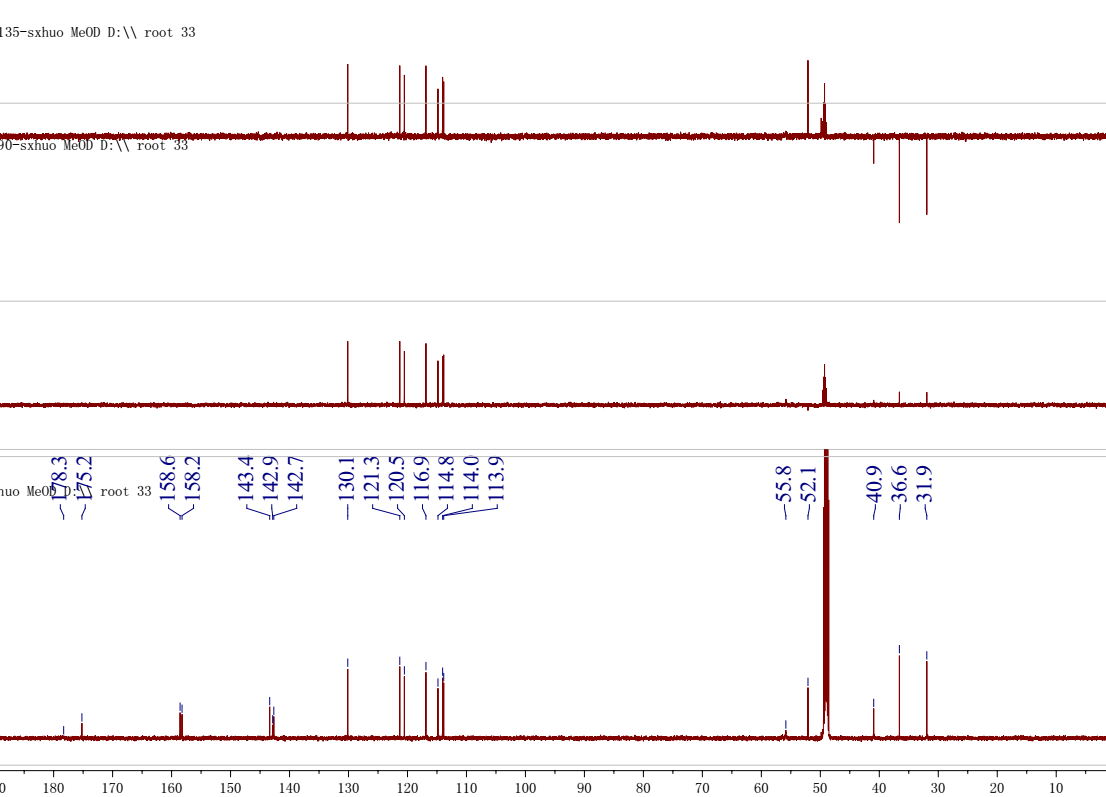


Figure S21. ^13^C NMR and DEPT spectra of **4** in methanol-*d*_4_


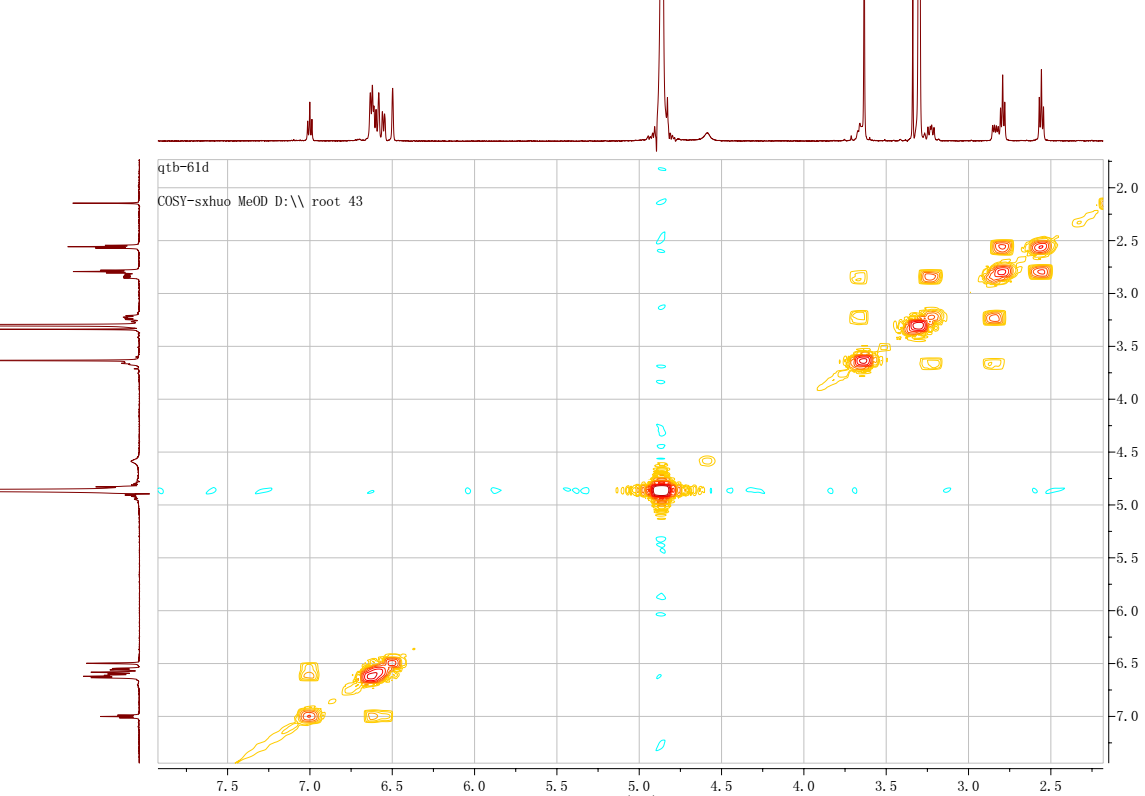


Figure S22. ^1^H-^1^H COSY spectrum of **4** in methanol-*d*_4_


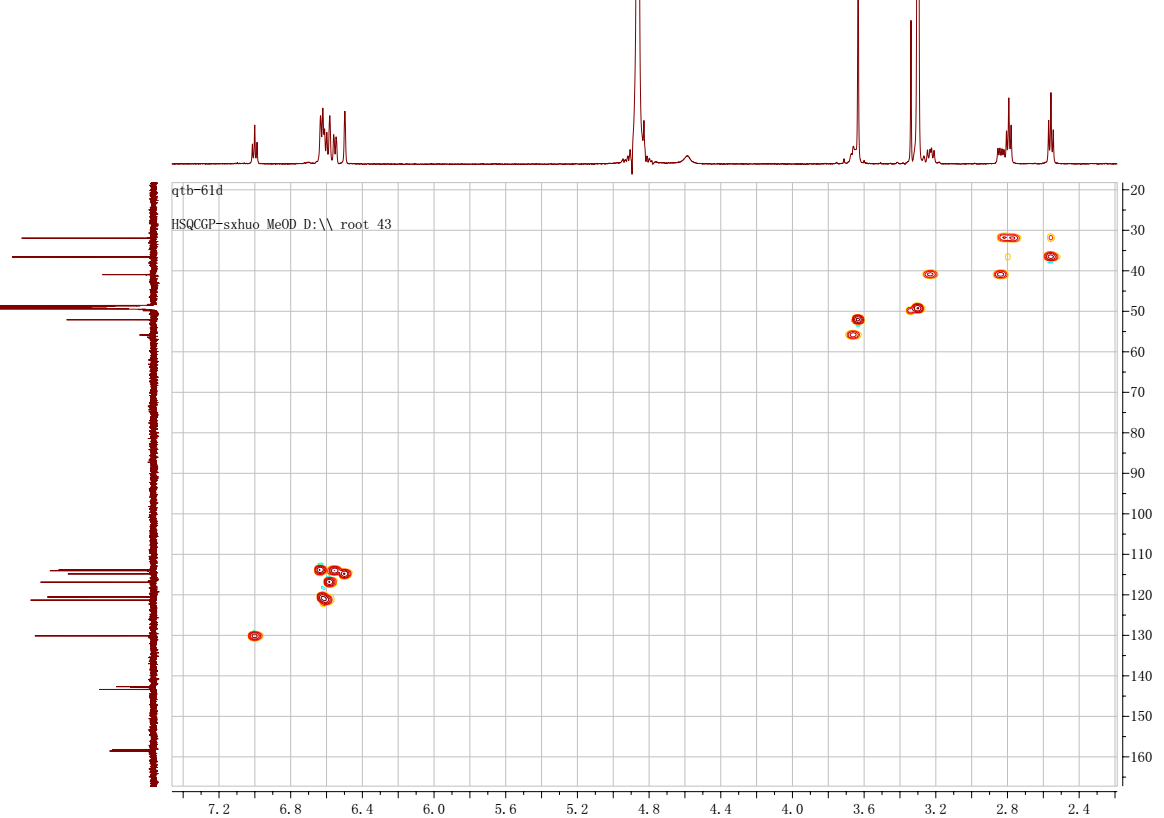


Figure S23. HSQC spectrum of **4** in methanol-*d*_4_


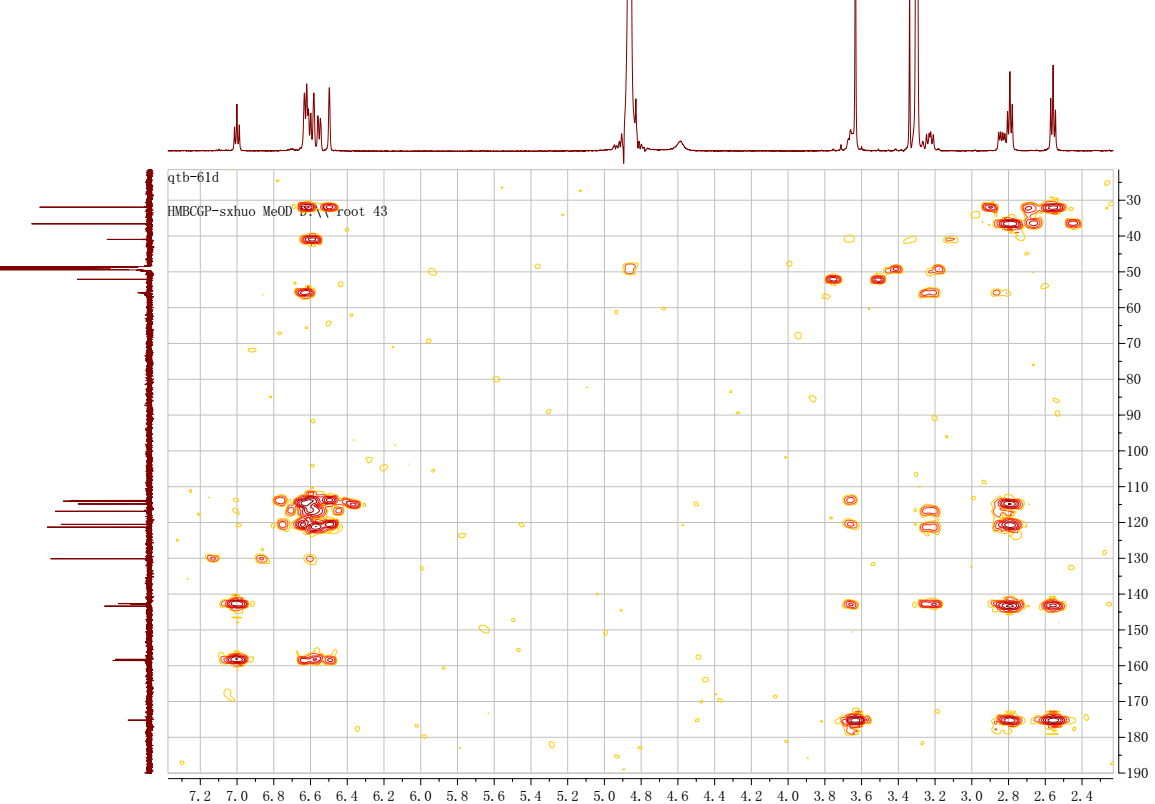


Figure S24. HMBC spectrum of **4** in methanol-*d*_4_


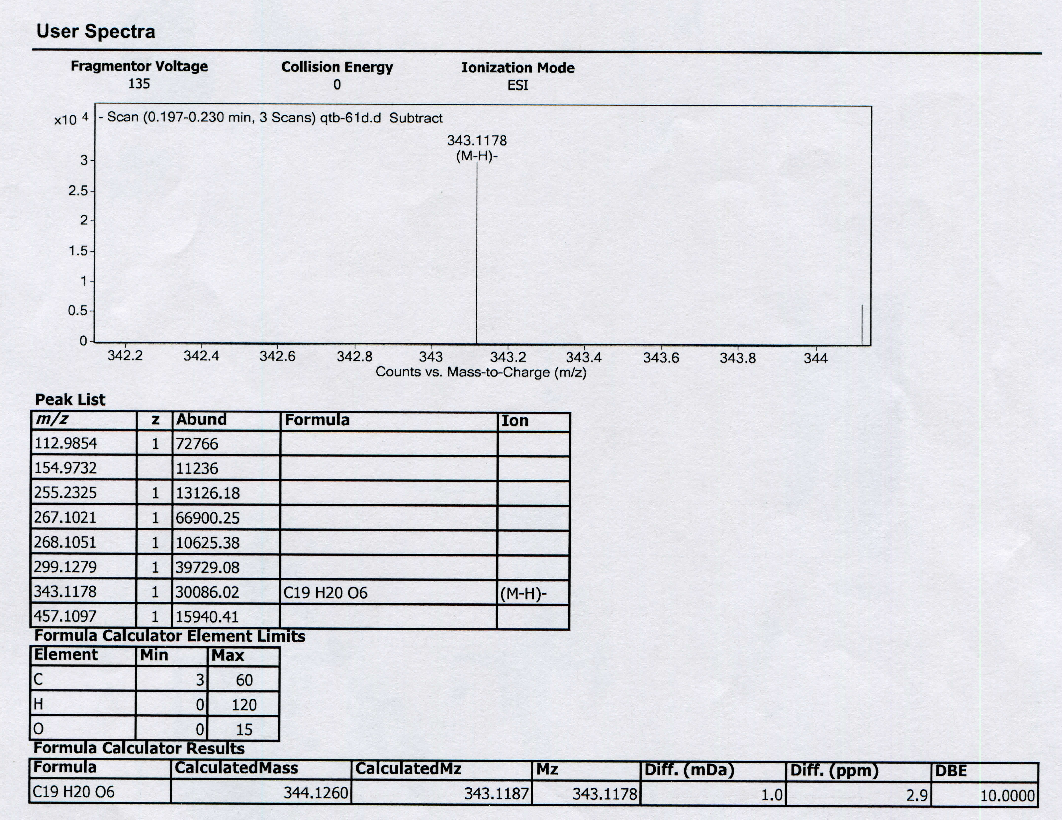


Figure S25. HREIMS of **4**


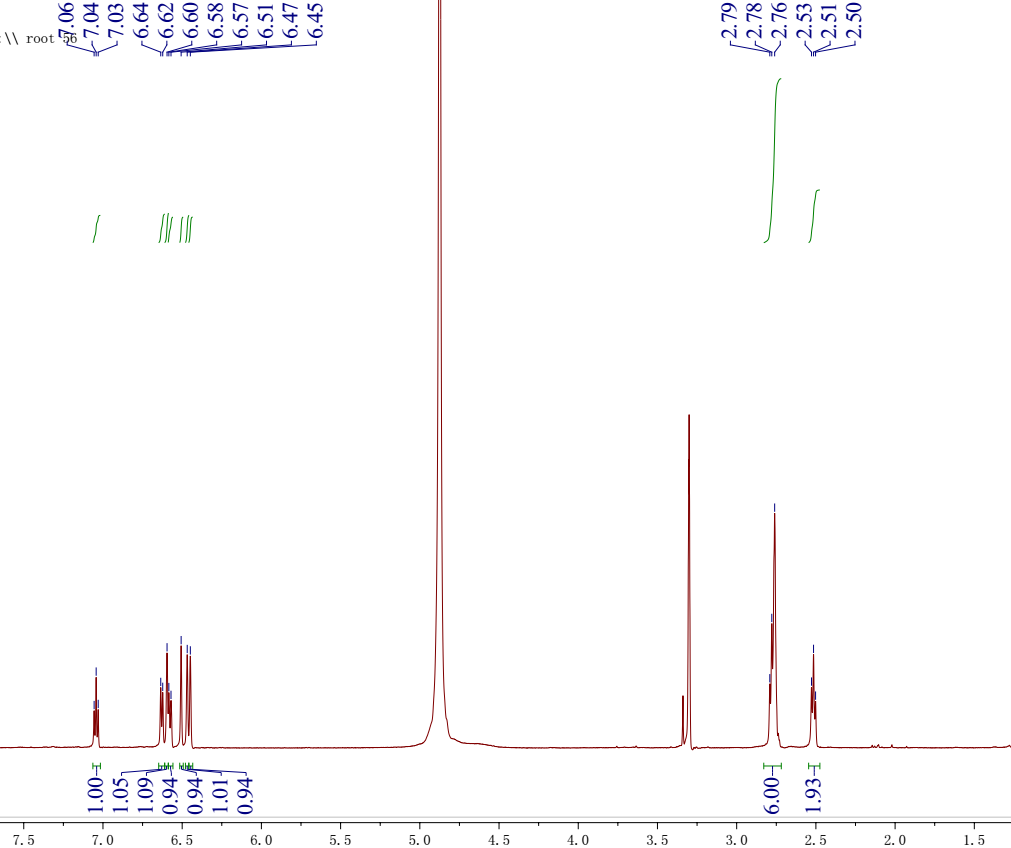


Figure S26. ^1^H NMR spectrum of **5** in methanol-*d*_4_


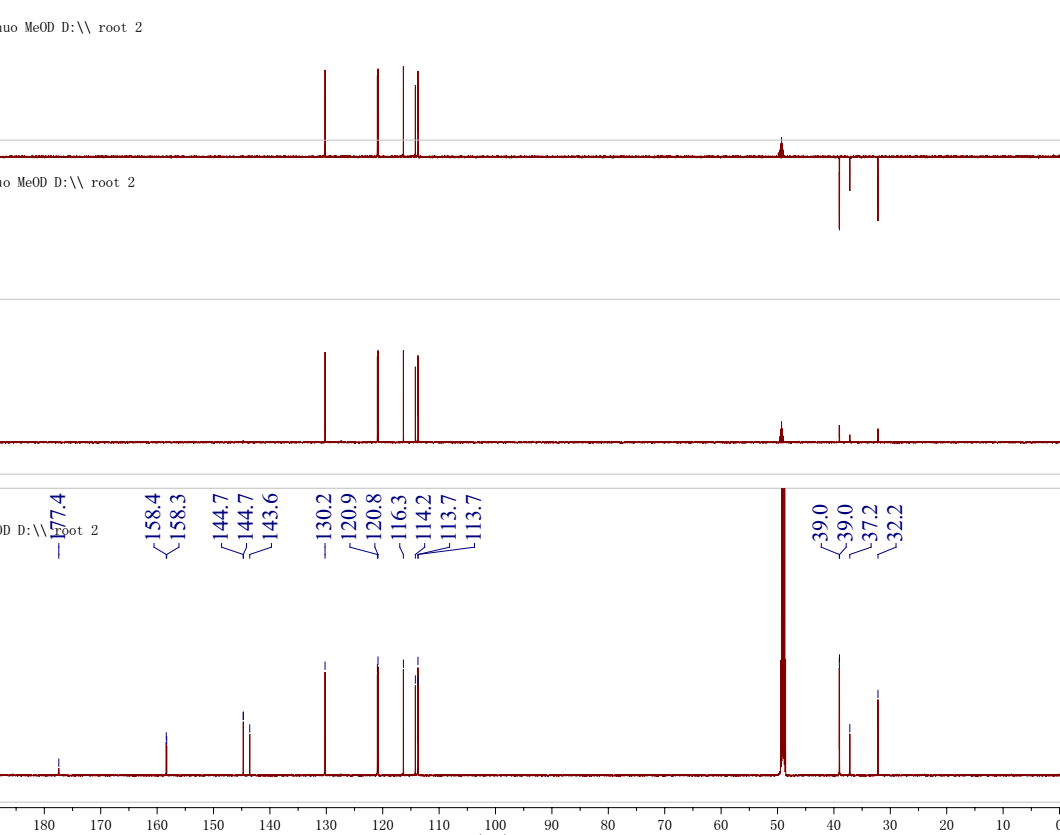


Figure S27. ^13^C NMR and DEPT spectra of **5** in methanol-*d*_4_


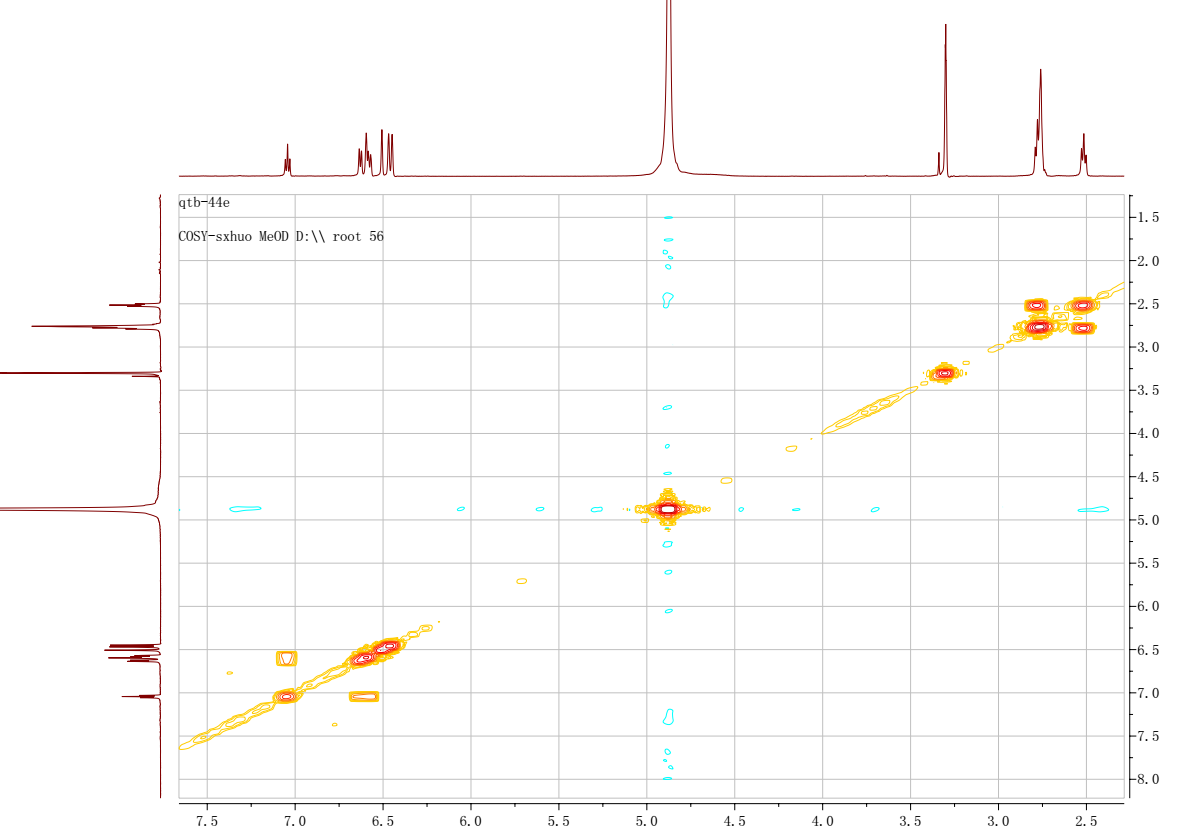


Figure S28. ^1^H-^1^H COSY spectrum of **5** in methanol-*d*_4_


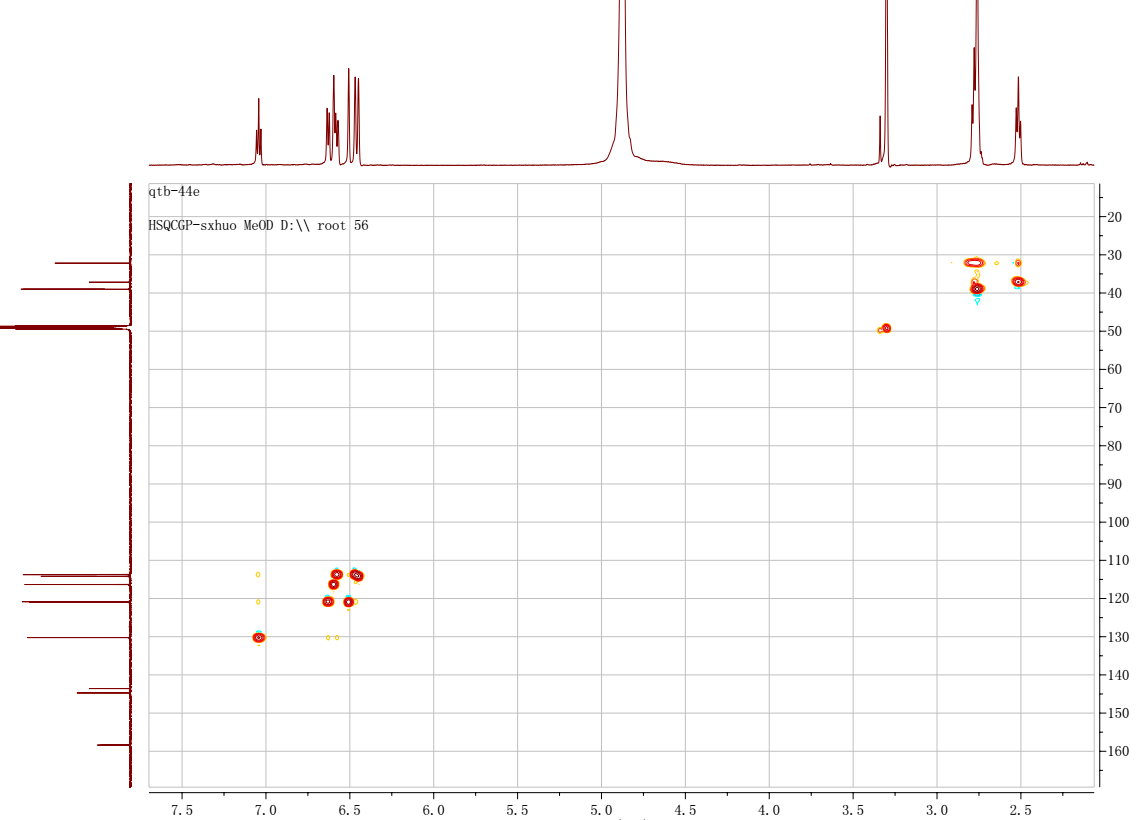


Figure S29. HSQC spectrum of **5** in methanol-*d*_4_


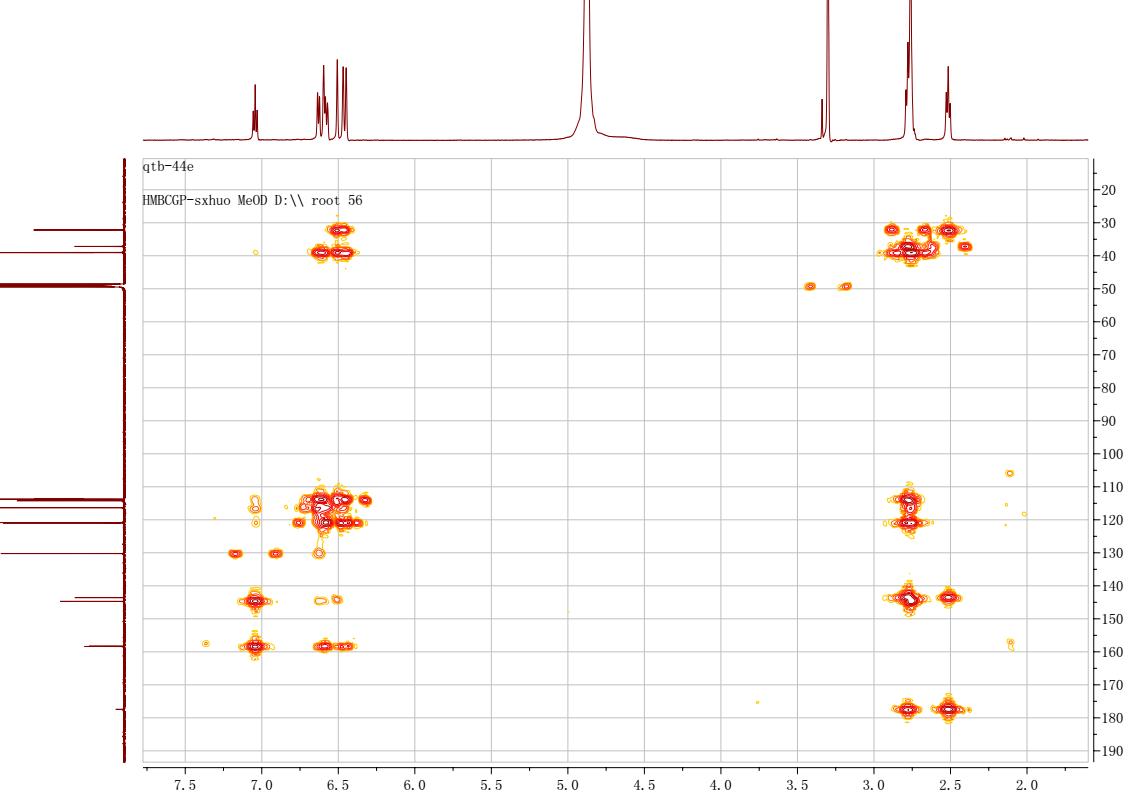


Figure S30. HMBC spectrum of **5** in methanol-*d*_4_


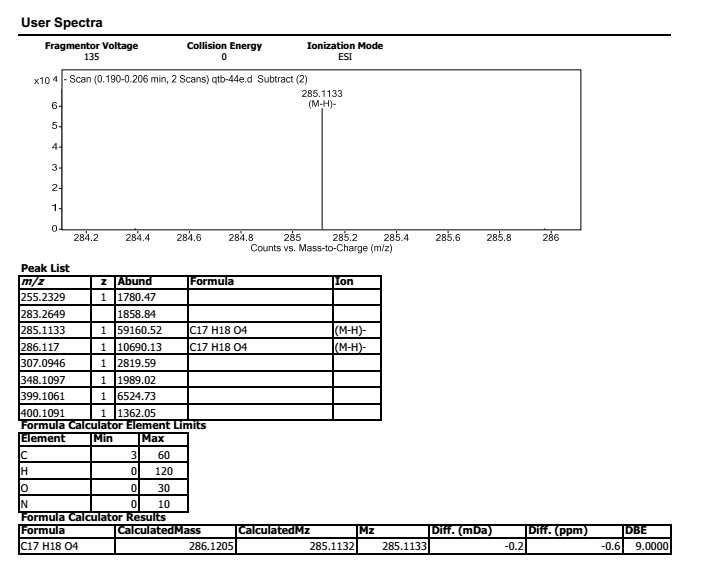


Figure S31. HREIMS of **5**


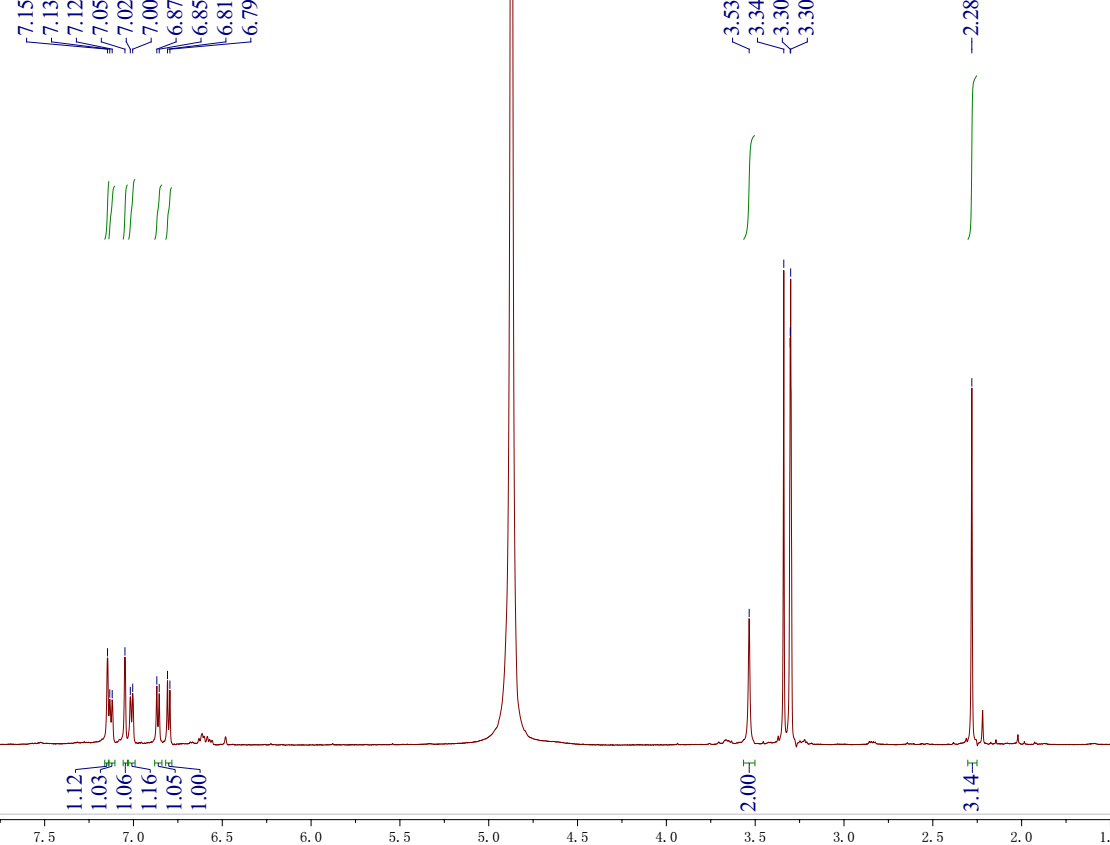


Figure S32. ^1^H NMR spectrum of **6** in methanol-*d*_4_


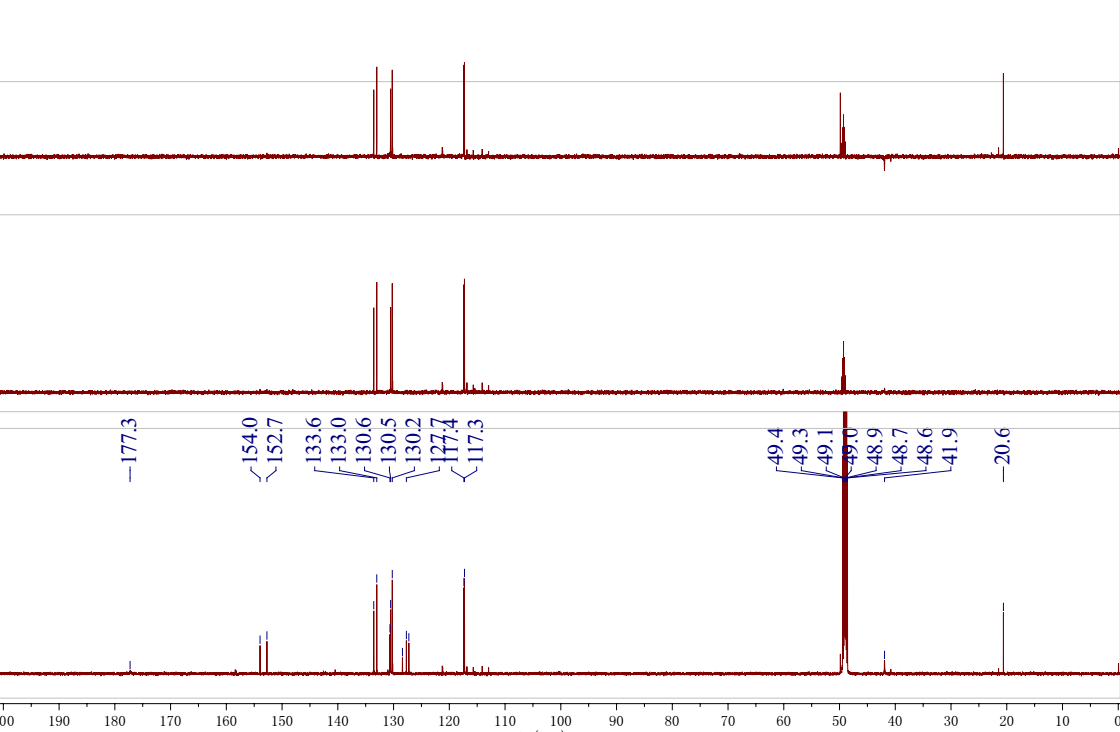


Figure S33. ^13^C NMR and DEPT spectra of **6** in methanol-*d*_4_


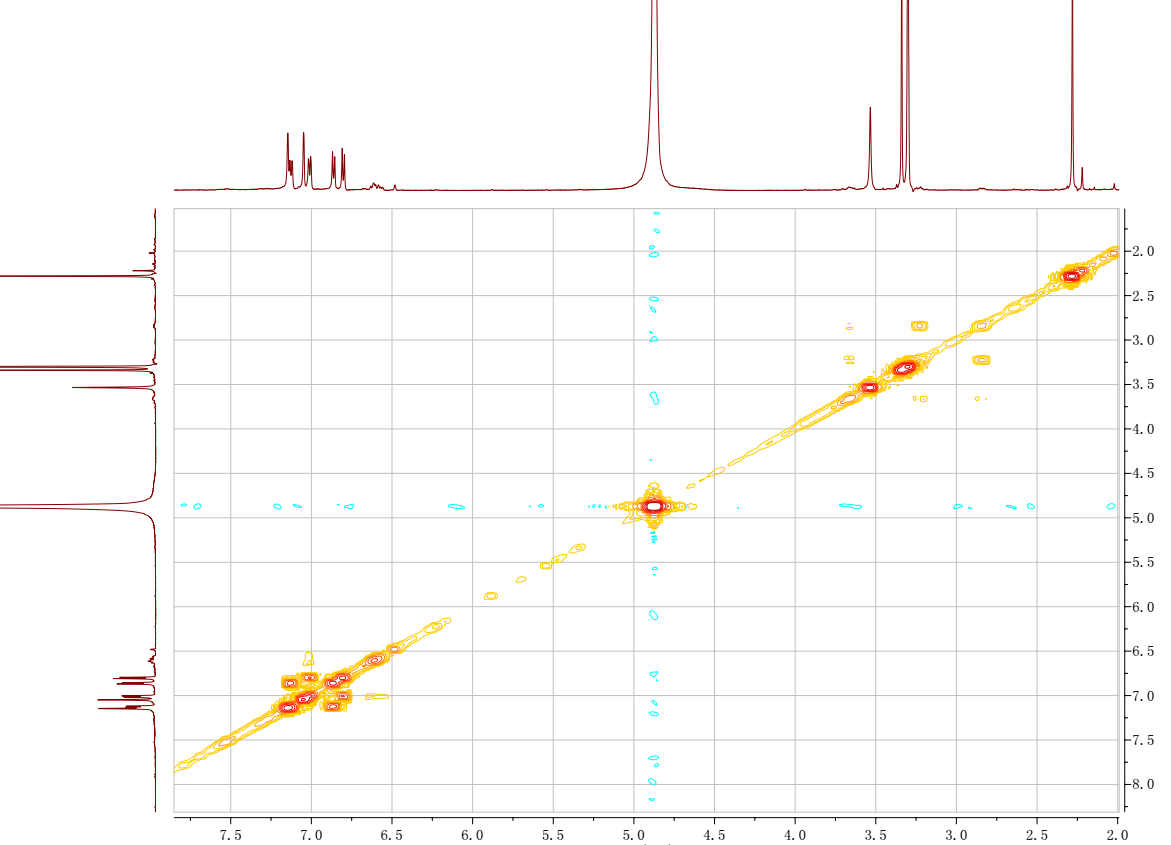


Figure S34. ^1^H-^1^H COSY spectrum of **6** in methanol-*d*_4_


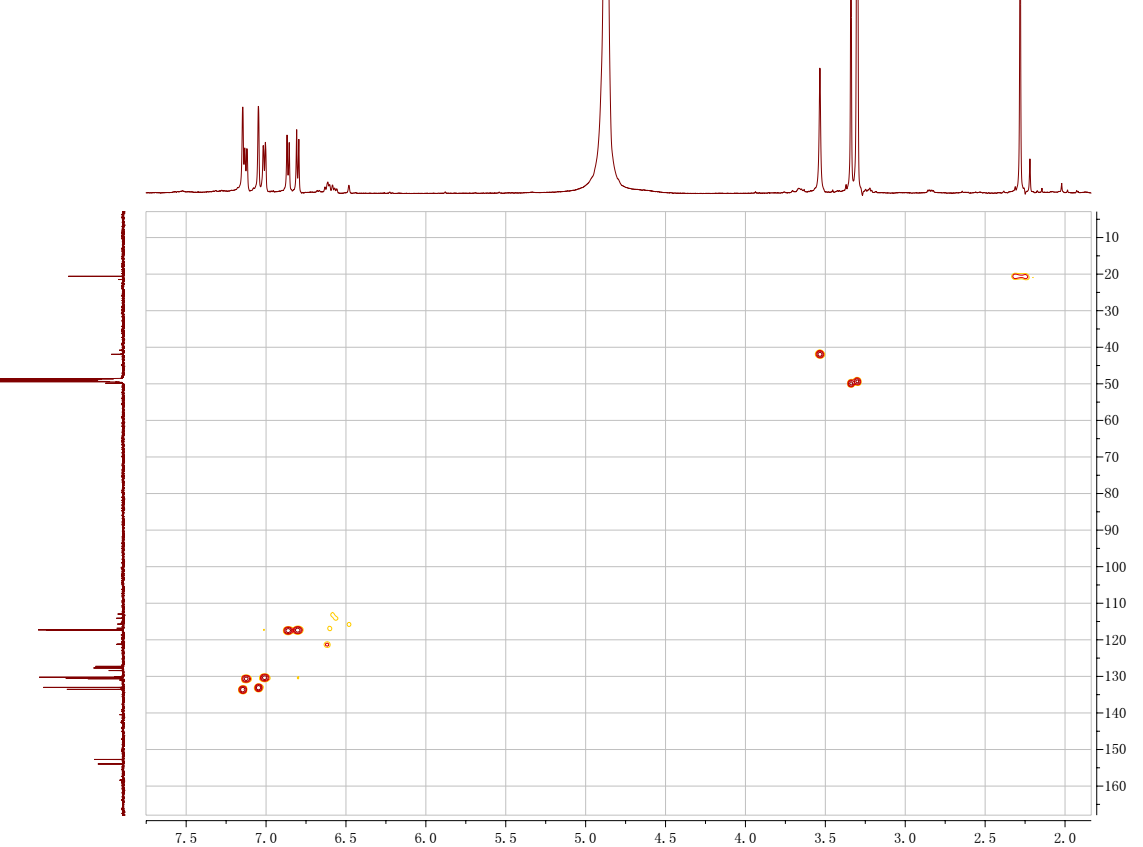


Figure S35. HSQC spectrum of **6** in methanol-*d*_4_


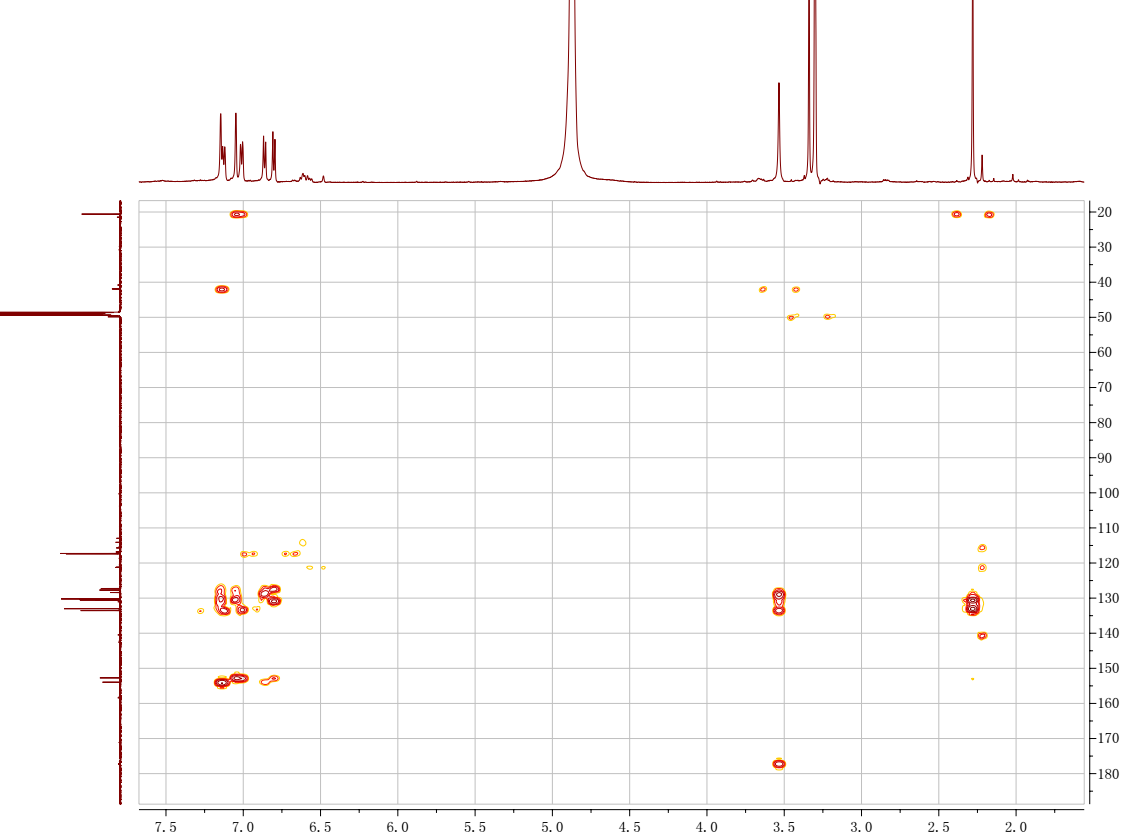


Figure S36. HMBC spectrum of **6** in methanol-*d*_4_


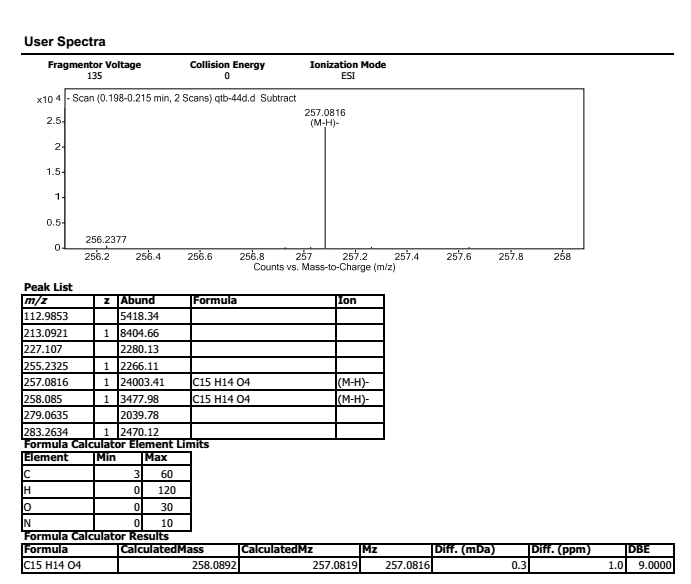


Figure S37. HREIMS of **6**


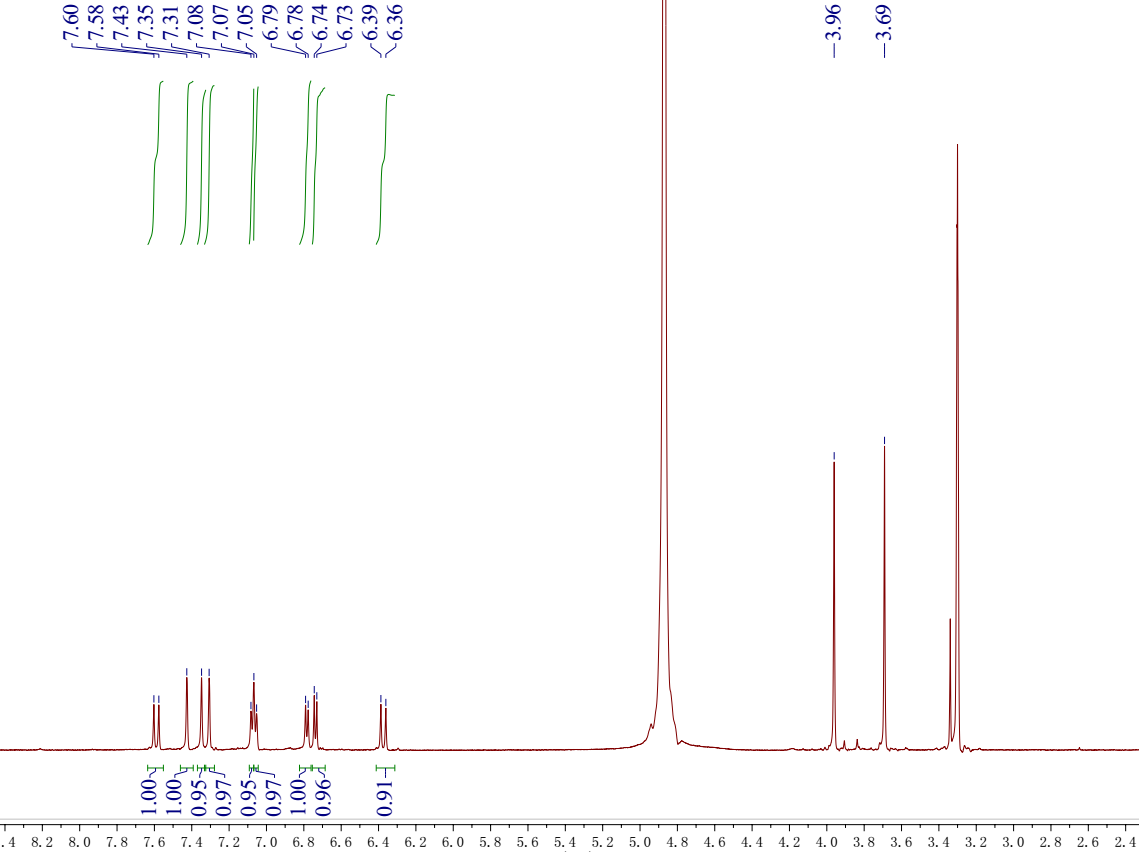


Figure S38. ^1^H NMR spectrum of **7** in methanol-*d*_4_


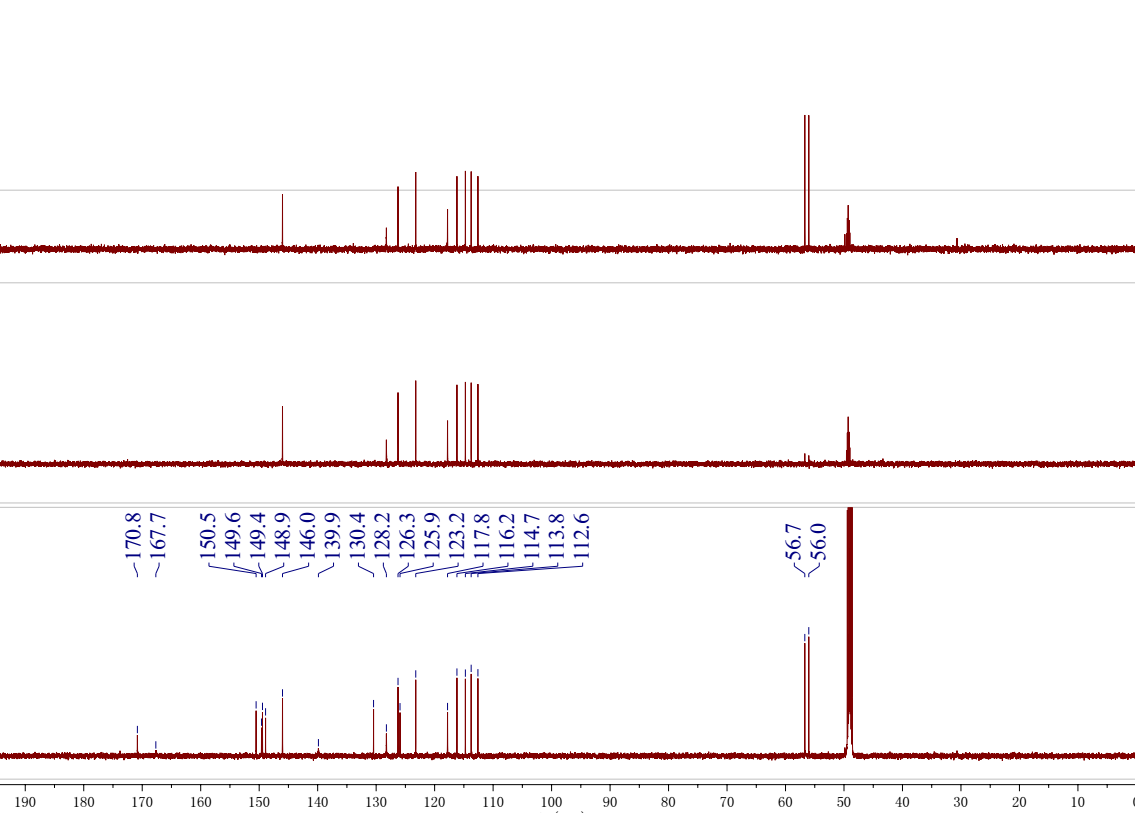


Figure S39. ^13^C NMR and DEPT spectra of **7** in methanol-*d*_4_


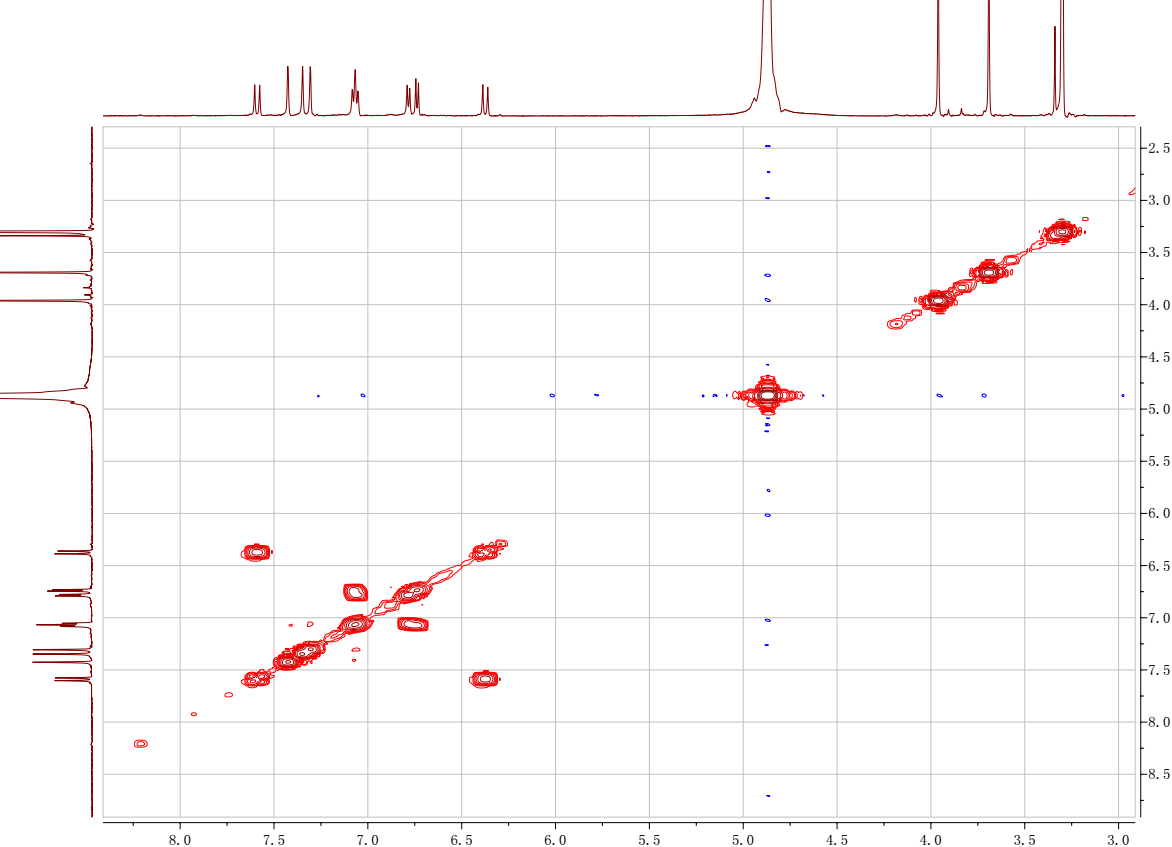


Figure S40. ^1^H-^1^H COSY spectrum of **7** in methanol-*d*_4_


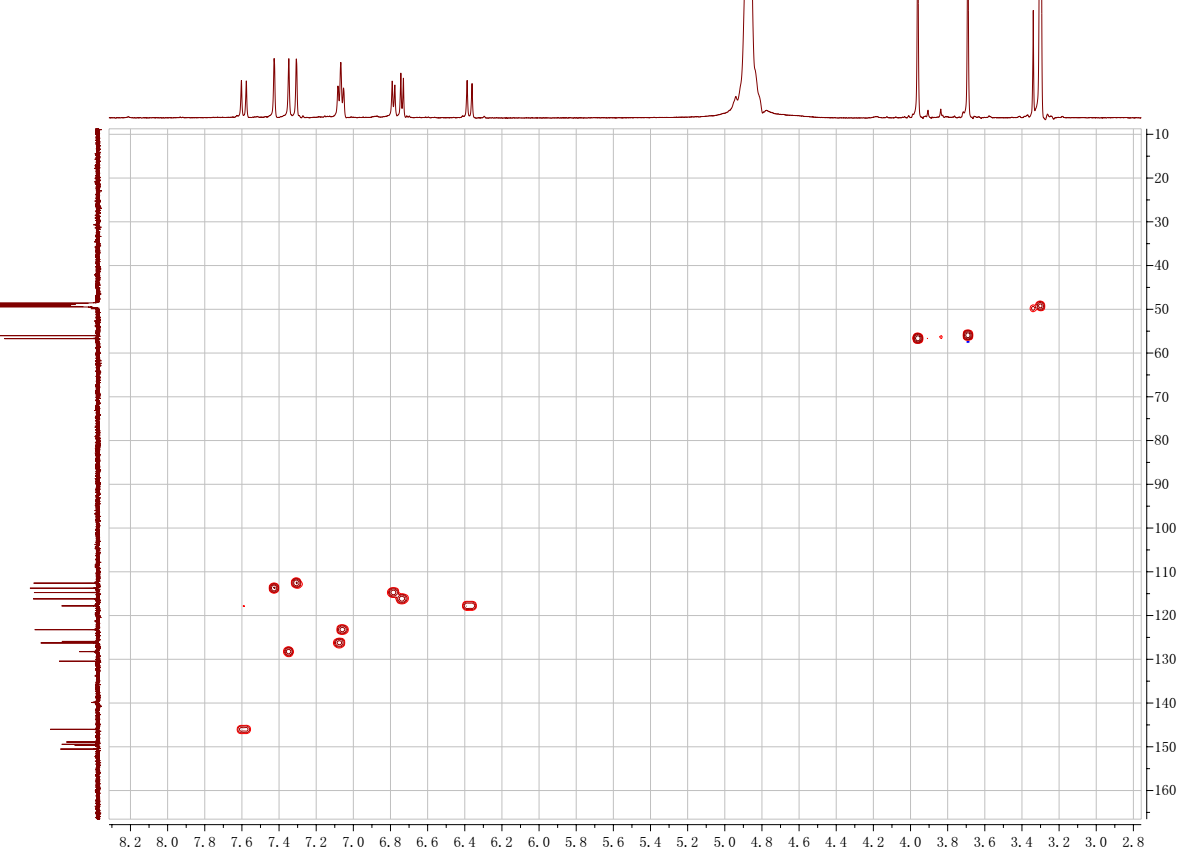


Figure S41. HSQC spectrum of **7** in methanol-*d*_4_


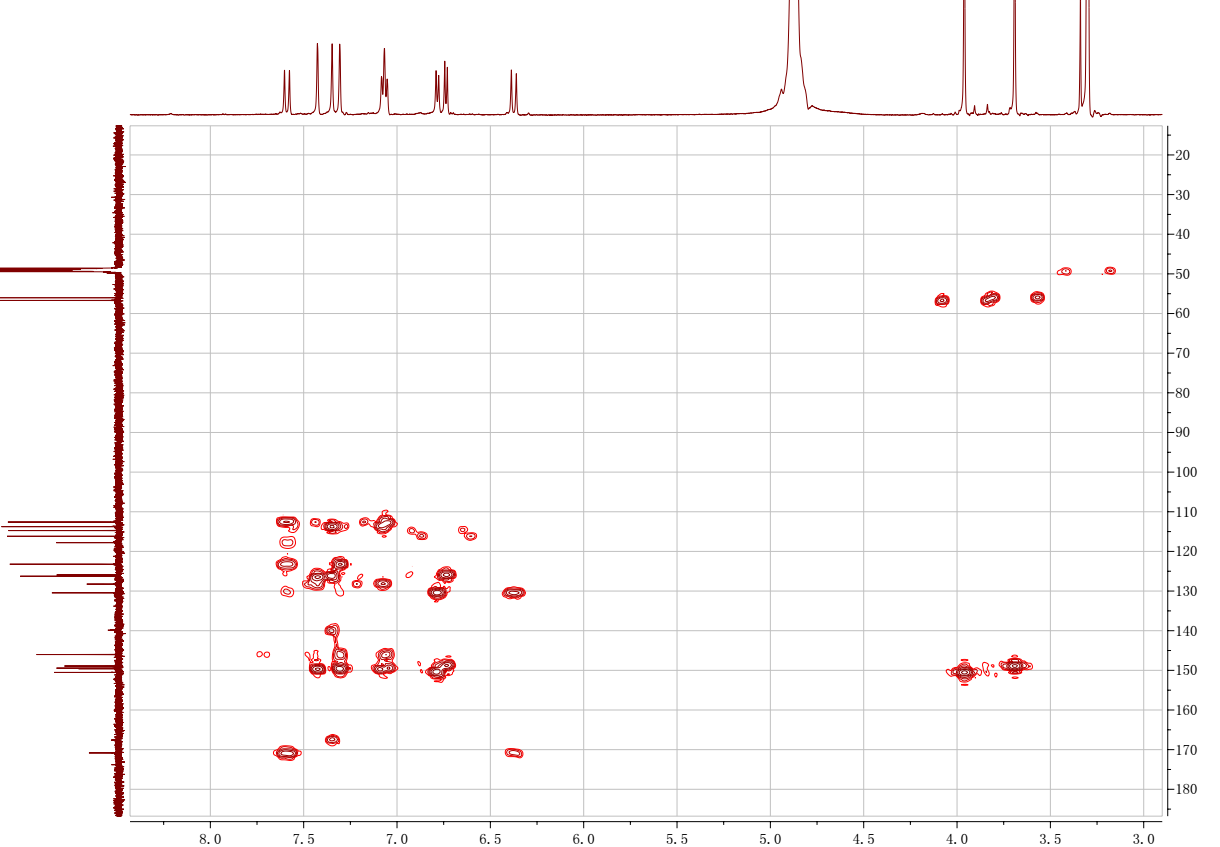


Figure S42. HMBC spectrum of **7** in methanol-*d*_4_


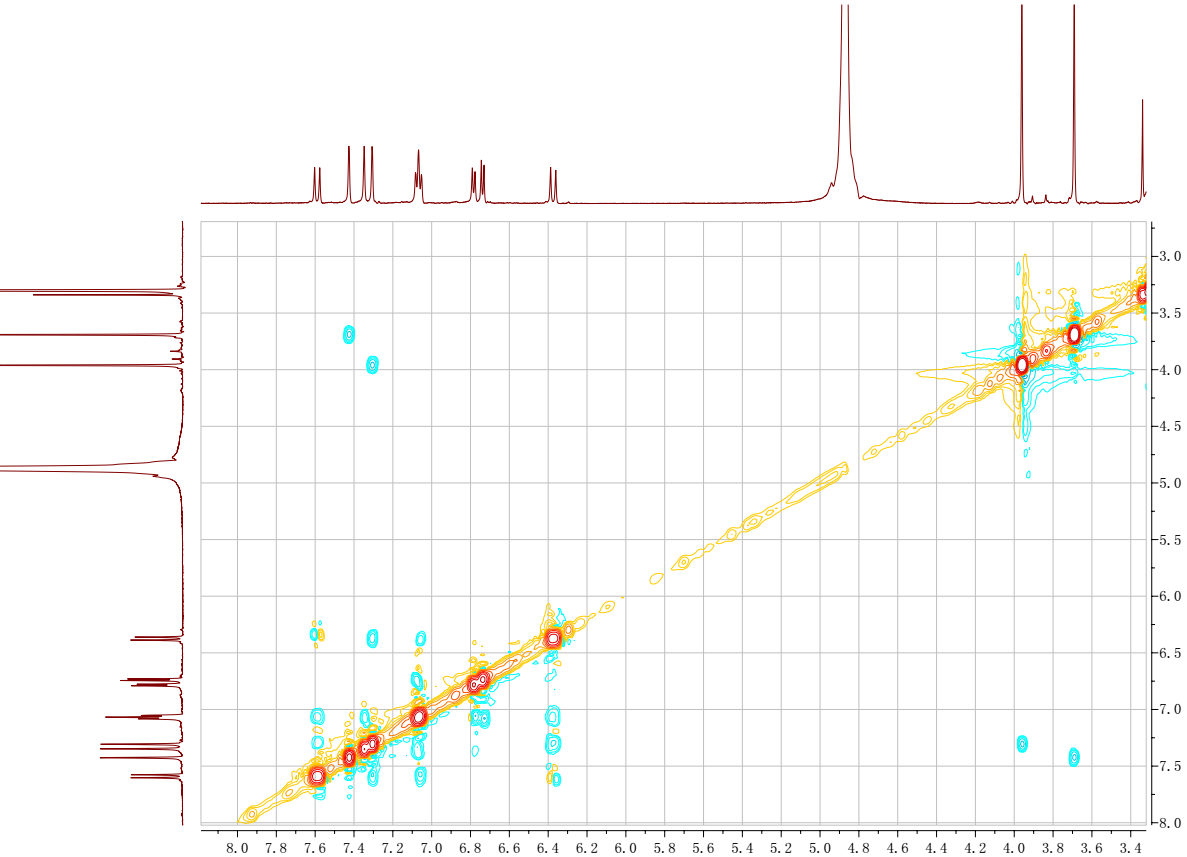


Figure S43. ROESY spectrum of **7** in methanol-*d*_4_


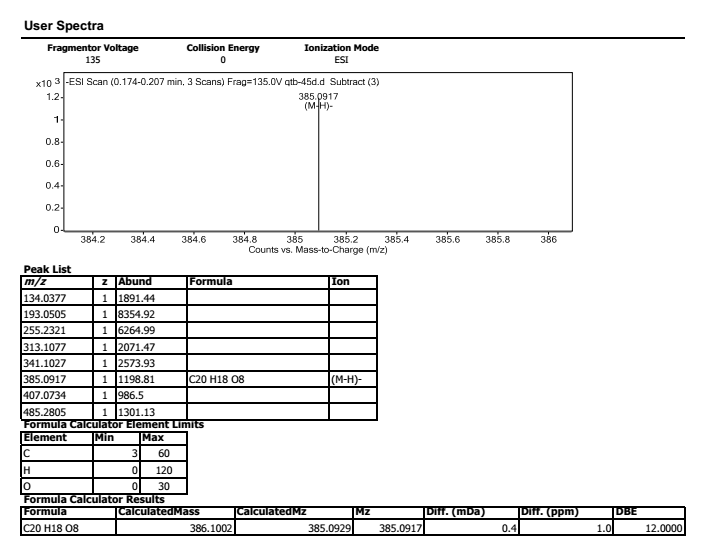


Figure S44. HREIMS of **7**


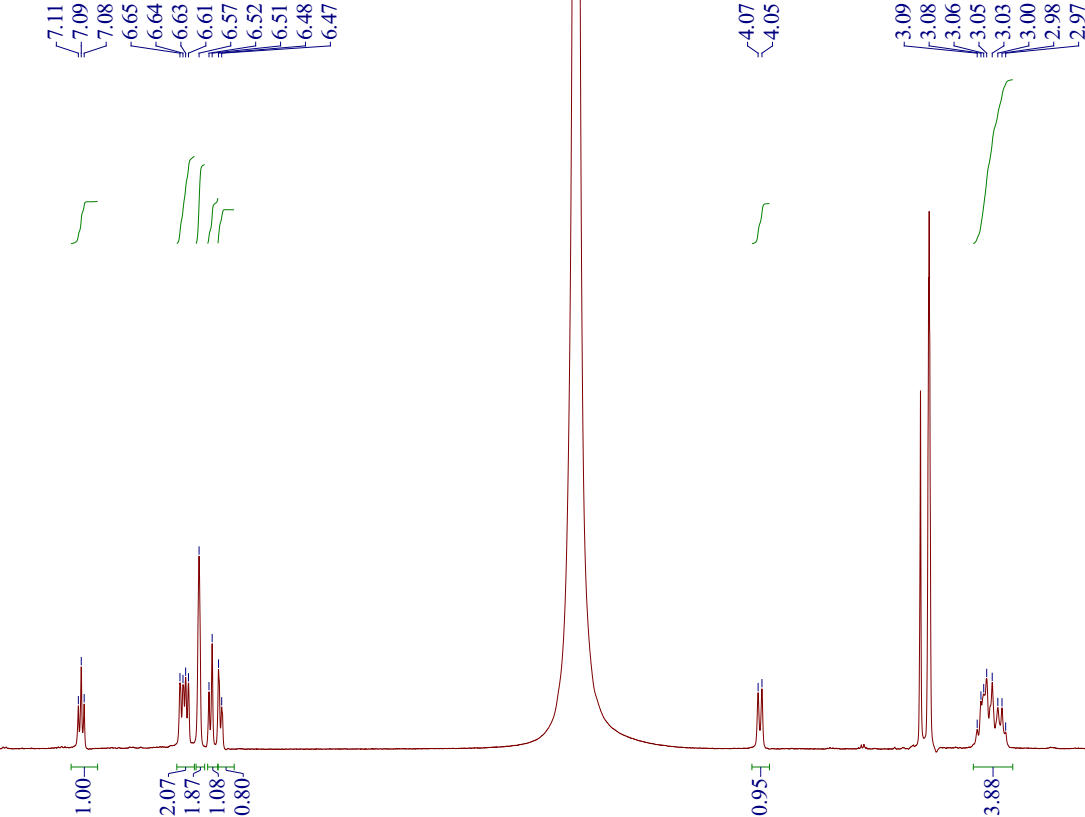


Figure S45. ^1^H NMR spectrum of **8** in methanol-*d*_4_


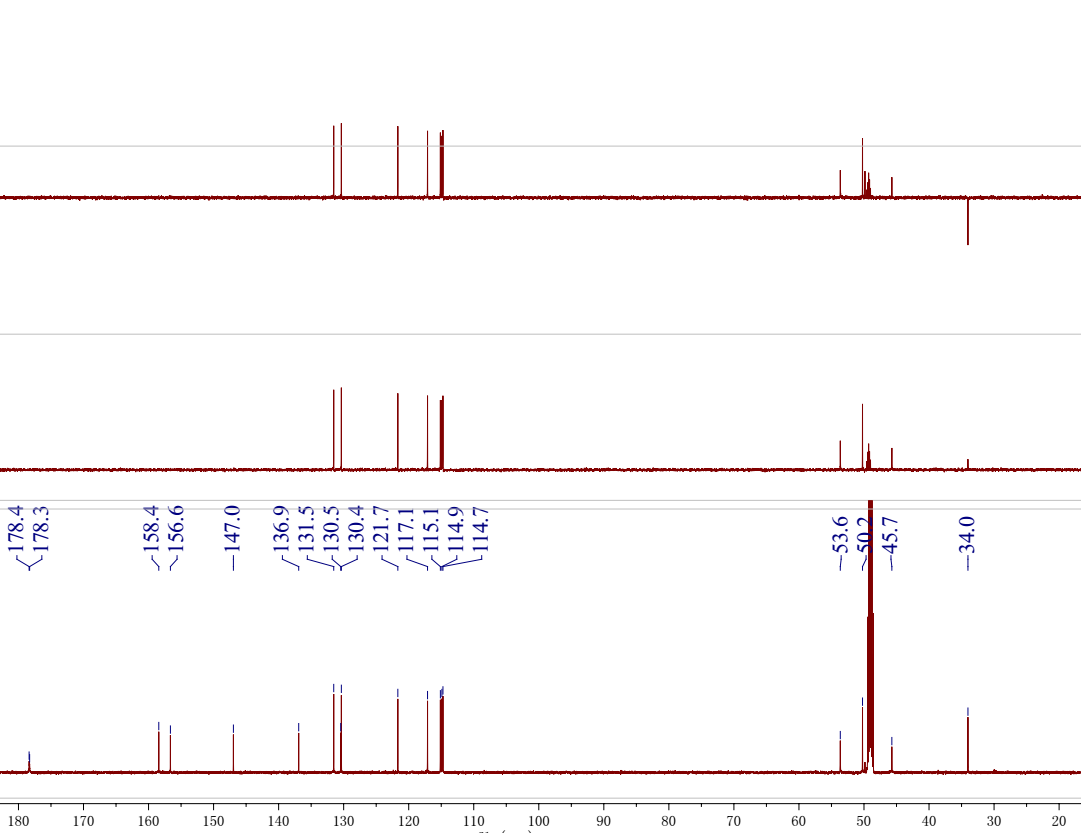


Figure S46. ^13^C NMR and DEPT spectra of **8** in methanol-*d*_4_


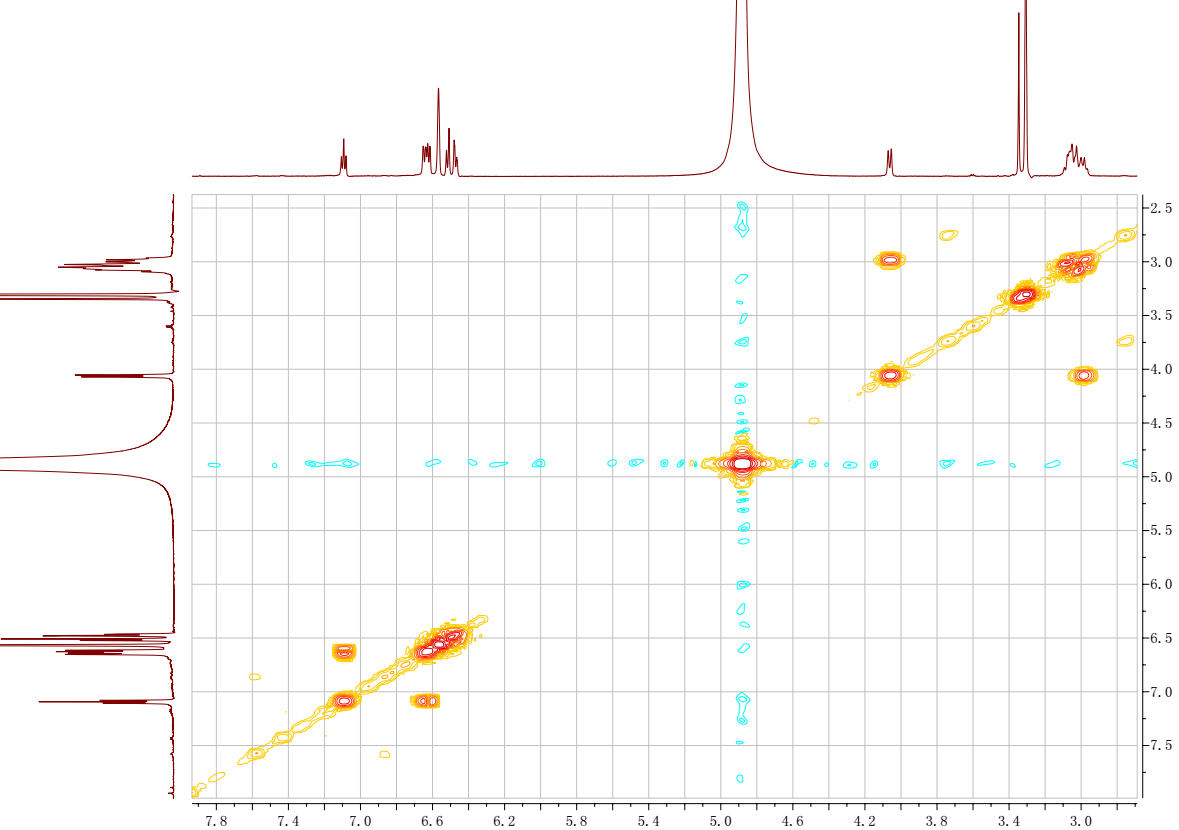


Figure S47. ^1^H-^1^H COSY spectrum of **8** in methanol-*d*_4_


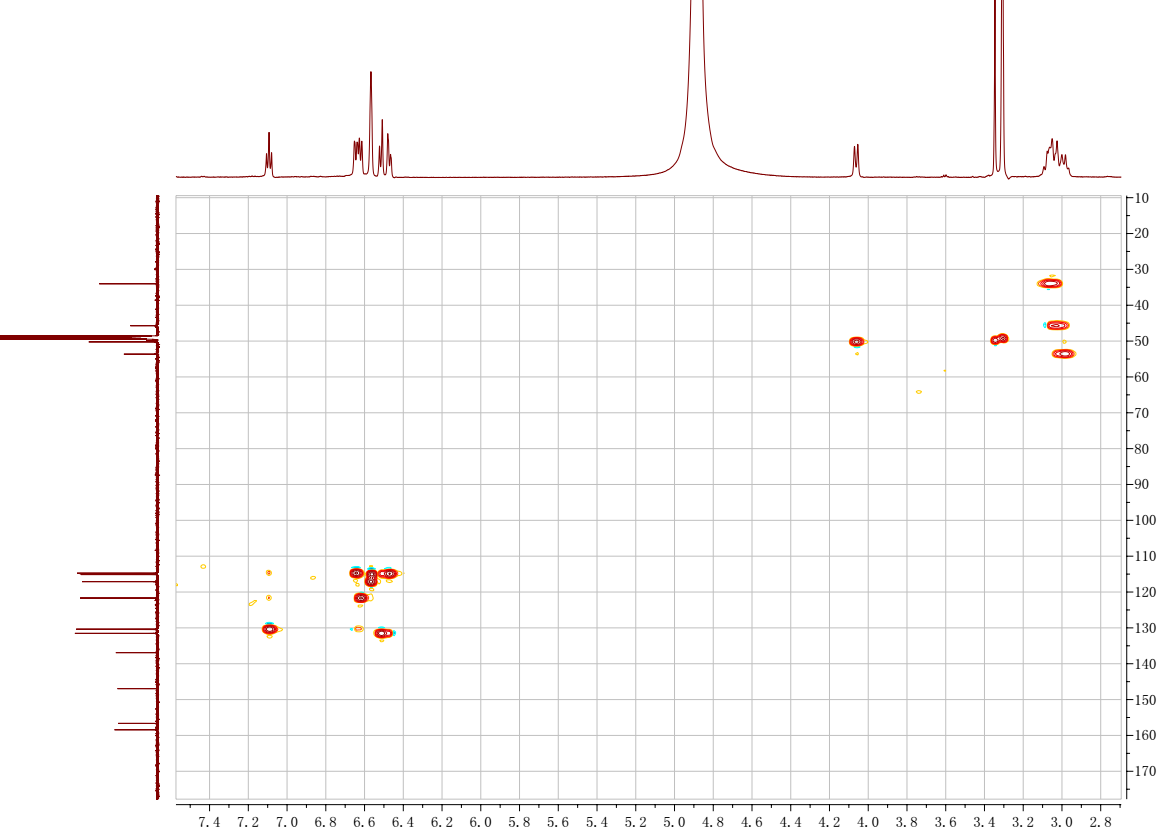


Figure S48. HSQC spectrum of **8** in methanol-*d*_4_


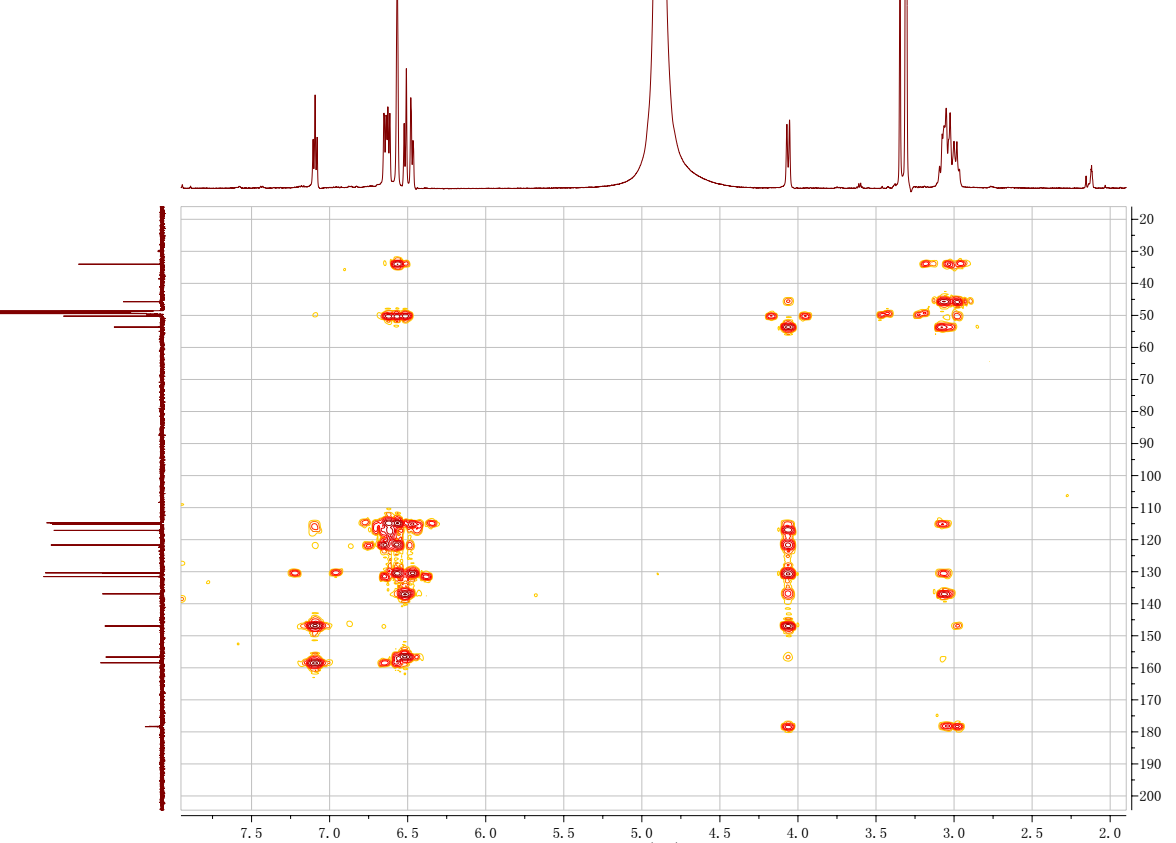


Figure S49. HMBC spectrum of **8** in methanol-*d*_4_


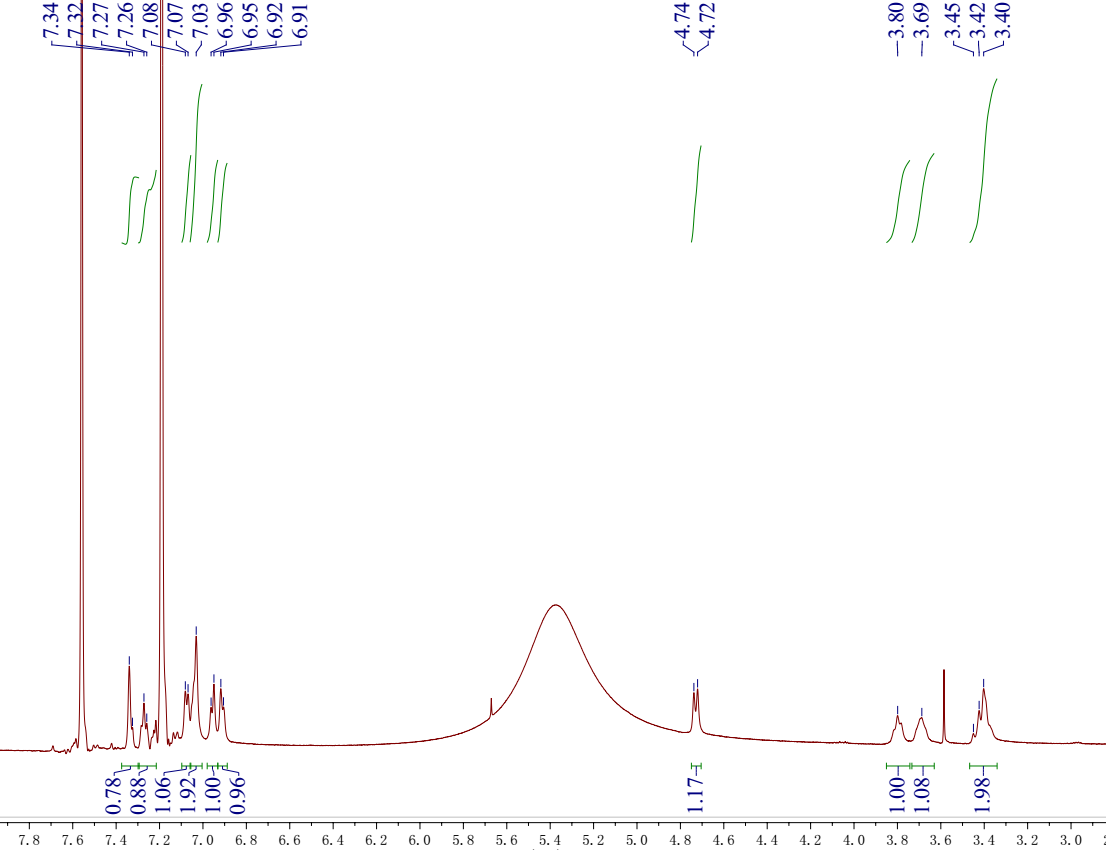


Figure S50. ^1^H NMR spectrum of **8** in pyridine-*d*_5_


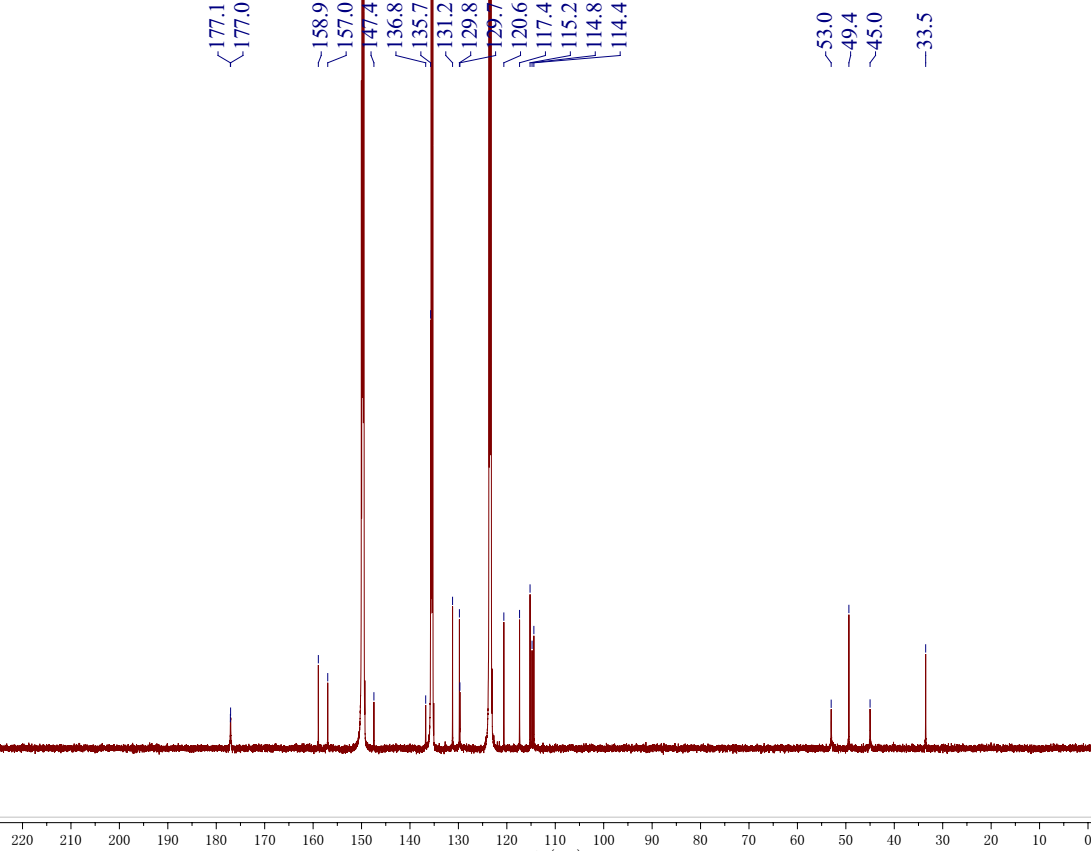


Figure S51. ^13^C NMR spectrum of **8** in pyridine-*d*_5_


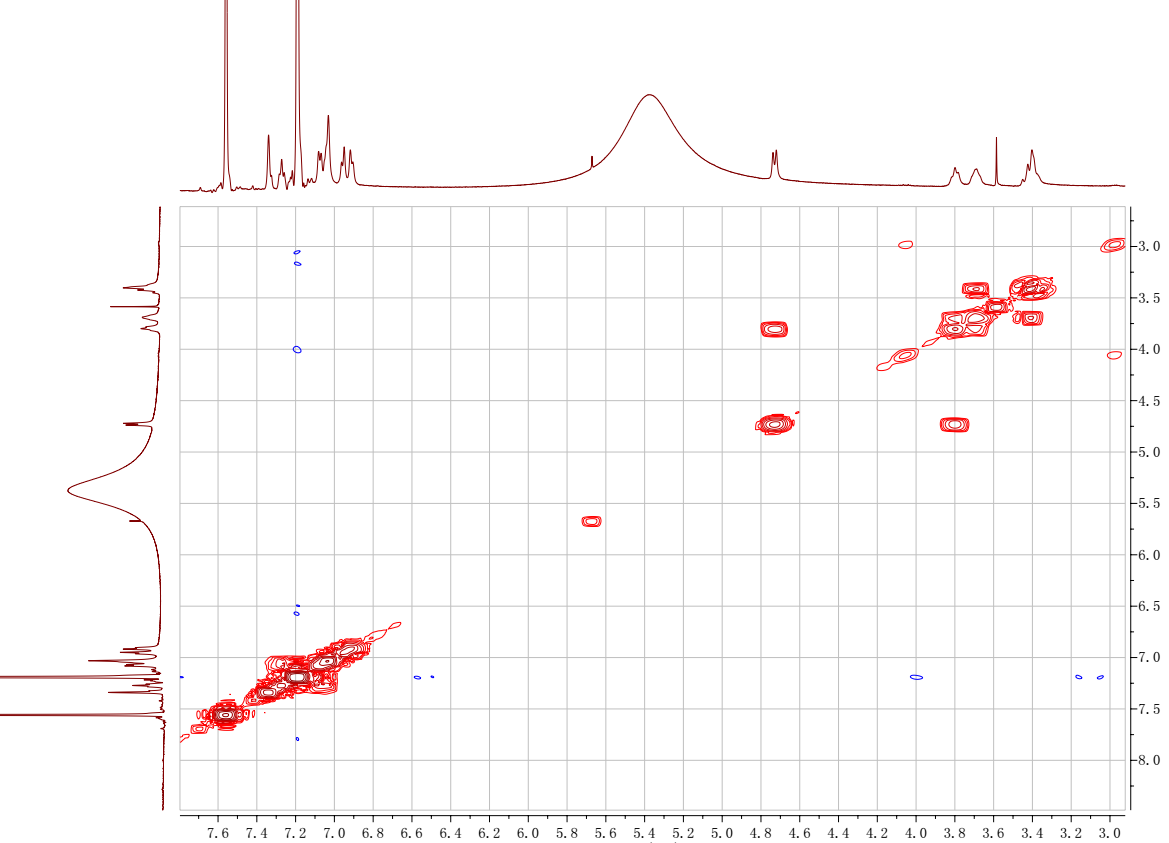


Figure S52. ^1^H-^1^H COSY spectrum of **8** in pyridine-*d*_5_


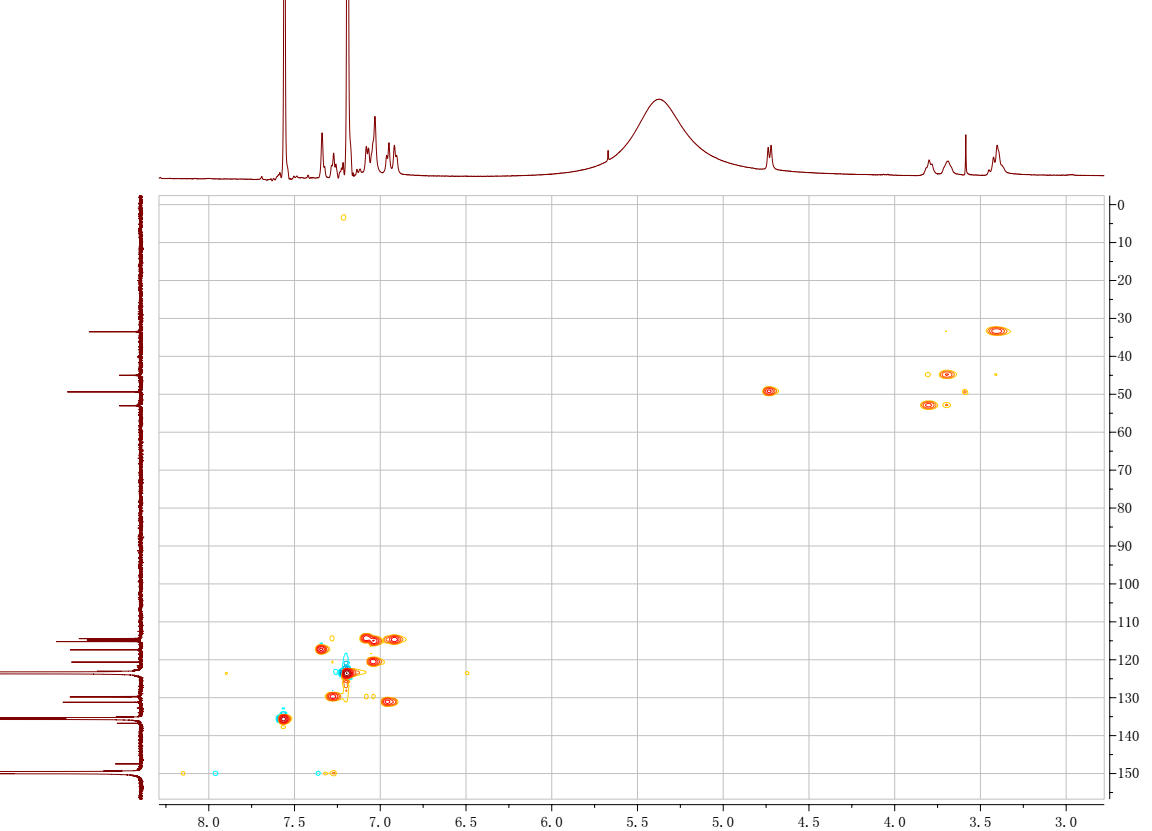


Figure S53. HSQC spectrum of **8** in pyridine-*d*_5_


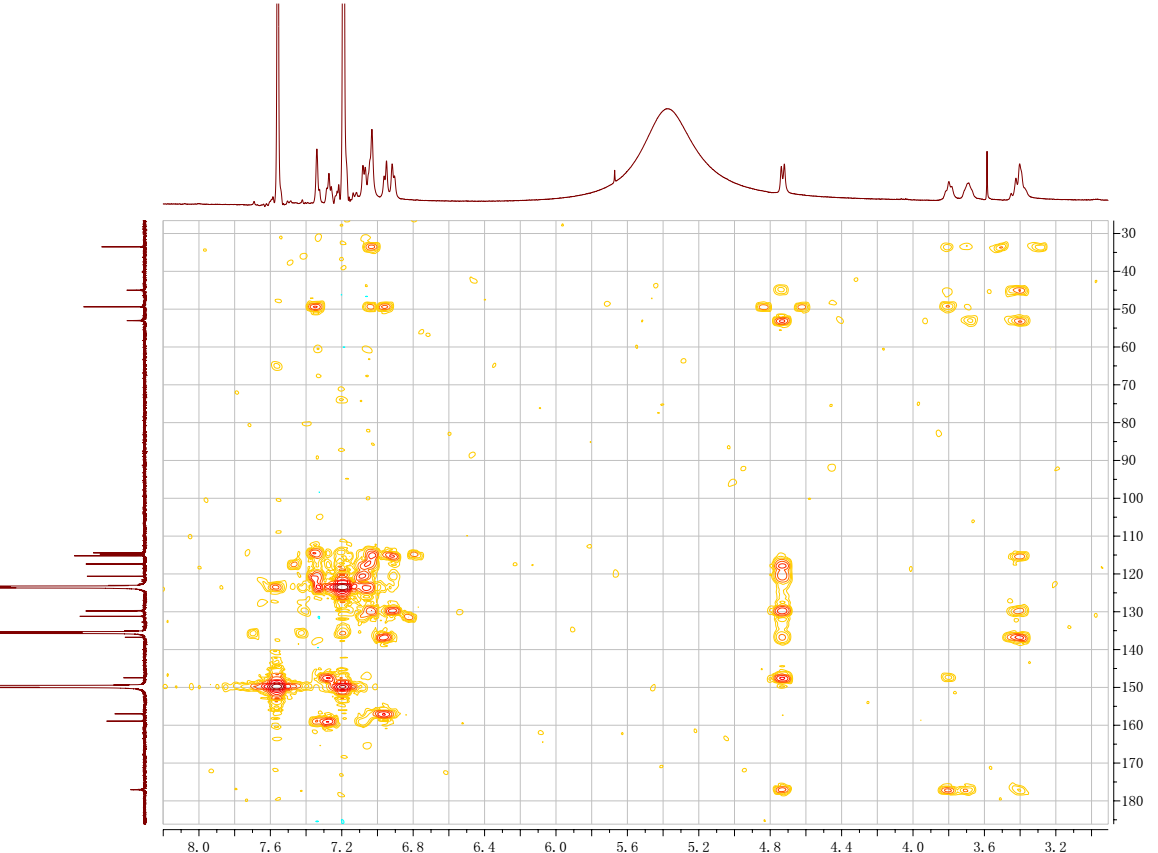


Figure S54. HMBC spectrum of **8** in pyridine-*d*_5_


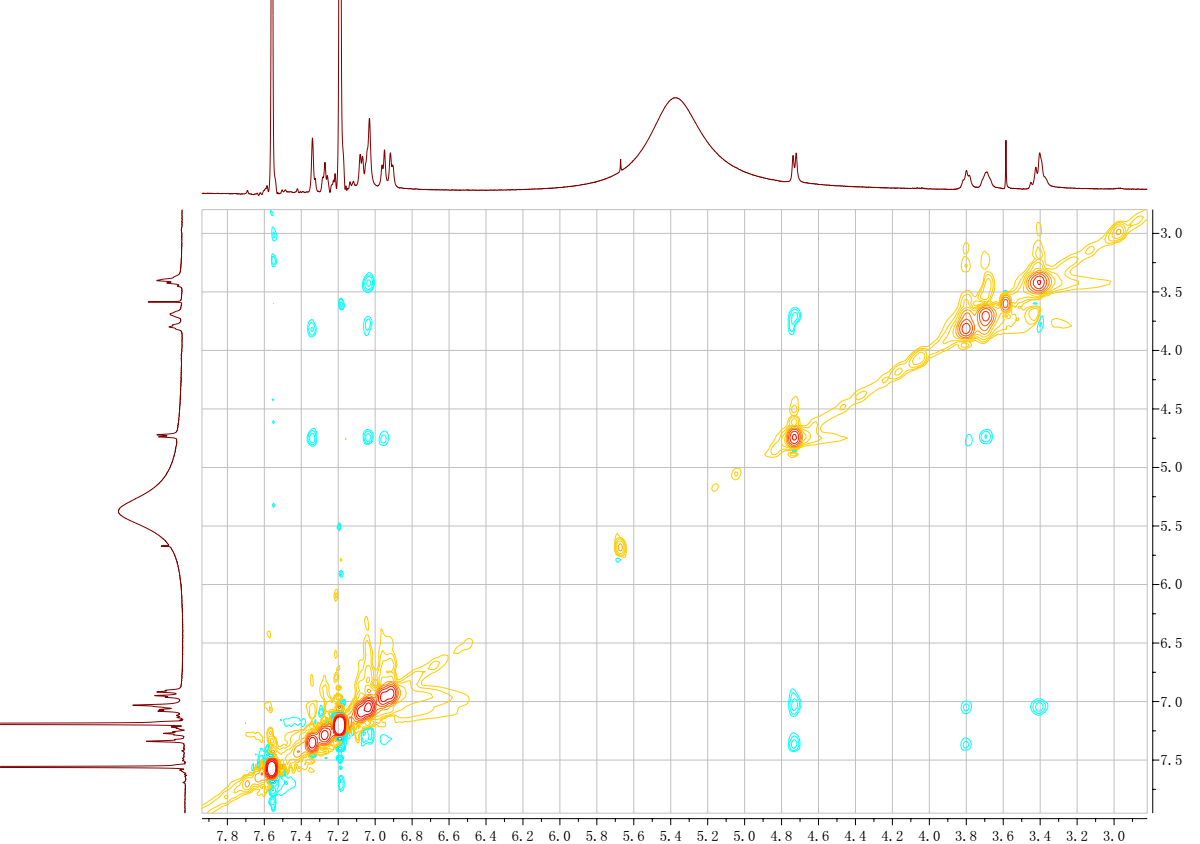


Figure S55. ROESY spectrum of **8** in pyridine-*d*_5_


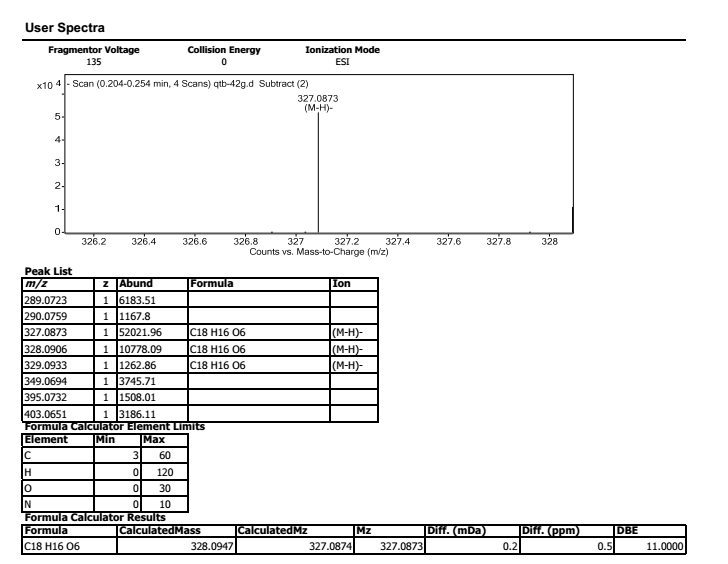


Figure S56. HREIMS of **8**


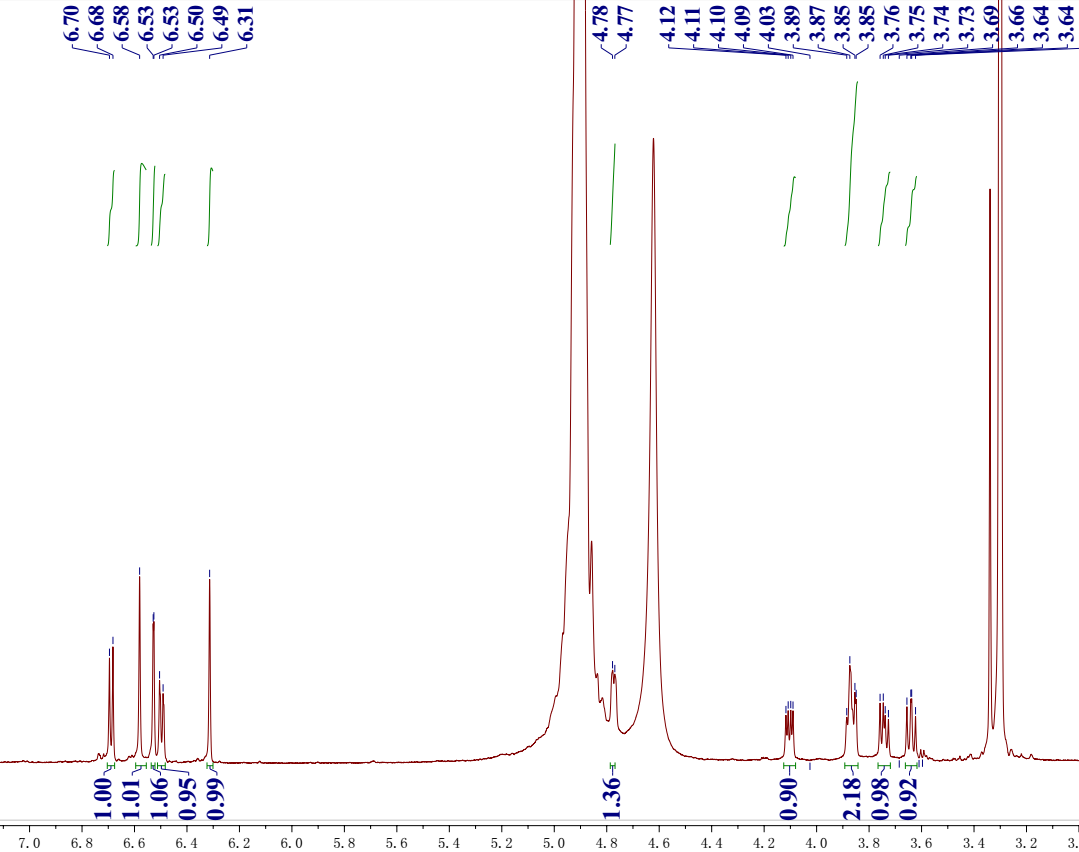


Figure S57. ^1^H NMR spectrum of **9** in methanol-*d*_4_


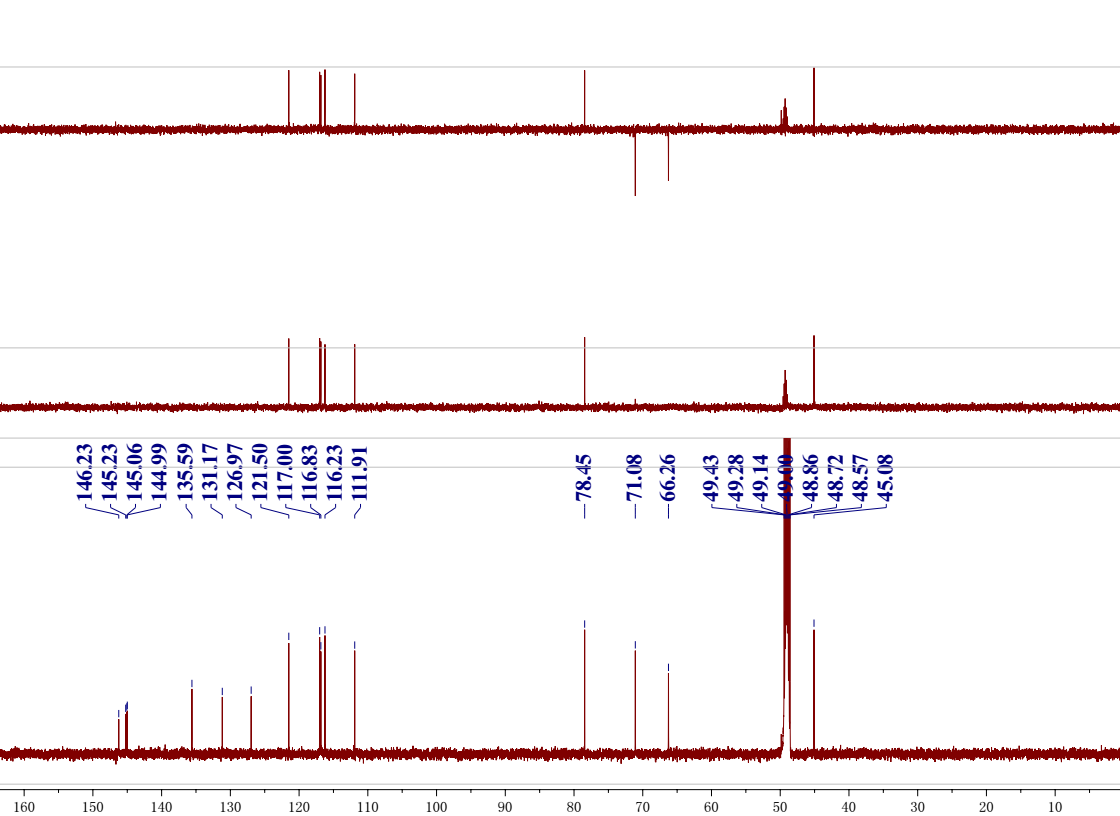


Figure S58. ^13^C NMR and DEPT spectra of **9** in methanol-*d*_4_


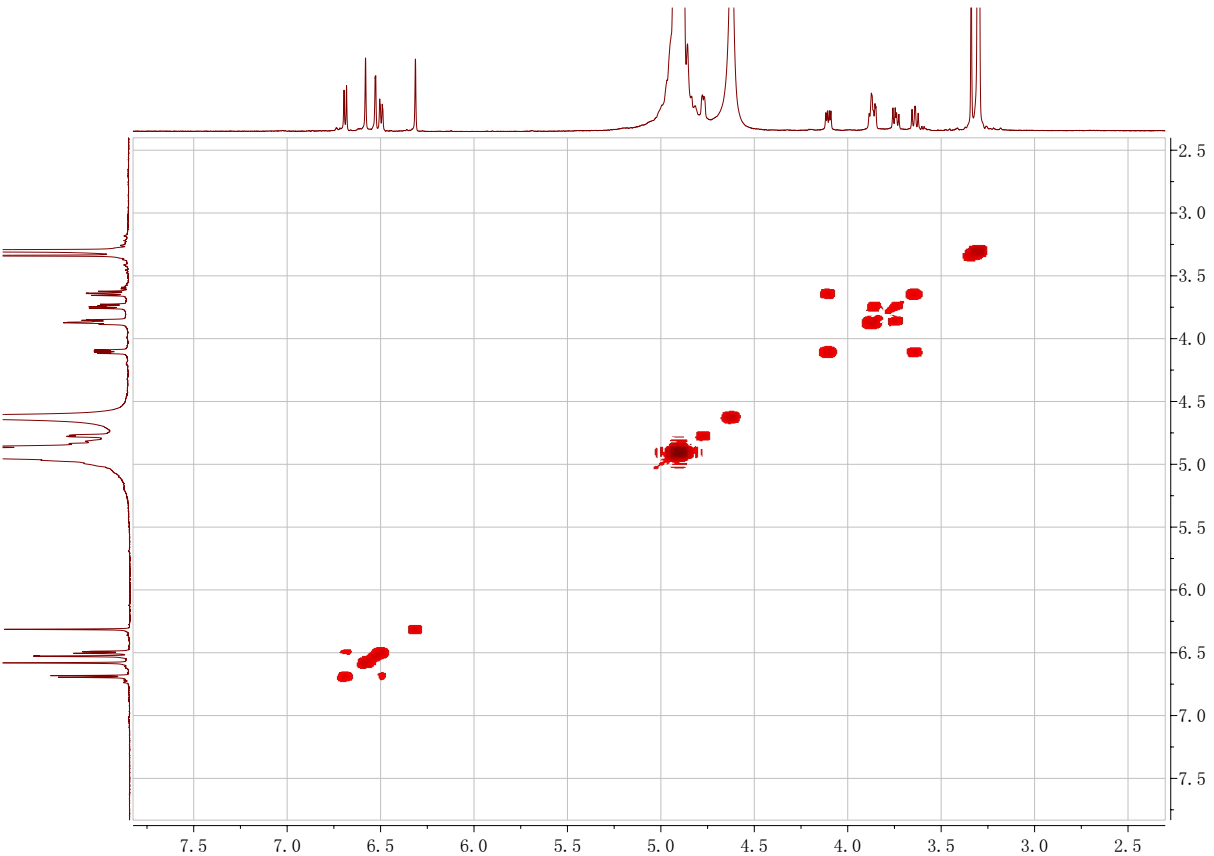


Figure S59. ^1^H-^1^H COSY spectrum of **9** in methanol-*d*_4_


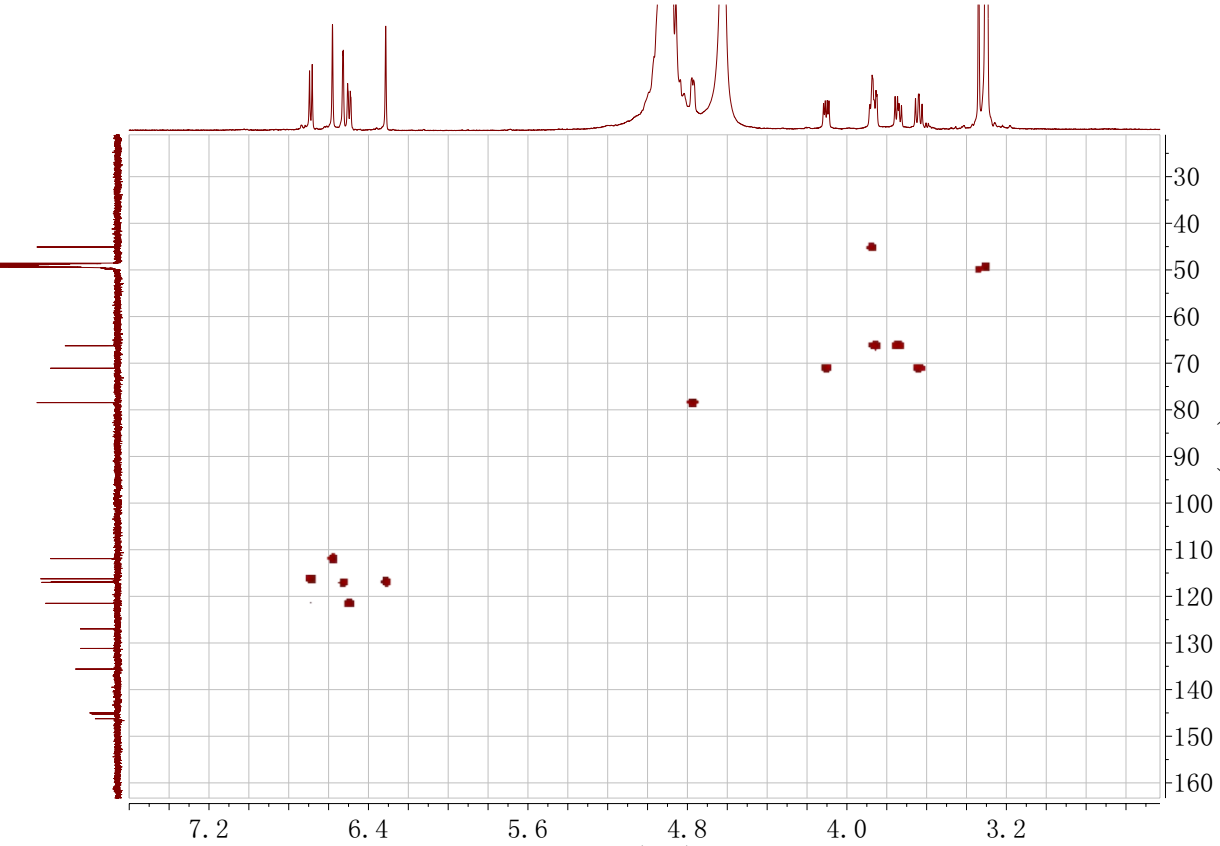


Figure S60. HSQC spectrum of **9** in methanol-*d*_4_


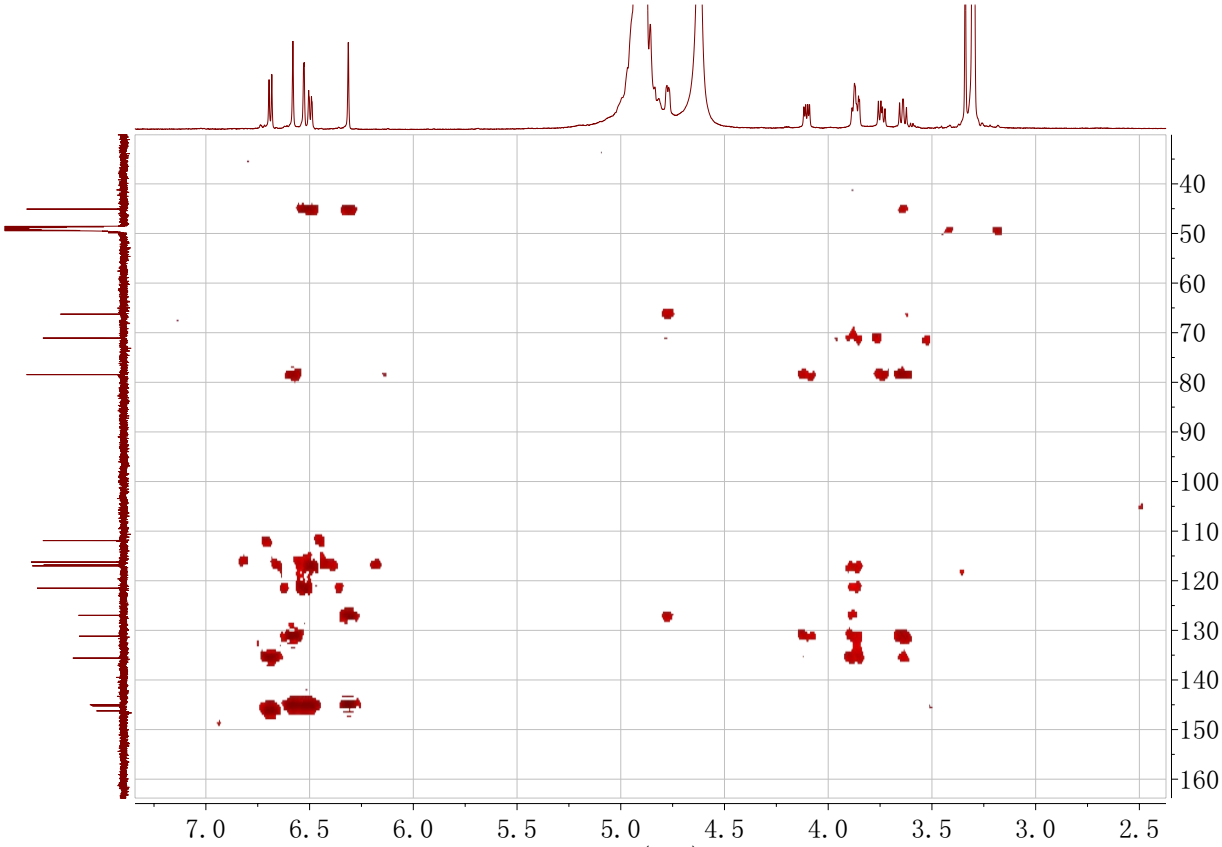


Figure S61. HMBC spectrum of **9** in methanol-*d*_4_


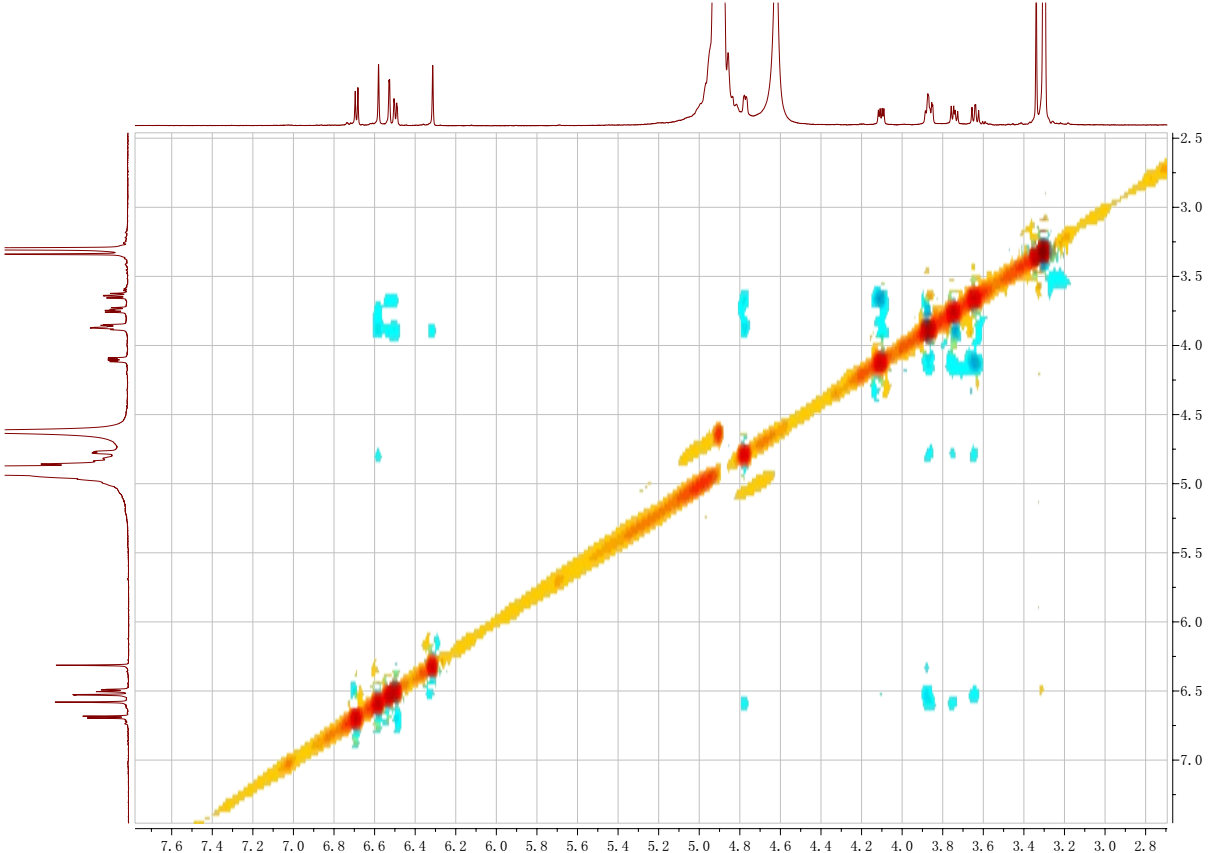


Figure S62. ROESY spectrum of **9** in methanol-*d*_4_


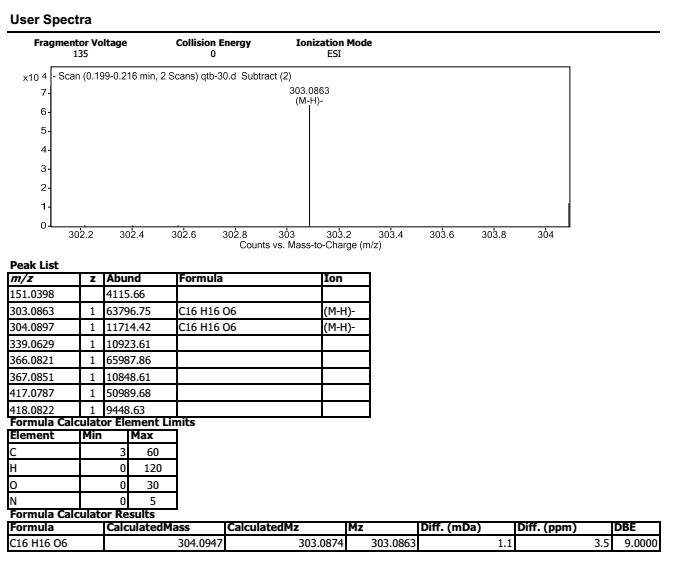


Figure S63. HREIMS of **9**
